# An Analytical Approach for Estimating Fossil Record and Diversification Events in Sharks, Skates and Rays

**DOI:** 10.1371/journal.pone.0044632

**Published:** 2012-09-05

**Authors:** Guillaume Guinot, Sylvain Adnet, Henri Cappetta

**Affiliations:** Institut des Sciences de l’Evolution de Montpellier, Université Montpellier 2, Montpellier, France; Technical University of Denmark, Denmark

## Abstract

**Background:**

Modern selachians and their supposed sister group (hybodont sharks) have a long and successful evolutionary history. Yet, although selachian remains are considered relatively common in the fossil record in comparison with other marine vertebrates, little is known about the quality of their fossil record. Similarly, only a few works based on specific time intervals have attempted to identify major events that marked the evolutionary history of this group.

**Methodology/Principal Findings:**

Phylogenetic hypotheses concerning modern selachians’ interrelationships are numerous but differ significantly and no consensus has been found. The aim of the present study is to take advantage of the range of recent phylogenetic hypotheses in order to assess the fit of the selachian fossil record to phylogenies, according to two different branching methods. Compilation of these data allowed the inference of an estimated range of diversity through time and evolutionary events that marked this group over the past 300 Ma are identified. Results indicate that with the exception of high taxonomic ranks (orders), the selachian fossil record is by far imperfect, particularly for generic and post-Triassic data. Timing and amplitude of the various identified events that marked the selachian evolutionary history are discussed.

**Conclusion/Significance:**

Some identified diversity events were mentioned in previous works using alternative methods (Early Jurassic, mid-Cretaceous, K/T boundary and late Paleogene diversity drops), thus reinforcing the efficiency of the methodology presented here in inferring evolutionary events. Other events (Permian/Triassic, Early and Late Cretaceous diversifications; Triassic/Jurassic extinction) are newly identified. Relationships between these events and paleoenvironmental characteristics and other groups’ evolutionary history are proposed.

## Introduction

Modern selachians (Neoselachii) represent a diversified clade of marine vertebrates encompassing all living sharks (about 500 described species) and batoids (rays and skates, about 630 described species) as well as some extinct groups. Known certainly since the Early Permian [1], neoselachians have developed a wide range of lifestyles, modes of reproduction and feeding strategies throughout their long and successful evolutionary history [2]–[4]. However, preservation of neoselachian remains in the fossil record is reduced due to the cartilaginous nature of their skeleton. In fact, neoselachian fossil remains mainly consist of isolated oral teeth (vertebrae, scales, fin spines and rostral teeth are also occasionally encountered) as a result of their polyphiodonty (continuous shedding and replacement of teeth), although exceptionally preserved skeletons are known from few localities [5]. Thus, taxonomic identifications and classifications almost solely rest on dental morphologies and the attribution of some taxa to higher taxonomic ranks is sometimes made difficult as a consequence of the reduced number of characters available (compared with whole skeletons) and of morpho-functional convergences. Nevertheless, its is accepted that teeth generally provide a set of morphological characters that frequently allow their identification at lower taxonomic ranks [5]–[6], commonly down to the species level.

The monophyly of the clade Neoselachii is now broadly supported by both morphological [7]–[9] and molecular data [10]–[15] and the hypothesis placing the extinct hybodont sharks as sister group to neoselachians [16]–[18] is likely. There are, however, various phylogenetic hypotheses (based on morphological and molecular data) suggesting different interrelationships within the Neoselachii (e.g. the position of Squatiniformes, Squaliformes, interrelationships among batoids) and no consensus has been found yet. For instance, the position of the clade Batomorphii remained problematic, with morphological studies [8]–[9], [19]–[20] indicating a derived position within the clade Hypnosqualea, a hypothesis largely inconsistent with stratigraphic data. All recent molecular studies [11]–[15], [21]–[23] clarified this issue, suggesting a basal position for the Batomorphii, as sister group to all living sharks (Selachimorpha).

Although selachian fossil remains are regarded as relatively common in comparison to other marine vertebrates, no attempts have been made to qualitatively assess the quality of the selachian fossil record, reported in various works [5], [24]–[26]. In addition, little is known about the events that have marked the evolutionary history of this group and recent studies either measured these over large geological periods using standing diversities [27] or focused on peculiar time intervals (Jurassic, K/T boundary), using evolution rates and/or subsampling methods [28]–[29]. The aim of the present study is to take advantage of the various and recent molecular phylogenetic hypotheses (years 2003–2012) in order to assess the fit of the selachian (i.e. neoselachians and hybodonts as used here) fossil record (recently updated by one of us [26]) to phylogenies according to different branching methods. This allowed an estimation of the diversity patterns of this group for orders, families and genera through time to be gained, along with the identification of various evolutionary events that marked this group over the past 300 Ma.

## Methods

### Phylogenetic Framework

The taxonomic level used is critical for the results obtained from palaeobiodiversity studies and the consequences of taxonomic levels used on resulting patterns and their corresponding issues have been discussed [30]–[32]. Although high taxonomic levels (orders, families) are little affected by problems related to their preservation, these are less informative for congruence-testing methods and the use of higher taxa obscures a large part of the phyletic and diversity patterns that would be observed with lower taxonomic levels. On the other hand, the use of species databases adds numerous issues concerning the fossil species concept (fossil species infrequently represent true biological species [33]), synonymies and others in addition to the fact that described fossil species represent only a small fraction of the genuine fossil species diversity [34]. Moreover, it has been shown that the genus is a much reliable rank on which biodiversity analyses are based [32], [35]. Consequently, the genus level will be the lowest taxonomic rank of the study presented here and higher taxonomic levels (orders, families) will be used for more general considerations.

**Figure 1:**
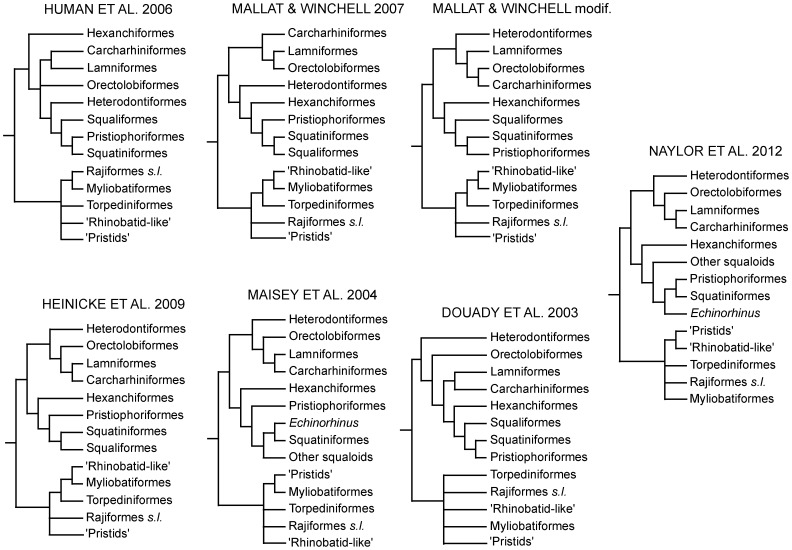
The seven phylogenetic hypotheses used in the analyses.

Due to the scarcity of comprehensive genus-based phylogenies of modern elasmobranchs (resolving phyletic relationships of sharks, rays and skates altogether), the phylogenetic framework used here is a compilation of different phylogenies found in the literature for each order, family and genus based on living taxa (see File S1). These intra-ordinal phylogenetic relationships were included within six cladistic trees corresponding to the six main phylogenies tested (Fig. 1) that comprise inter-order relationships found in recent molecular works devoted to neoselachians: DOU [11], MAI [3], HUM [21], HEI [15], MAW [14] and MAW-m [14] (with modification of shark interrelationships of [22]). A seventh recent molecular phylogenetic framework (NAY [23]) was considered as it provides genus-level neoselachian interrelationships. Following the common hypothesis that hybodont sharks are sister-group to all neoselachians [16]–[17], the former were included in the phylogenetic hypothesis in stem position, with intra-relationships following those of [17]. These order-level phylogenies, reduced to cladistic trees, were combined along with fossil taxa that were added in unresolved positions (polytomies) according to their systematic position [5], [26] and taxa of uncertain affinities were left in basal position in the order, super-order or higher taxonomic rank. Therefore, seven phylogenetic hypotheses were available for each taxonomic level (genus, family and order levels). Data on selachian fossil record comprising first and last occurrences of the recent and extinct taxa (513 genera, 89 families and 14 orders) included in this analysis is from [26]. See File S1 for detail on used phylogenetic hypotheses, File S2 for stratigraphic framework and File S3 for observed and inferred fossil record.

### Branching Methods

**Figure 2:**
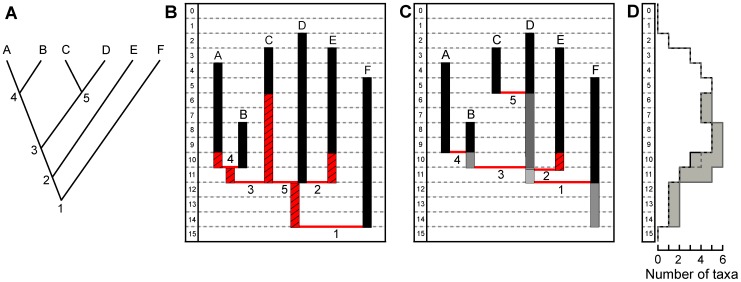
Two branching methods used for assessing the quality of the selachian fossil record and corresponding diversity. A: Cladogram showing the original phylogenetic hypothesis; B: stratigraphic ranges (black boxes) with ghost lineages (stripped red boxes) added to fit the phylogenetic hypothesis using the Conventional Branching Method (CBM); C: stratigraphic ranges with taxic interrelationships using the Direct Descendence Branching Method (DDBM); D: Resulting diversity curves, black: standing diversity; dashed grey line: inferred diversity using the DDBM; grey line: inferred diversity using the CBM; grey boxes: Genuine Diversity Domain (GDD).

Two possibilities of branching approximations can be considered for plotting fossil record on phylogenetic relationships drawn from a cladogram (Fig. 2A). The first one, referred to as ‘Conventional Branching Method’ (CBM) here (Fig. 2B), clearly respects cladistic rules and is broadly used in studies dealing with congruence-testing methods. According to this, sister groups originate from a common ancestor from which they subsequently diverge, thus implying a coeval origination age for the two lineages. Consequently, when such a condition is not observed in the fossil record (the origination age of one of the lineages is very often older than the other), the time gap between the two lineages must be ‘filled in’ in order to fit the cladistic hypothesis (Fig. 2A) and the fossil range added to the youngest lineage is referred to as ‘ghost range’ (Fig. 2B). In addition to the CBM, we considered another branching method (fig. 2C) that respects exactly the same phylogenetic relationships drawn from the same cladogram (fig. 2A) but differs in being thriftier in terms of added ‘ghost range’. Contrary to the CBM, this method considers that the divergence age of a lineage can be younger than the first occurrence date of its sister group and that the former can be descending directly from the latter. Taxa considered (branches) are regarded as pools representing a number of taxonomic entities of intergrading morphs that vary through time, but that are grouped together according to taxonomy and classification rules used in paleontological studies (typological concept). Accordingly, taxon A is branched directly from taxon B (Fig. 2C, ‘node’ 4) with no added stratigraphic range for the former. However, although all representatives of pool B are grouped together in the systematic conception, those of the oldest forms (grey box), can be considered as belonging to either A or B. Although this method seems to contradict the concept of clade in introducing paraphylies, this is justified here with supraspecific-level fossil taxa for providing a lowest estimated diversity especially considering the quality and nature of the selachian fossil record. Moreover this branching method does not artificially increase the amount of ghost ranges when dealing with groups of poorly resolved phylogenies (numerous polytomies), as in elasmobranchs. Similarly, although E diverges before D in the phylogenetic hypothesis, E seems to emerge from D (Fig. 2C, ‘node’ 2) even though the first occurrence of the former is younger than that of the latter. It is necessary however, that ‘node’ 2 is older than (or at the very least contemporaneous of) ‘node’ 3 and younger than ‘node’ 1, in order to respect the phylogenetic hypothesis induced by the cladogram, thus implying the addition of a ghost range at the base of E. This branching method is referred to as ‘Direct Descendence Branching Method’ (DDBM) here. Although it may allow for paraphylies *sensu stricto*, it can be considered that this branching method takes account of the variability of the taxonomic classification and respects the divergence order of each node proposed in the phylogenetic hypothesis, as opposed to the conventional method that retains and induces polytomies. Conversely, the CBM gives more credit to the phylogenies and requires the addition of numerous stratigraphic ranges for each clade to the detriment of the observed fossil record.

It is then possible to infer diversity curves (Fig. 2D) by compiling the number of taxa per time bin and corresponding ghost ranges, according to the overestimating (CBM) and underestimating (DDBM) branching methods. Thus, a domain constrained by the lowest estimated diversity values using the DDBM (lower border) and by the highest estimated diversity values using the CBM (upper border) can be identified (grey zone). This zone can be considered as a Genuine Diversity Domain (GDD), which should include ‘true’ taxic diversity values and the gap between standing diversity and GDD borders is indicative of the quality of the selachian fossil record. Thus, when such a gap is large, the fossil record is likely to be relatively incomplete, particularly if the lower border is much higher than the standing diversity (suggesting a lack of covering between last and first occurrences of two sister taxa). In addition, when DDBM and CBM diversity curves are superimposed, branching methods are likely to have little influence on index values, i.e. that the first occurrences of sister taxa are roughly coeval. Conversely, strongly diverging DDBM and CBM diversity curves suggest a large number of taxa of limited temporal distribution.

We used the Relative Completeness Index metric [36] for estimating the completeness of the selachian fossil record using both branching methods in order to compare estimated diversities according to the seven phylogenetic hypotheses considered. Because this metric considers the relative ratios of ghost and observed ranges, the lower the RCI score is, the better the fit of fossil record to phylogenetic hypotheses. We use the terms RCI and RCI’ in order to differentiate the calculations using the conventional branching method (used in RCI *sensu* [36]) from the branching method using direct descendence, respectively. For each hypothesis and taxonomic level, diversity curves and scores (RCI, RCI’) were computed for unresolved trees that retain all the polytomies induced by the phylogenetic uncertainty and/or addition of fossil taxa with unresolved phylogenetic relationships. Subsequently, RCI and RCI’ values were also computed for resolved trees where polytomies were randomly resolved using routine replications in order to reduce the amount of ghost ranges. We retained the lowest RCI and RCI’ scores indicating the best fit of resolved phylogenetic relationships to fossil record.

All data analyses were made using the R Statistical software [37] with the package APE [38]. Programming codes are available upon request to the authors.

## Results

### Fit of Phylogenies to Stratigraphy

RCI and RCI’ scores computed for unresolved trees (Table S1) according to each hypothesis and taxonomic level are obviously significantly higher than corresponding RCI and RCI’ values for resolved trees, respectively. Similarly, RCI scores are lower for data using higher taxonomic ranks as a result of the smaller number of taxa encompassed, thus limiting the amount of ghost lineages (e.g. compared with generic data). Routine replications performed to randomly resolve polytomies induce a great number of RCI and RCI’ values (with minimal values reached for few computed resolved trees, see Figure S1). For each analysis, we retained the lowest RCI and RCI’ values for resolved trees, bearing in mind that minimal values depend on the fixed number of replications. However, the computed phylogenetic relationships that best fit the fossil record have no real biological meaning and only attest of the minimal value allowed by the phylogenetic framework and fossil record (as opposed to the calculation of GER where only the fossil record depends on the minimal value of gaps, [39]). Moreover, the minimal RCI and RCI’ values for resolved trees are dependent on the number of replications performed and must be considered with caution when the number of possible resolved trees reaches infinite values (i.e. generic data). Thus, only RCI and RCI’ values for unresolved trees will be retained for further comparisons and discussion.

Results suggest that three out of seven phylogenies stand out: MAI [3], HUM [21] and DOU [11] phylogenies received the best RCI/RCI’ scores, depending on taxonomic levels considered. No correlations were evidenced by statistic tests (see Table S2) between RCI and RCI’ values for order and family data, thus suggesting that branching methods influence RCI scores’ distribution at higher taxonomic levels. Conversely RCI and RCI’ values appear correlated for genus data. The other correlation observed (RCI scores for family data vs. RCI scores for order data) may be explained by a roughly similar amount of ghost ranges between family and order data for a given tree topology (the fossil record for a given order is often represented by a single family). One can expect that the resolution of tested phylogenetic hypotheses (number of tree nodes) seriously influences RCI and RCI’ values. However, RCI and RCI’ values do not appear correlated to the number of tree nodes in most cases (see Table S2), with the exception of two correlations, although the one where the number of tree nodes is positively correlated to RCI scores is not strongly statistically supported. In addition, the branching method used does not appear to influence this relationship as such a correlation was found for both branching methods. Giving the inconsistency in their distribution, these correlations remain difficult to interpret.

### Quality of the Selachian Fossil Record

**Figure 3:**
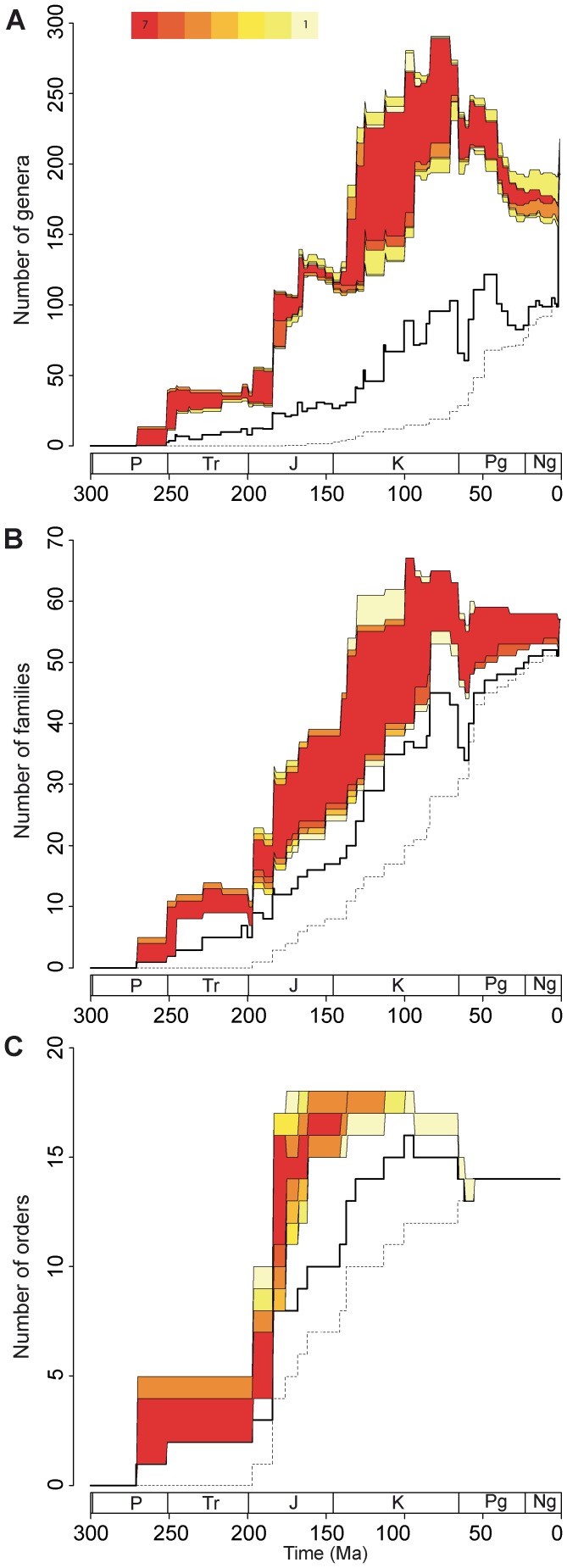
Inferred taxic diversity for A: genera, B: families, and C: orders. Colored zone corresponds to the Genuine Diversity Domain (GDD), with the upper boundary represented by CBM diversity values and lower boundary represented by DDBM values, for the seven phylogenetic hypotheses considered. Color nuances within the GDD are function of the number of phylogenetic hypotheses covering the GDD (see figure). Bold black line: standing diversity; dashed line: diversity of modern taxa with representatives in the geological times. Geologic interval abbreviations are as follow: P  =  Permian; Tr  =  Triassic; J  =  Jurassic; K  =  Cretaceous; Pg  =  Paleogene; Ng  =  Neogene.

All generated diversity curves were plotted (Fig. 3) for the three datasets used (orders, families, genera; see File S3) along with modern taxic and observed diversity (standing diversity) curves.

Figure 3A illustrates these results for genus data. These indicate that the GDD is rather well constrained (close upper and lower borders) since the Permian until the Jurassic/Cretaceous and to a lesser extent in the Cenozoic, suggesting that apparitions of sister taxa in the fossil record are more or less contemporaneous, whereas the Cretaceous interval (particularly Early Cretaceous) shows the greatest uncertainty (depending on the branching methods and phylogenetic hypotheses).This suggests that a large number of phylogenetic relationships of Cretaceous genera are unresolved, that numerous genera must be reconsidered and/or that their stratigraphic occurrences are too restricted in time. With the exception of the early Jurassic interval, the gap between standing generic diversity and GDD values is large from the Triassic until Recent. This is particularly marked for the Late Cretaceous-Middle Eocene interval where observed fossil data represent down to nearly 30% of the estimated diversity (considering values of the lower GDD boundary). Patterns obtained from family-based datasets (Fig. 3B) differ in some ways. While the GDD is narrow in the Permian-Early Jurassic interval, the gap between resulting curves for CBM and DDBM is marked throughout most of the Early Jurassic – Neogene interval. Consequently, the family-level selachian fossil record can be regarded as globally complete if one considers curves generated from DDBM branching hypothesis (lower GDD boundary), with the exception of the latest Cretaceous – earliest Paleogene interval. However, the CBM suggests that the family diversity is poorly known in the whole Jurassic – Paleogene interval, with up to about half of the diversity yet to be discovered. With the exception of the late Jurassic – lower Cretaceous interval (Fig. 3C), the gap between the lower GDD boundary and standing ordinal diversity is reduced, indicating a reasonably good fossil record for this taxonomic rank (although this gap is greater in the pre-mid Jurassic if one considers the upper GDD boundary). The thickness of the GDD, although important in the Early – Middle Jurassic interval, indicates rather well constrained estimated diversity values regardless of the branching method used, a pattern that is possibly imputable to the limited branching possibilities for orders in comparison to lower taxonomic ranks, due to the smaller number of taxa considered. Despite the efforts put to infer diversity patterns for high taxonomic ranks (orders, families), these results should be taken with caution because high taxonomic ranks are less informative than the genus level (see Method Chapter and references herein). This is particularly true for some early Mesozoic selachians taxa of which affinities at higher taxonomic levels are uncertain.

### Major Events in the Selachian Evolutionary History

**Figure 4:**
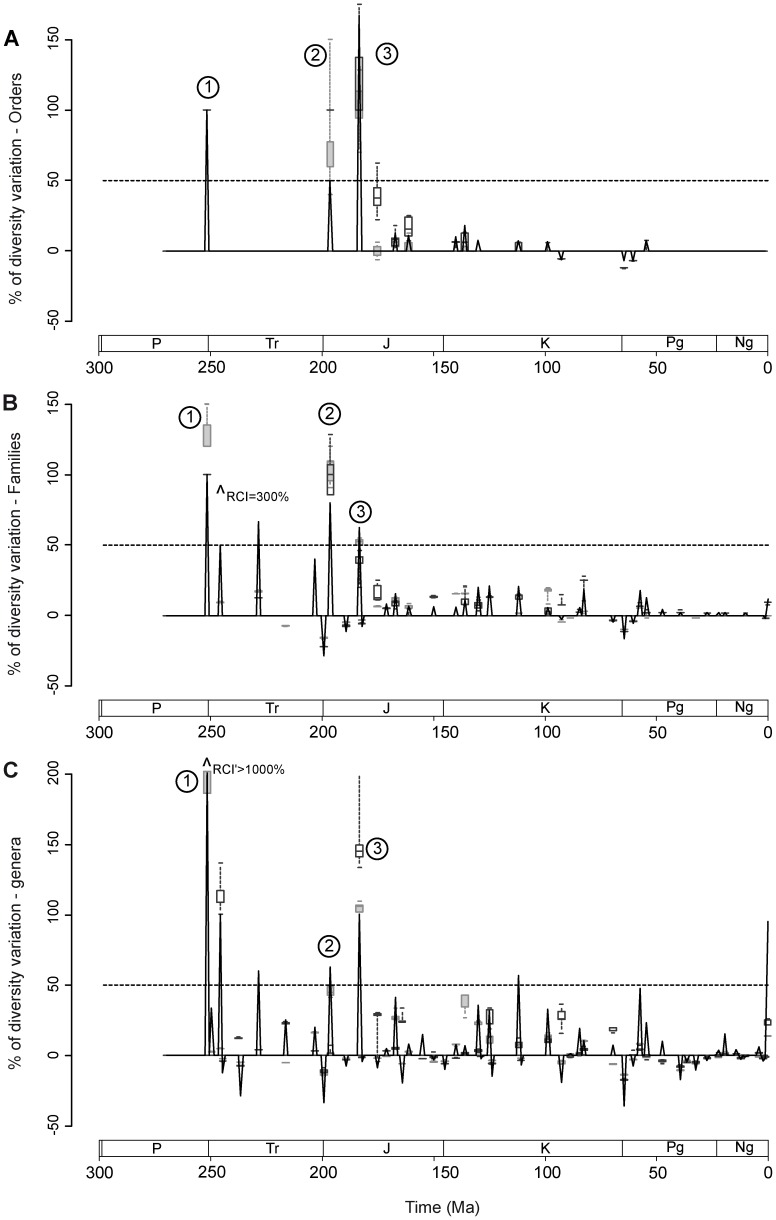
Percentages of diversity variation through time for A: orders, B: families and C: genera. Black solid line indicates diversity variations computed for standing diversity. Filled grey boxes and empty black boxes indicate ranges of diversity variation percentage computed with CBM and DDBM, respectively. Percentages falling outside the graph are indicated by arrows and corresponding percentage values.

Percentages of diversity variation through time were computed (Fig. 4) using observed and inferred fossil record, with the usual formula: 100(N_t+1_ – N_t_) / N_t_ [N_t_  =  number of taxa at time t]. Diversity events are represented by positive or negative peaks with corresponding boxes indicating the range of variation according to the phylogenetic hypotheses, for a given branching method. Thus, when the observed diversity variation is situated below the inferred values, the diversity event is expected to be underestimated by the observed standing diversity, and conversely. Again, timing and amplitude of these events vary according to the branching method used and phylogenetic hypothesis considered, respectively. Basically, inferred diversification events are coeval between CBM and DDBM when first occurrences are simultaneous in the fossil record, whereas when gradual, diversification ages inferred from CBM are older than those inferred from DDBM.

Three major diversification events (arbitrarily above 50%) can be identified from each of the three datasets (genera, families and orders). The first one (1) is a marked diversification around the Permian/ Trias boundary, the three datasets indicating a minimum diversity increase of 100% (over 1000% with DDBM for generic data). However, inferred ordinal CBM values do not support a diversification at this time, but earlier (this branching method assumes a coeval origination for most of neoselachian clades). Indeed, a mid Permian diversification is suggested by all datasets but diversification rates cannot be calculated for this, as a result of the edge effect [40]. A second diversification event (2) is observable in the earliest Jurassic (Hettangian/ Sinemurian). Although this is marked for all datasets and branching methods, the observed diversity increase appears underestimated for both ordinal and familial data, but overestimated for generic data. The last diversification event (3) (Pliensbachian/ Toarcian) is of higher or similar amplitude according to the datasets, and recognized regardless of the datasets and methods considered. Two additional diversification events are observable from observed familial and generic data: the Lower/Middle Triassic boundary and the Middle/ Upper Triassic boundary. However, although the former is supported by inferred values, branching methods diverge concerning its amplitude. Similarly, the latter event appears overestimated by observed values as DDBM and CBM values either suggest a minor diversification event or no event. The Cretaceous period is characterized by a series of moderate to minor evolutionary events rather than a single major one. The tempo of these evolutionary events slightly differs according to branching methods. CBM suggests a marked diversification (genera and families) in the earliest Cretaceous followed by a series of diversity peaks in the Cretaceous, including a peak in the Cenomanian. However, generic diversity values inferred from the DDBM indicate a gradual increase until the earliest Late Cretaceous (according to the phylogenetic hypotheses considered), where a marked diversity peak is present (Cenomanian; 30–40% diversity increase), followed by another distinct peak in the Campanian/ Maastrichtian (20% increase). DDBM on family dataset suggests a succession of stepwise increases throughout the Cretaceous with a marked peak at the Santonian/ Campanian boundary (around 25% increase). In addition, two diversity peaks represented in observed data (Albian and late Paleocene) appear overestimated when phylogenetic hypotheses are considered. It should be noted that, although the Cretaceous/ Paleogene extinction is represented in the three datasets, its amplitude appears overestimated in observed data (e.g. 12–15% inferred vs. 40% observed diversity drop for generic data), which is due to the higher general diversity estimated by phylogenies. This extinction is thus comparable, at genus and family levels, to another diversity drop observed for both datasets at the Triassic/ Jurassic boundary. Other diversity drops observed for both datasets remain largely overestimated by observed data and/or represent minor events. Finally, inferred Cenozoic ordinal and familial diversities stagnate or increase slightly, as opposed to inferred generic diversity values that show a series of minor late Eocene – Oligocene drops followed by roughly constant values until Recent. Obviously, the peak observed for genus and family data at recent times appears largely overestimated, regardless of the branching method used.

## Discussion

A common hypothesis concerning neoselachian phylogenetic interrelationships is the basal dichotomy between the shark clades Galeomorphii (Orectolobiformes, Carcharhiniformes, Lamniformes, Heterodontiformes) and Squalomorphii (Hexanchiformes, Squaliformes, Pristiophoriphormes, Squatiniformes). Globally, RCI and RCI’ values do not clearly indicate if the fossil record supports this basal dichotomy of sharks represented in four of the seven hypotheses (e.g. MAI [3], HEI [15], MAW-m [14], [22], NAY [23]). Even though one of them, MAI [3], received some of the best scores at family and order levels, those of DOU [11] that only supports the Squalomorphii clade, received the best scores for RCI and RCI’ for genus data. This incongruence remains unclear and can testify: (1) of the incompleteness of the fossil record, especially during the first steps of the neoselachian radiation, (2) of irrelevant resolutions of phylogenetic relationships, or (3) a combination of these two factors. Actually, the main difference between the latter phylogenies and the former lays is the resolution of interrelationships among batoids. While this group is well constrained in HEI [15] and MAW-m [14], [22], there is an important polytomy in MAI [3]. Similarly, the other phylogenetic hypothesis that best fits the fossil record (RCI for family and order data) is HUM [21]. This considers that the Hexanchiformes are sister-group to all sharks and the clades Galeomorphii and Squalomorphii are fragmented into a clade gathering Carcharhiniformes and Lamniformes, a second clade with Heterodontiformes, Squaliformes, Pristiophoriformes and Squatiniformes, the Orectolobiformes being placed in polytomy between the two former. Again, although this shows a good fit to stratigraphy, a possible reason for the low RCI values attributed to this phylogeny, along with the position of the Orectolobiformes, is the relationships among the clade Batomorphii, with the clade Rhiniformes, Prisids and Torpediniformes being placed in unresolved position. It is thus likely that this topology received low RCI scores with CBM because it follows the stratigraphic order of apparition of shark clades and leaves a degree of freedom concerning the batoid interrelationships. In addition, there is a possible relationship between branching methods and phylogenies for high taxonomic ranks, the tree topologies including the groups Galeomorphii and Squalomorphii being concordant with the DDBM whereas the topology of HUM [21] is in agreement with the CBM. However, DOU [11] received best RCI and RCI’ scores for genus data (although other phylogenies received very close index values), suggesting that branching methods do not influence congruence values at lower taxonomic ranks. Again, the large polytomy among the batoid clade most likely is responsible for these low congruence index values.

However, and although the fossil record for orders appears rather complete with the exception of the Middle – Late Jurassic interval, results indicate that standing diversity of lower taxonomic ranks (families and to a greater extent, genera) is by far imperfect. This may be regarded as contradictory to the fact that selachian fossil remains have been collected and studied for over two centuries. However, such a gap between observed and inferred diversities is conceivable as studies based on bulk-sampling and washing-sieving techniques became common only about fifty years ago, whereas older studies used surface-sampling of conspicuous remains exclusively. On the basis of the high number of Lazarus taxa, the Mesozoic is considered a period of corresponding poor fossil record for neoselachians [27], a pattern also observed here for selachians in general (including here neoselachians and hybodonts). This is particularly true for the Jurassic interval, where both branching methods on family and genus data indicate large gaps between standing and inferred diversities. This is likely to be the result of the geographical restriction of studies on Jurassic selachians, being almost exclusively limited to European localities (mainly Germany and England). Similar remarks can be made, to a lesser extent, for Early Cretaceous genus and family data (with the exception of family data using DDBM) but the low diversity of sampled paleoenvironmental facies may also be responsible for such a gap. These two parameters, along with the uncertainty of affinities of numerous taxa, are also likely to influence results for Triassic diversity. Causes for differences between observed and inferred generic diversities in the Late Cretaceous and mid-Eocene are less straightforward. Numerous corresponding faunas have been reported from a variety of facies, geographical areas and environmental realms. However, this period corresponds to the highest inferred selachian diversity and thus, sampling effort may simply not have been sufficient to cover such a diversity.

Few recent studies, restricted to the Mesozoic interval, have attempted to identify key events in the selachian evolutionary history. Recent studies suggested a diversification peak in the Toarcian (late Early Jurassic) [29] as well as a significant neoselachian diversification in the Early Jurassic and possibly Middle Jurassic and a second, larger phase of diversification through the mid to Late Cretaceous [27], [41]. Results presented here however, indicate a phase of radiation in the mid Permian. This should be taken with caution as timing only depends on the age of the oldest neoselachian remains known to date (‘*Synechodus*’ *antiquus* [1]) and further discoveries may modify this assumption. The second diversification event inferred at both genus and family levels takes place around the Permian/ Triassic boundary. Little is known about pre-Triassic neoselachian and hybodont evolution patterns but it appears that this period corresponds to the first major radiation for these groups and it is noteworthy that most of the early shark groups went extinct by the late Permian (e.g. bransonelliforms, stethacanthids, symmoriids, petalodontiforms). It is thus likely that this radiation was an opportunistic response to the extinction of early shark groups, with neoselachians and hybodonts probably filling up ecological niches previously occupied by the former. The Triassic shark diversity plateau is followed by two major Early Jurassic diversification events (≈ Hettangian and Toarcian) that have been reported from previous studies using different methods. Neoselachian diversifications in the Rhaetian [42] and Hettangian [27] have been signaled as well as a diversity peak in the Toarcian [29]. In these cases, authors suggest a correlation between rising sea-levels [29], [42], warm climatic periods [29] and increasing diversity. In addition, innovations in body plans and reproduction strategies (oviparity) were mentioned as possible adaptations that favored Early Jurassic selachian radiations [29]. Although these parameters are likely to have played a role in these Early Jurassic selachian evolutionary events, the fact that these events are contemporaneous to the radiation of actinopterygian bony fishes in the Late Triassic and early Jurassic is striking [43]. Neoselachian adaptations to active predation (jaw suspension, sensory system, vertebra morphology) are key characters/ structures that allowed predation on diversifying early Mesozoic ray-finned bony fishes. It is difficult to assess whether the Cretaceous diversifications took place during a rapid pulse in the early Cretaceous followed by minor diversification events in the Late Cretaceous (as suggested by CBM) or during the Early, Mid and latest Late Cretaceous (as suggested by DDBM). Reason for this is that very little is known on (particularly pre-Aptian) Early Cretaceous fully marine selachian faunas. Despite these uncertainties, three time intervals: Berriasian-Hauterivian, Cenomanian and Santonian-Campanian can be recognized as periods of diversification for selachians, including the apparition of numerous modern selachian clades (many lamniform, squaliform and batoid families). It is noteworthy that the Cenomanian stage also corresponds to an important radiation event for ray-finned fishes [44], which shared similar marine environments with selachians. The Cretaceous/Paleocene boundary is often regarded as the first major extinction event in the selachian evolutionary history. During this extinction event eleven families and one order (Hybodontiformes) went extinct [28] (see also [41], [45]). Although not considering data on hybodont sharks (the authors argued that only a single Maastrichtian species was known), it has been suggested [28] that extinction levels were similar among ecological selachian groups, with the exception of benthopelagic and deep-sea taxa, which were less affected. Whatever the causes of the mass disappearance of selachian taxa at the K/T boundary, it is certain that this extinction event affected this group, but probably in a lower order of magnitude than expected when standing diversity is considered. However, our results suggest a Triassic/ Jurassic diversity drop of similar amplitude to the K/T extinction. The vast majority of families and genera concerned by this extinction are hybodont sharks or selachian of doubtful affinities (e.g. Pseudodalatiidae, Homalodontidae, *Hueneichthys*,) as neoselachian groups remain poorly represented. Although numerous works carried on the Triassic/ Jurassic boundary suggest an important extinction (end-Triassic extinction) for a number of terrestrial (e.g. Therapsida, early amphibians) and marine groups (e.g. most ammonoids, conodonts, most bivalves, xenacanthimorph sharks), no studies on selachians have reported an impact of the end-Triassic extinction on this group. Even if proposed reasons for this mass extinction are numerous [46], the eruption of the Central Atlantic magmatic province [47], [48] associated with the break up of the Pangea is likely. The combination of the extinction of a number of shark groups (particularly among hybodont sharks) and the apparition of new ecological niches probably favored the diversification of new shark groups fulfilling these free niches, as indicated by the subsequent major Hettangian diversification identified here (see above). However, no major biotic/ abiotic crises corresponding to the series of late Eocene – Oligocene selachian generic diversity drops following the slight early Paleogene recovery have been reported and no recent studies on Cenozoic selachian diversity have been carried out yet (but see [41]). Studies on Tertiary paleoclimates [49]–[50] indicate that the early Paleogene corresponds to a period of high atmospheric temperatures including the Paleocene/Eocene Thermal Maximum, the Early Eocene Climatic Optimum and the mid-Eocene Climatic Optimum, whereas temperatures drop dramatically in the Bartonian (along with the onset of Antarctic ice sheets) and stay low in the Oligocene. Thus, a positive correlation between temperatures and selachian diversity may explain the patterns observed for this time interval. Such a correlation has moreover been reported for numerous living marine organisms [51] and it is likely that this prevailed in the Cenozoic. Similarly, inferred generic diversity keeps decreasing afterward (familial and ordinal diversities stagnate) until Recent, as paleotemperatures do [50].

### Conclusion

This study presents an innovative methodology for combining phylogenetic hypotheses and stratigraphy using two branching methods (CBM and DDBM) with the purpose of inferring highest and lowest boundaries of the true selachian taxic diversity and evolutionary history. This has been applied on three taxonomic ranks (orders, families and genera) and seven phylogenetic hypotheses on a period encompassing 300 Myrs. For the first time, the selachian fossil record is quantitatively assessed, suggesting a globally poor record for lower taxonomic ranks (genera, families) when phylogenetic relationships are considered. We also present the first comprehensive analysis of major events that are likely to have marked the selachian evolutionary history. Some of them were mentioned in previous works using alternative methods (Early Jurassic, mid-Cretaceous, K/T boundary and late Paleogene diversity drops), thus reinforcing the efficiency of the methodology presented here in inferring such evolutionary events. Other events (Permian/Triassic, Early and Late Cretaceous diversifications; Triassic/ Jurassic extinction) are identified for the first time.

## Supporting Information

Figure S1 **Example of RCI scores obtained for an unresolved tree and for corresponding resolved trees with replications.**
(TIF)

Table S1 **RCI (using CBM) and RCI’ (using DDBM) scores for the various phylogenetic hypotheses considered.** ‘Unresolved’ corresponds to the best RCI and RCI’ scores considering trees with polytomies; ‘resolved’ indicates best RCI and RCI’ scores for trees with resolved polytomies and corresponding number of possible trees. Values in bold indicate best scores for each taxonomic rank.
(XLS)

Table S2 **Correlation table using the Spearman rank correlation coefficient and corresponding p-values.** Values in bold indicate statistically significant correlations. O = Order; F = Family; G = Genus.
(XLS)

File S1 **Phylogenetic hypotheses used in the analyses.**
(DOC)

File S2 **Stratigraphic framework used in the analyses.**
(DOC)

File S3 **Observed and computed stratigraphic ranges for genera, families and orders.**
(DOC)
