# Supplementary material for: An Analytical Approach for Estimating Fossil Record and Diversification Events in Sharks, Skates and Rays
Source: PLoS One. 2012 Sep 5;7(9):e44632. doi: 10.1371/journal.pone.0044632 (PMC3434181; doi:10.1371/journal.pone.0044632)
Supplement: File S1 — Phylogenetic hypotheses used in the analyses. (DOC) [file pone.0044632.s004.doc]

**Supporting Information File S1**

Phylogenetic hypotheses used in the analyses

Case of living taxa

For each selachian order, with the exception of some orders encompassing only few taxa not necessitating phylogenetic resolution (e.g. Squatiniformes, Heterodontiformes), the following intra-ordinal phylogenetic relationships were selected: Orectolobiformes [1], Lamniformes [2,3], Carcharhiniformes [4] (subsequently modified [5] with a peculiar resolution of *Halaelurus*-related taxa [6]), Hexanchiformes [7], Squaliformes [8,9], Rajiformes [4] (with modifications [10-12]), Myliobatiformes [13] and Torpediniformes [12]. Concepts of Pristiformes, Rhiniformes and Rhinobatiformes [14] were not considered here because of the inconsistency in their contents according to authors [12,15] and their lumping with the Rajiformes. When ambiguous and/or distinct from Rajiformes *sensu* [4] in the phylogenies considered, we preferred the use of unnamed groups (e.g. “Pristid”, “Rhinobatid-like”) instead, thus limiting the clade Rajiformes to its indisputable families (e.g. Rajidae, Arhynchobatidae, Anacanthobatidae).

The recently published species-level shark phylogeny [16] is not fully considered here because of the large number of discrepancies (most of the families and numerous genera and orders are considered paraphyletic) and due to some incongruities that are not further argued (e.g. Ginglymostomatidae nested within Carcharhiniformes).

Case of fossil taxa

The systematic position of numerous fossil taxa is problematic. For instance, in terms of dental characters, basal neoselachians can be identified mainly on the basis of the possession of a triple-layered enameloid [17,18], the intermediary layer being considered a synapomorphy of neoselachians. However, this character remains subject to discussion because batoids and several fossil taxa lack this layer. Therefore, contrary to [19], we excluded the Devonian McMurdodontidae (genus *McMurdodus*) and the Carboniferous Anachronistidae (genera *Cooleyella* and *Ginteria*) from the Neoselachii, pending further evidences concerning their enameloid histology [20,21]. Concerning shark taxa, we chose to include the Late Triassic genera *Grozonodon*, *Hueneichthys* and *Pseudocetorhinus* within the Selachimorpha (modern sharks) because their teeth display a triple-layered enameloid microstructure [18,22,23]. The genus *Reifia* is included within the galean selachimorphs according to both the triple-layered microstructure of its enameloid [24] and to the overall morphological aspect of its teeth [10,11,24]. The Rhaetian genus *Vallisia* is considered more closely-related to the hybodonts than to the neoselachian sharks because the two former share a single-layered enameloid; however, *Vallisia* is not included within the hybodonts due to differences in dental morphologies [23]. According to different studies, the genus *Parasquatina* is considered either as a possible squatiniform [25] or as an orectolobiform [10] and is therefore placed in stem position to these two orders in the phylogenetic hypotheses considered here. The family Echinorhinidae (sometimes considered in its own order Echinorhiniformes [21]), when not represented in the phylogenetic hypotheses selected, was placed in basal position to the clade Squalomorphii (Squaliformes, Squatiniformes, Pristiophoriformes and Hexanchiformes), as for the fossil family Protospinacidae. Regarding the batoids, the main issue concerns the interpretation of the order Rajiformes. According to [11], this order includes the Rhinobatoidei, Rajoidei, Sclerorhynchoidei and Pristioidei and other taxa of unknown affinities (e.g., *Ptychotrygon*, *Texatrygon*, *Safagaia*), whereas other classifications consider these suborders as proper orders or with ambiguous phylogenetic relationships [12]. Thus, when a phylogenetic hypothesis retains no close relationships between Rhinobatoidei, Pristioidei (referred to as “Rhinobatoid-like” and “Pristids” here, respectively) and Rajiformes *sensu lato*, other rajiform taxa (e.g., Sclerorhynchoidei) were left in polytomies at the base of the clade.

Phylogenetic hypotheses used including fossil taxa

All genus, family and order-level phylogenetic hypotheses are given in nexus format. The phylogenetic hypotheses of HEI[27] are also given in circular trees as an example.

*Genus-level phylogenies:*

**DOU[26]**

(((((((Aegyptobatus,Tribodus),Distobatus,Glickmanodus,Reticulodus,Acrodus,Acrorhizodus,Asteracanthus,Bdellodus),(Egertonodus,Pororhiza,Priohybodus,Thaiodus,Leptacanthus,Planohybodus,Secarodus,Hybodus,Meristodon,Meristodonoides,Khoratodus)),(Palaeobates,Polyacrodus)),(Lonchidion,Lissodus,Bahariyodon,Hylaeobatis,Isanodus,Parvodus,Vectiselachos,Jiaodontus,Diplolonchidion)),(Homalodontus,Doratodus),(Heteroptychodus,Ptychodus),Steinbachodus,Pseudodalatias,Ostenoselache,Hubeiodus,Mukdahanodus,Raineria),(((((((((Burnhamia,Eomobula,(Eoplinthicus,Plinthicus),Manta,Mobula),Rhinoptera),Ixobatis,(Pucabatis,Rhombodus,Washakiebatis),Aetobatus,Aetomylaeus,Apocopodon,Brachyrhizodus,Garabatis,Igdabatis,Leidybatis,Lophobatis,(Myliobatis,Pteromylaeus),Pseudaetobatus,Aktaua),(Pteroplatea,Aetoplatea,Gymnura,Jacquhermania,Ouledia),Cretomanta,Archaeomanta),((Urolophus,Trygonoptera,Plesiobatis),Himantura),((Potamotrygon,Plesiotrygon),(Paratrygon,Heliotrygon)),Arechia,Aturobatis,Coupatezia,Dasyrhombodus,Delpitia,Enantiobatis,Ewingia,Heterobatis,Heterotorpedo,Merabatis,Myliodasyatis,Phosphatodon,Meridiania,Texabatis,Viperecucullus,(Urotrygon,Urobatis),Hypolophites,Hypolophodon,Ishaquia,Pastinachus,Taeniura,Dasyatis,Neotrygon,Pteroplatytrygon,Taeniurops,Urogymnus,Asterotrygon,Heliobatis),Hexatrygon),Zanobatus),(((Torpedo,Eotorpedo),Hypnos),((Heteronarce,Typhlonarke,Narke,Temera,Crassinarke,Electrolux),(Narcine,Benthobatis,Diplobatis,Discopyge),Titanonarke)),(Ataktobatis,Engaibatis,Jurobatos,Microbatis,Myledaphus,Protoplatyrhina,Pseudohypolophus,Squatirhina,Vascobatis,(Rhina,Rhynchobatus,((Asterodermus,Belemnobatis),Doliobatis,Iansan,Isidobatus,Paratrygonorrhina,Rhinobatos,Rhombopterygia,Spathobatis,Aptychotrema,Trygonorrhina,Zapteryx,Platypterix),(Platyrhina,Tethybatis,Platyrhinoidis),(Hypsobatis,Angolabatis,Youssoubatis),Parapalaeobates)),(((Dipturus,Raja,Malacoraja,Mafdetia,Rajorhina,Smithraja,Walteraja,Amblyraja,Breviraja,Dactylobatus,Dentiraja,Fenestraja,Gurgesiella,Hongeo,Insentiraja,Leucoraja,Neoraja,Okamejei,Rajella,Rostroraja,Zearaja),(Anacanthobatis,Sinobatis),(Psammobatis,Pseudoraja,Rioraja,Rhinoraja,Pavoraja,Notoraja,Irolita,Brochiraja,Bathyraja,Atlantoraja,Arhynchobatis,Sympterygia)),Cyclobatis),(Anoxypristis,Pristis,Propristis),(Cristabatis,Toarcibatis),((Onchosaurus,Pucapristis,Schizorhiza),(Ankistrorhynchus,Borodinopristis,(Columbusia,Onchopristis),Ctenopristis,Dalpiazia,Ganopristis,Ischyrhiza,Libanopristis,Marckgrafia,Micropristis,Plicatopristis,Sclerorhynchus,Kiestus,Atlanticopristis),Biropristis,Baharipristis,Renpetia,Celtipristis),Ptychotrygon,Ptychotrygonoides,Texatrygon,Archingeayia,Duwibatis,Engolismaia,Erguitaia,Erythrobatis,Hamrabatis,Safagaia,Tanoutia,Turoniabatis,Leiribatos,Phosphatobatis,Iberotrygon),((((((((((((((Etmopterus,Miroscyllium),Eoetmopterus,Microetmopterus,Paraetmopterus,Proetmopterus),(Centroscyllium,Aculeola),Trigonognathus),((Centroselachus,Proscymnodon,(Scymnodon,Zameus),Scymnodalatias,Cretascymnus,Centroscymnus),(SomniosusS,SomniosusR)),(Oxynotus,Protoxynotus),((Eosqualiolus,Squaliolus,Euprotomicrus),Heteroscymnoides,Paraphorosoides,Acrosqualiolus,Angoumeius,Squaliodalatias,Dalatias,Isistius,Euprotomicroides,Mollisquama)),(Protocentrophorus,(Centrophorus,Deania))),((Squalus,Cirrhigaleus,Protosqualus,Centrophoroides),Centrosqualus,Megasqualus)),Protospinax),((Squatina,Pseudorhina),(Pristiophorus,(Ikamauius,Pliotrema)))),(((Hexanchus,(Heptranchias,Paraheptranchias),Weltonia),Notorynchus,Notidanoides,Notidanodon,Pachyhexanchus,(Pseudonotidanus,Welcommia)),(Chlamydoselachus,Eothrinax),Sphenodus,Paraorthacodus),(Echinorhinus,Gibbechinorhinus,Orthechinorhinus,Paraechinorhinus,Pseudoechinorhinus)),((Dwardius,Priscusurus,Eoptolamna,(Mitsukurina,Scapanorhynchus,Anomotodon,Striatolamia,Woellsteinia),((Acrolamna,Archaeolamna,Cretodus,Cretoxyrhina,Dallasiela,Trigonotodus,Eostriatolamia,Paraisurus,Palaeocarcharodon,Cretolamna,Telodontaspis),Protolamna,Leptostyrax,Pseudoscapanorhynchus),((((Carcharias,Hispidaspis),Araloselachus),Brachycarcharias,Cenocarcharias,Glueckmanotodus,Hypotodus,Jaekelotodus,Johnlongia,(Mennerotodus,Borealotodus),Odontaspis,Orpodon,Palaeohypotodus,Roulletia,Sylvestrilamia,Turania,Pueblocarcharias),((Alopias,Anotodus,Paranomotodon,Usakias),Pseudocarcharias,Megachasma),((OtodusO,OtodusC,OtodusM),Parotodus),Cardabiodon,Serratolamna,((Galeocorax,Pseudocorax),(Ptychocorax,Nanocorax,Squalicorax,Scindocorax)),(Cetorhinus,(((Carchariolamna,Lamna,Carcharoides),Isurolamna,(Isurus,Macrorhizodus)),(Carcharodon,Cosmopolitodus),Karaisurus,Lethenia,Xiphodolamia)))),((Bavariscyllium,Casieria,Cretascyliorhinus,Eypea,(Foumtizia,Pachyscyllium),Macrourogaleus,Megascyliorhinus,Microscyliorhinus,Crassescyliorhinus,Palaeoscyllium,Platyrhizoscyllium,(PremontreiaP,PremontreiaO),Protoscyliorhinus,Pseudoscyliorhinus,Pteroscyllium,Stenoscyllium,Schroederichthys,(((Porodermoides,Poroderma),(Scyliorhinus,Cephaloscyllium)),(Atelomycterus,Aulohalaelurus)),(Galeus,Bythaelurus,(Asymbolus,(Cephalurus,Parmaturus)),(Holohalaelurus,Halaelurus,Haploblepharus,Prohaploblepharus),(Pentanchus,Apristurus)),(Proscyllium,Praeproscyllium,Ctenacis,Eridacnis),((Pseudotriakis,Gollum),(Leptocharias,(Archaeotriakis,(Iago,Khouribgaleus,Gomphogaleus,Galeorhinus,(Triakis,Scylliogaleus,Mustelus,Hemitriakis,Furgaleus,Pachygaleus,Palaeogaleus,Paratriakis,Squatigaleus,Xystrogaleus,((Hemigaleus,Paragaleus,(Chaenogaleus,Hemipristis),Moerigaleus),((Sphyrna,Eusphyra),((Abdounia,Tingaleus),Danogaleus,Eogaleus,Physogaleus,(Rhizoprionodon,Scoliodon,Loxodon,Isogomphodon,(Triaenodon,((Glyphis,Lamiopsis),Prionace,Nasolamia,Carcharhinus))),Kruckowlamna,Misrichthys,Negaprion,Galeocerdo)))))))),Figaro,Gogolia),Corysodon))),(Palaeorectolobus,Agaleus,Palaeocarcharias,Pseudospinax,(Annea,Dorsetoscyllium,Folipistrix,(Phorcynis,Heterophorcynus),Ornatoscyllium,(Pararhincodon,Cirrhoscyllium,Parascyllium),((((Ginglymostoma,Plicatoscyllium),Cantioscyllium,Protoginglymostoma,(Palaeorhincodon,Rhincodon),Stegostoma,Pseudoginglymostoma,Nebrius,Delpitoscyllium,Ganntouria,Hologinglymostoma),(Acanthoscyllium,Almascyllium,Hemiscyllium,Chiloscyllium,Mesiteia)),(Palaeobrachaelurus,Cretorectolobus,(Paraginglymostoma,Brachaelurus,Eostegostoma,Heteroscyllium),(Cederstroemia,Orectoloboides,(Eometlaouia,Squatiscyllium),Orectolobus,Eucrossorhinus,Sutorectus))))),Parasquatina),(Heterodontus,Paracestracion,Proheterodontus)),(Reifia,(Breviacanthus,(Mucrovenator,Rhomphaiodon),Palidiplospinax,Synechodus))),Pseudocetorhinus,Hueneichthys,Grozonodon)),Vallisia)

**HEI[27]**

(((((((Aegyptobatus,Tribodus),Distobatus,Glickmanodus,Reticulodus,Acrodus,Acrorhizodus,Asteracanthus,Bdellodus),(Egertonodus,Pororhiza,Priohybodus,Thaiodus,Leptacanthus,Planohybodus,Secarodus,Hybodus,Meristodon,Meristodonoides,Khoratodus)),(Palaeobates,Polyacrodus)),(Lonchidion,Lissodus,Bahariyodon,Hylaeobatis,Isanodus,Parvodus,Vectiselachos,Jiaodontus,Diplolonchidion)),(Homalodontus,Doratodus),(Heteroptychodus,Ptychodus),Steinbachodus,Pseudodalatias,Ostenoselache,Hubeiodus,Mukdahanodus,Raineria),(((((((((((Burnhamia,Eomobula,(Eoplinthicus,Plinthicus),Manta,Mobula),Rhinoptera),Ixobatis,(Pucabatis,Rhombodus,Washakiebatis),Aetobatus,Aetomylaeus,Apocopodon,Brachyrhizodus,Garabatis,Igdabatis,Leidybatis,Lophobatis,(Myliobatis,Pteromylaeus),Pseudaetobatus,Aktaua),(Pteroplatea,Aetoplatea,Gymnura,Jacquhermania,Ouledia),Cretomanta,Archaeomanta),((Urolophus,Trygonoptera,Plesiobatis),Himantura),((Potamotrygon,Plesiotrygon),(Paratrygon,Heliotrygon)),Arechia,Aturobatis,Coupatezia,Dasyrhombodus,Delpitia,Enantiobatis,Ewingia,Heterobatis,Heterotorpedo,Merabatis,Myliodasyatis,Phosphatodon,Meridiania,Texabatis,Viperecucullus,(Urotrygon,Urobatis),Hypolophites,Hypolophodon,Ishaquia,Pastinachus,Taeniura,Dasyatis,Neotrygon,Pteroplatytrygon,Taeniurops,Urogymnus,Asterotrygon,Heliobatis),Hexatrygon),Zanobatus),(Ataktobatis,Engaibatis,Jurobatos,Microbatis,Myledaphus,Protoplatyrhina,Pseudohypolophus,Squatirhina,Vascobatis,(Rhina,Rhynchobatus,((Asterodermus,Belemnobatis),Doliobatis,Iansan,Isidobatus,Paratrygonorrhina,Rhinobatos,Rhombopterygia,Spathobatis,Aptychotrema,Trygonorrhina,Zapteryx,Platypterix),(Platyrhina,Tethybatis,Platyrhinoidis),(Hypsobatis,Angolabatis,Youssoubatis),Parapalaeobates))),(((Torpedo,Eotorpedo),Hypnos),((Heteronarce,Typhlonarke,Narke,Temera,Crassinarke,Electrolux),(Narcine,Benthobatis,Diplobatis,Discopyge),Titanonarke))),(((Dipturus,Raja,Malacoraja,Mafdetia,Rajorhina,Smithraja,Walteraja,Amblyraja,Breviraja,Dactylobatus,Dentiraja,Fenestraja,Gurgesiella,Hongeo,Insentiraja,Leucoraja,Neoraja,Okamejei,Rajella,Rostroraja,Zearaja),(Anacanthobatis,Sinobatis),(Psammobatis,Pseudoraja,Rioraja,Rhinoraja,Pavoraja,Notoraja,Irolita,Brochiraja,Bathyraja,Atlantoraja,Arhynchobatis,Sympterygia)),Cyclobatis),(Anoxypristis,Pristis,Propristis),(Cristabatis,Toarcibatis),((Onchosaurus,Pucapristis,Schizorhiza),(Ankistrorhynchus,Borodinopristis,(Columbusia,Onchopristis),Ctenopristis,Dalpiazia,Ganopristis,Ischyrhiza,Libanopristis,Marckgrafia,Micropristis,Plicatopristis,Sclerorhynchus,Kiestus,Atlanticopristis),Biropristis,Baharipristis,Renpetia,Celtipristis),Ptychotrygon,Ptychotrygonoides,Texatrygon,Archingeayia,Duwibatis,Engolismaia,Erguitaia,Erythrobatis,Hamrabatis,Safagaia,Tanoutia,Turoniabatis,Leiribatos,Phosphatobatis,Iberotrygon),(Pseudocetorhinus,Hueneichthys,Grozonodon,(((((((((((Etmopterus,Miroscyllium),Eoetmopterus,Microetmopterus,Paraetmopterus,Proetmopterus),(Centroscyllium,Aculeola),Trigonognathus),((Centroselachus,Proscymnodon,(Scymnodon,Zameus),Scymnodalatias,Cretascymnus,Centroscymnus),(SomniosusS,SomniosusR)),(Oxynotus,Protoxynotus),((Eosqualiolus,Squaliolus,Euprotomicrus),Heteroscymnoides,Paraphorosoides,Acrosqualiolus,Angoumeius,Squaliodalatias,Dalatias,Isistius,Euprotomicroides,Mollisquama)),(Protocentrophorus,(Centrophorus,Deania))),((Squalus,Cirrhigaleus,Protosqualus,Centrophoroides),Centrosqualus,Megasqualus)),(Squatina,Pseudorhina)),(Pristiophorus,(Ikamauius,Pliotrema))),(((Hexanchus,(Heptranchias,Paraheptranchias),Weltonia),Notorynchus,Notidanoides,Notidanodon,Pachyhexanchus,(Pseudonotidanus,Welcommia)),(Chlamydoselachus,Eothrinax),Sphenodus,Paraorthacodus)),(Echinorhinus,Gibbechinorhinus,Orthechinorhinus,Paraechinorhinus,Pseudoechinorhinus),Protospinax),(Reifia,(Breviacanthus,(Mucrovenator,Rhomphaiodon),Palidiplospinax,Synechodus),((Heterodontus,Paracestracion,Proheterodontus),((Palaeorectolobus,Agaleus,Palaeocarcharias,Pseudospinax,(Annea,Dorsetoscyllium,Folipistrix,(Phorcynis,Heterophorcynus),Ornatoscyllium,(Pararhincodon,Cirrhoscyllium,Parascyllium),((((Ginglymostoma,Plicatoscyllium),Cantioscyllium,Protoginglymostoma,(Palaeorhincodon,Rhincodon),Stegostoma,Pseudoginglymostoma,Nebrius,Delpitoscyllium,Ganntouria,Hologinglymostoma),(Acanthoscyllium,Almascyllium,Hemiscyllium,Chiloscyllium,Mesiteia)),(Palaeobrachaelurus,Cretorectolobus,(Paraginglymostoma,Brachaelurus,Eostegostoma,Heteroscyllium),(Cederstroemia,Orectoloboides,(Eometlaouia,Squatiscyllium),Orectolobus,Eucrossorhinus,Sutorectus))))),((Dwardius,Priscusurus,Eoptolamna,(Mitsukurina,Scapanorhynchus,Anomotodon,Striatolamia,Woellsteinia),((Acrolamna,Archaeolamna,Cretodus,Cretoxyrhina,Dallasiela,Trigonotodus,Eostriatolamia,Paraisurus,Palaeocarcharodon,Cretolamna,Telodontaspis),Protolamna,Leptostyrax,Pseudoscapanorhynchus),((((Carcharias,Hispidaspis),Araloselachus),Brachycarcharias,Cenocarcharias,Glueckmanotodus,Hypotodus,Jaekelotodus,Johnlongia,(Mennerotodus,Borealotodus),Odontaspis,Orpodon,Palaeohypotodus,Roulletia,Sylvestrilamia,Turania,Pueblocarcharias),((Alopias,Anotodus,Paranomotodon,Usakias),Pseudocarcharias,Megachasma),((OtodusO,OtodusC,OtodusM),Parotodus),Cardabiodon,Serratolamna,((Galeocorax,Pseudocorax),(Ptychocorax,Nanocorax,Squalicorax,Scindocorax)),(Cetorhinus,(((Carchariolamna,Lamna,Carcharoides),Isurolamna,(Isurus,Macrorhizodus)),(Carcharodon,Cosmopolitodus),Karaisurus,Lethenia,Xiphodolamia)))),((Bavariscyllium,Casieria,Cretascyliorhinus,Eypea,(Foumtizia,Pachyscyllium),Macrourogaleus,Megascyliorhinus,Microscyliorhinus,Crassescyliorhinus,Palaeoscyllium,Platyrhizoscyllium,(PremontreiaP,PremontreiaO),Protoscyliorhinus,Pseudoscyliorhinus,Pteroscyllium,Stenoscyllium,Schroederichthys,(((Porodermoides,Poroderma),(Scyliorhinus,Cephaloscyllium)),(Atelomycterus,Aulohalaelurus)),(Galeus,Bythaelurus,(Asymbolus,(Cephalurus,Parmaturus)),(Holohalaelurus,Halaelurus,Haploblepharus,Prohaploblepharus),(Pentanchus,Apristurus)),(Proscyllium,Praeproscyllium,Ctenacis,Eridacnis),((Pseudotriakis,Gollum),(Leptocharias,(Archaeotriakis,(Iago,Khouribgaleus,Gomphogaleus,Galeorhinus,(Triakis,Scylliogaleus,Mustelus,Hemitriakis,Furgaleus,Pachygaleus,Palaeogaleus,Paratriakis,Squatigaleus,Xystrogaleus,((Hemigaleus,Paragaleus,(Chaenogaleus,Hemipristis),Moerigaleus),((Sphyrna,Eusphyra),((Abdounia,Tingaleus),Danogaleus,Eogaleus,Physogaleus,(Rhizoprionodon,Scoliodon,Loxodon,Isogomphodon,(Triaenodon,((Glyphis,Lamiopsis),Prionace,Nasolamia,Carcharhinus))),Kruckowlamna,Misrichthys,Negaprion,Galeocerdo)))))))),Figaro,Gogolia),Corysodon))))),Parasquatina))),Vallisia)


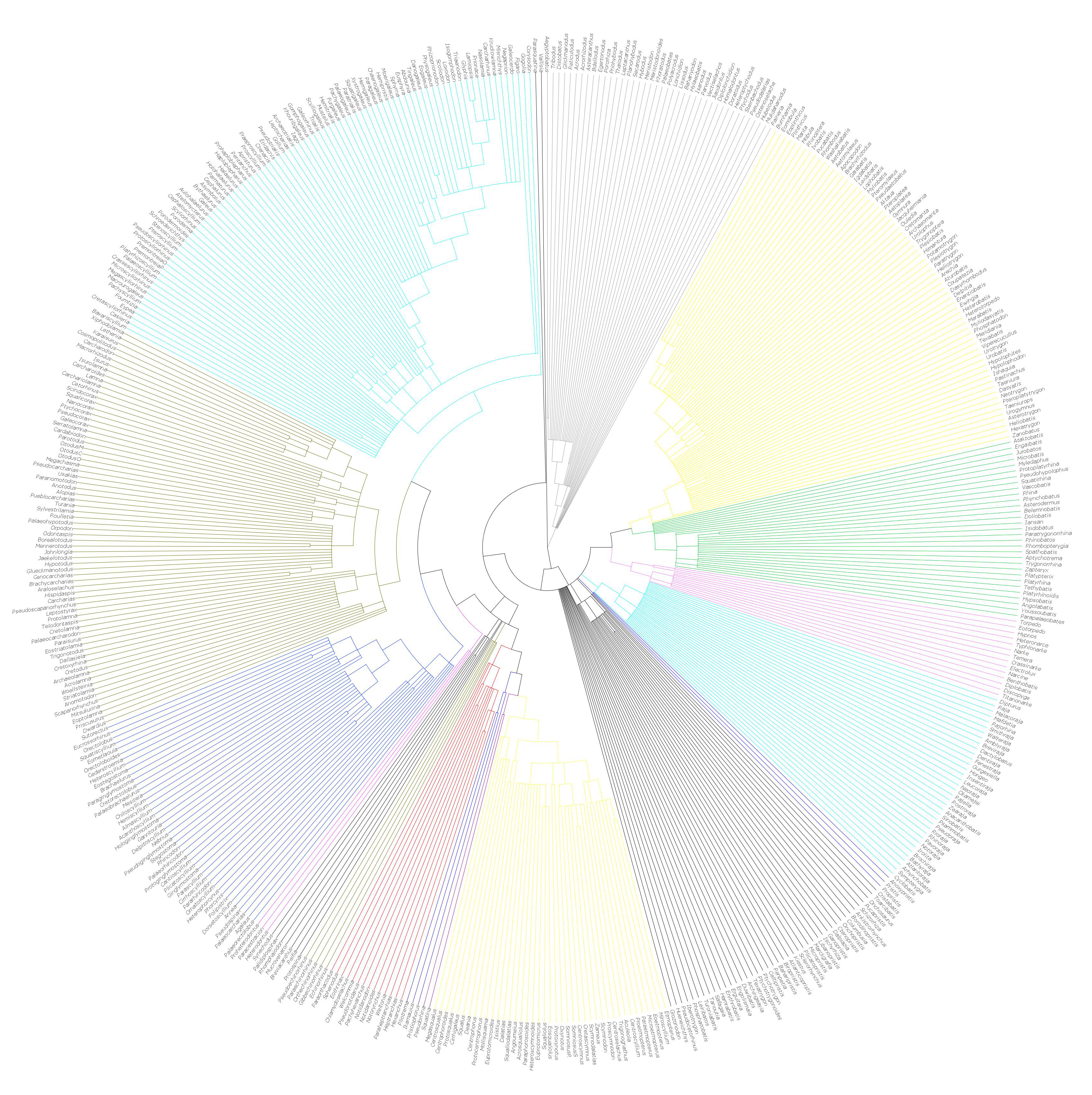


**HUM[28]**

(((((((Aegyptobatus,Tribodus),Distobatus,Glickmanodus,Reticulodus,Acrodus,Acrorhizodus,Asteracanthus,Bdellodus),(Egertonodus,Pororhiza,Priohybodus,Thaiodus,Leptacanthus,Planohybodus,Secarodus,Hybodus,Meristodon,Meristodonoides,Khoratodus)),(Palaeobates,Polyacrodus)),(Lonchidion,Lissodus,Bahariyodon,Hylaeobatis,Isanodus,Parvodus,Vectiselachos,Jiaodontus,Diplolonchidion)),(Homalodontus,Doratodus),(Heteroptychodus,Ptychodus),Steinbachodus,Pseudodalatias,Ostenoselache,Hubeiodus,Mukdahanodus,Raineria),((((((Dipturus,Raja,Malacoraja,Mafdetia,Rajorhina,Smithraja,Walteraja,Amblyraja,Breviraja,Dactylobatus,Dentiraja,Fenestraja,Gurgesiella,Hongeo,Insentiraja,Leucoraja,Neoraja,Okamejei,Rajella,Rostroraja,Zearaja),(Anacanthobatis,Sinobatis),(Psammobatis,Pseudoraja,Rioraja,Rhinoraja,Pavoraja,Notoraja,Irolita,Brochiraja,Bathyraja,Atlantoraja,Arhynchobatis,Sympterygia)),Cyclobatis),(Anoxypristis,Pristis,Propristis),(Cristabatis,Toarcibatis),((Onchosaurus,Pucapristis,Schizorhiza),(Ankistrorhynchus,Borodinopristis,(Columbusia,Onchopristis),Ctenopristis,Dalpiazia,Ganopristis,Ischyrhiza,Libanopristis,Marckgrafia,Micropristis,Plicatopristis,Sclerorhynchus,Kiestus,Atlanticopristis),Biropristis,Baharipristis,Renpetia,Celtipristis),Ptychotrygon,Ptychotrygonoides,Texatrygon,Archingeayia,Duwibatis,Engolismaia,Erguitaia,Erythrobatis,Hamrabatis,Safagaia,Tanoutia,Turoniabatis,Leiribatos,Phosphatobatis,Iberotrygon),((((((((Burnhamia,Eomobula,(Eoplinthicus,Plinthicus),Manta,Mobula),Rhinoptera),Ixobatis,(Pucabatis,Rhombodus,Washakiebatis),Aetobatus,Aetomylaeus,Apocopodon,Brachyrhizodus,Garabatis,Igdabatis,Leidybatis,Lophobatis,(Myliobatis,Pteromylaeus),Pseudaetobatus,Aktaua),(Pteroplatea,Aetoplatea,Gymnura,Jacquhermania,Ouledia),Cretomanta,Archaeomanta),((Urolophus,Trygonoptera,Plesiobatis),Himantura),((Potamotrygon,Plesiotrygon),(Paratrygon,Heliotrygon)),Arechia,Aturobatis,Coupatezia,Dasyrhombodus,Delpitia,Enantiobatis,Ewingia,Heterobatis,Heterotorpedo,Merabatis,Myliodasyatis,Phosphatodon,Meridiania,Texabatis,Viperecucullus,(Urotrygon,Urobatis),Hypolophites,Hypolophodon,Ishaquia,Pastinachus,Taeniura,Dasyatis,Neotrygon,Pteroplatytrygon,Taeniurops,Urogymnus,Asterotrygon,Heliobatis),Hexatrygon),Zanobatus),(Ataktobatis,Engaibatis,Jurobatos,Microbatis,Myledaphus,Protoplatyrhina,Pseudohypolophus,Squatirhina,Vascobatis,(Rhina,Rhynchobatus,((Asterodermus,Belemnobatis),Doliobatis,Iansan,Isidobatus,Paratrygonorrhina,Rhinobatos,Rhombopterygia,Spathobatis,Aptychotrema,Trygonorrhina,Zapteryx,Platypterix),(Platyrhina,Tethybatis,Platyrhinoidis),(Hypsobatis,Angolabatis,Youssoubatis),Parapalaeobates))),(((Torpedo,Eotorpedo),Hypnos),((Heteronarce,Typhlonarke,Narke,Temera,Crassinarke,Electrolux),(Narcine,Benthobatis,Diplobatis,Discopyge),Titanonarke))),((((((((((((Etmopterus,Miroscyllium),Eoetmopterus,Microetmopterus,Paraetmopterus,Proetmopterus),(Centroscyllium,Aculeola),Trigonognathus),((Centroselachus,Proscymnodon,(Scymnodon,Zameus),Scymnodalatias,Cretascymnus,Centroscymnus),(SomniosusS,SomniosusR)),(Oxynotus,Protoxynotus),((Eosqualiolus,Squaliolus,Euprotomicrus),Heteroscymnoides,Paraphorosoides,Acrosqualiolus,Angoumeius,Squaliodalatias,Dalatias,Isistius,Euprotomicroides,Mollisquama)),(Protocentrophorus,(Centrophorus,Deania))),((Squalus,Cirrhigaleus,Protosqualus,Centrophoroides),Centrosqualus,Megasqualus)),((Pristiophorus,(Ikamauius,Pliotrema)),(Squatina,Pseudorhina))),(Heterodontus,Paracestracion,Proheterodontus)),(Palaeorectolobus,Agaleus,Palaeocarcharias,Pseudospinax,(Annea,Dorsetoscyllium,Folipistrix,(Phorcynis,Heterophorcynus),Ornatoscyllium,(Pararhincodon,Cirrhoscyllium,Parascyllium),((((Ginglymostoma,Plicatoscyllium),Cantioscyllium,Protoginglymostoma,(Palaeorhincodon,Rhincodon),Stegostoma,Pseudoginglymostoma,Nebrius,Delpitoscyllium,Ganntouria,Hologinglymostoma),(Acanthoscyllium,Almascyllium,Hemiscyllium,Chiloscyllium,Mesiteia)),(Palaeobrachaelurus,Cretorectolobus,(Paraginglymostoma,Brachaelurus,Eostegostoma,Heteroscyllium),(Cederstroemia,Orectoloboides,(Eometlaouia,Squatiscyllium),Orectolobus,Eucrossorhinus,Sutorectus))))),((Dwardius,Priscusurus,Eoptolamna,(Mitsukurina,Scapanorhynchus,Anomotodon,Striatolamia,Woellsteinia),((Acrolamna,Archaeolamna,Cretodus,Cretoxyrhina,Dallasiela,Trigonotodus,Eostriatolamia,Paraisurus,Palaeocarcharodon,Cretolamna,Telodontaspis),Protolamna,Leptostyrax,Pseudoscapanorhynchus),((((Carcharias,Hispidaspis),Araloselachus),Brachycarcharias,Cenocarcharias,Glueckmanotodus,Hypotodus,Jaekelotodus,Johnlongia,(Mennerotodus,Borealotodus),Odontaspis,Orpodon,Palaeohypotodus,Roulletia,Sylvestrilamia,Turania,Pueblocarcharias),((Alopias,Anotodus,Paranomotodon,Usakias),Pseudocarcharias,Megachasma),((OtodusO,OtodusC,OtodusM),Parotodus),Cardabiodon,Serratolamna,((Galeocorax,Pseudocorax),(Ptychocorax,Nanocorax,Squalicorax,Scindocorax)),(Cetorhinus,(((Carchariolamna,Lamna,Carcharoides),Isurolamna,(Isurus,Macrorhizodus)),(Carcharodon,Cosmopolitodus),Karaisurus,Lethenia,Xiphodolamia)))),((Bavariscyllium,Casieria,Cretascyliorhinus,Eypea,(Foumtizia,Pachyscyllium),Macrourogaleus,Megascyliorhinus,Microscyliorhinus,Crassescyliorhinus,Palaeoscyllium,Platyrhizoscyllium,(PremontreiaP,PremontreiaO),Protoscyliorhinus,Pseudoscyliorhinus,Pteroscyllium,Stenoscyllium,Schroederichthys,(((Porodermoides,Poroderma),(Scyliorhinus,Cephaloscyllium)),(Atelomycterus,Aulohalaelurus)),(Galeus,Bythaelurus,(Asymbolus,(Cephalurus,Parmaturus)),(Holohalaelurus,Halaelurus,Haploblepharus,Prohaploblepharus),(Pentanchus,Apristurus)),(Proscyllium,Praeproscyllium,Ctenacis,Eridacnis),((Pseudotriakis,Gollum),(Leptocharias,(Archaeotriakis,(Iago,Khouribgaleus,Gomphogaleus,Galeorhinus,(Triakis,Scylliogaleus,Mustelus,Hemitriakis,Furgaleus,Pachygaleus,Palaeogaleus,Paratriakis,Squatigaleus,Xystrogaleus,((Hemigaleus,Paragaleus,(Chaenogaleus,Hemipristis),Moerigaleus),((Sphyrna,Eusphyra),((Abdounia,Tingaleus),Danogaleus,Eogaleus,Physogaleus,(Rhizoprionodon,Scoliodon,Loxodon,Isogomphodon,(Triaenodon,((Glyphis,Lamiopsis),Prionace,Nasolamia,Carcharhinus))),Kruckowlamna,Misrichthys,Negaprion,Galeocerdo)))))))),Figaro,Gogolia),Corysodon)),Parasquatina),(((Hexanchus,(Heptranchias,Paraheptranchias),Weltonia),Notorynchus,Notidanoides,Notidanodon,Pachyhexanchus,(Pseudonotidanus,Welcommia)),(Chlamydoselachus,Eothrinax),Sphenodus,Paraorthacodus),(Echinorhinus,Gibbechinorhinus,Orthechinorhinus,Paraechinorhinus,Pseudoechinorhinus),Protospinax),(Reifia,(Breviacanthus,(Mucrovenator,Rhomphaiodon),Palidiplospinax,Synechodus))),Pseudocetorhinus,Hueneichthys,Grozonodon)),Vallisia);

**MAI[29]**

(((((((Aegyptobatus,Tribodus),Distobatus,Glickmanodus,Reticulodus,Acrodus,Acrorhizodus,Asteracanthus,Bdellodus),(Egertonodus,Pororhiza,Priohybodus,Thaiodus,Leptacanthus,Planohybodus,Secarodus,Hybodus,Meristodon,Meristodonoides,Khoratodus)),(Palaeobates,Polyacrodus)),(Lonchidion,Lissodus,Bahariyodon,Hylaeobatis,Isanodus,Parvodus,Vectiselachos,Jiaodontus,Diplolonchidion)),(Homalodontus,Doratodus),(Heteroptychodus,Ptychodus),Steinbachodus,Pseudodalatias,Ostenoselache,Hubeiodus,Mukdahanodus,Raineria),((((((((((Burnhamia,Eomobula,(Eoplinthicus,Plinthicus),Manta,Mobula),Rhinoptera),Ixobatis,(Pucabatis,Rhombodus,Washakiebatis),Aetobatus,Aetomylaeus,Apocopodon,Brachyrhizodus,Garabatis,Igdabatis,Leidybatis,Lophobatis,(Myliobatis,Pteromylaeus),Pseudaetobatus,Aktaua),(Pteroplatea,Aetoplatea,Gymnura,Jacquhermania,Ouledia),Cretomanta,Archaeomanta),((Urolophus,Trygonoptera,Plesiobatis),Himantura),((Potamotrygon,Plesiotrygon),(Paratrygon,Heliotrygon)),Arechia,Aturobatis,Coupatezia,Dasyrhombodus,Delpitia,Enantiobatis,Ewingia,Heterobatis,Heterotorpedo,Merabatis,Myliodasyatis,Phosphatodon,Meridiania,Texabatis,Viperecucullus,(Urotrygon,Urobatis),Hypolophites,Hypolophodon,Ishaquia,Pastinachus,Taeniura,Dasyatis,Neotrygon,Pteroplatytrygon,Taeniurops,Urogymnus,Asterotrygon,Heliobatis),Hexatrygon),Zanobatus),(Anoxypristis,Pristis,Propristis)),(((Torpedo,Eotorpedo),Hypnos),((Heteronarce,Typhlonarke,Narke,Temera,Crassinarke,Electrolux),(Narcine,Benthobatis,Diplobatis,Discopyge),Titanonarke)),((Ataktobatis,Engaibatis,Jurobatos,Microbatis,Myledaphus,Protoplatyrhina,Pseudohypolophus,Squatirhina,Vascobatis,(Rhina,Rhynchobatus,((Asterodermus,Belemnobatis),Doliobatis,Iansan,Isidobatus,Paratrygonorrhina,Rhinobatos,Rhombopterygia,Spathobatis,Aptychotrema,Trygonorrhina,Zapteryx,Platypterix),(Platyrhina,Tethybatis,Platyrhinoidis),(Hypsobatis,Angolabatis,Youssoubatis),Parapalaeobates)),(((Dipturus,Raja,Malacoraja,Mafdetia,Rajorhina,Smithraja,Walteraja,Amblyraja,Breviraja,Dactylobatus,Dentiraja,Fenestraja,Gurgesiella,Hongeo,Insentiraja,Leucoraja,Neoraja,Okamejei,Rajella,Rostroraja,Zearaja),(Anacanthobatis,Sinobatis),(Psammobatis,Pseudoraja,Rioraja,Rhinoraja,Pavoraja,Notoraja,Irolita,Brochiraja,Bathyraja,Atlantoraja,Arhynchobatis,Sympterygia)),Cyclobatis)),(Cristabatis,Toarcibatis),((Onchosaurus,Pucapristis,Schizorhiza),(Ankistrorhynchus,Borodinopristis,(Columbusia,Onchopristis),Ctenopristis,Dalpiazia,Ganopristis,Ischyrhiza,Libanopristis,Marckgrafia,Micropristis,Plicatopristis,Sclerorhynchus,Kiestus,Atlanticopristis),Biropristis,Baharipristis,Renpetia,Celtipristis),Ptychotrygon,Ptychotrygonoides,Texatrygon,Archingeayia,Duwibatis,Engolismaia,Erguitaia,Erythrobatis,Hamrabatis,Safagaia,Tanoutia,Turoniabatis,Leiribatos,Phosphatobatis,Iberotrygon),(Pseudocetorhinus,Hueneichthys,Grozonodon,((((((((((Etmopterus,Miroscyllium),Eoetmopterus,Microetmopterus,Paraetmopterus,Proetmopterus),(Centroscyllium,Aculeola),Trigonognathus),((Centroselachus,Proscymnodon,(Scymnodon,Zameus),Scymnodalatias,Cretascymnus,Centroscymnus),(SomniosusS,SomniosusR)),(Oxynotus,Protoxynotus),((Eosqualiolus,Squaliolus,Euprotomicrus),Heteroscymnoides,Paraphorosoides,Acrosqualiolus,Angoumeius,Squaliodalatias,Dalatias,Isistius,Euprotomicroides,Mollisquama)),(Protocentrophorus,(Centrophorus,Deania))),((Squalus,Cirrhigaleus,Protosqualus,Centrophoroides),Centrosqualus,Megasqualus)),((Squatina,Pseudorhina),(Echinorhinus,Gibbechinorhinus,Orthechinorhinus,Paraechinorhinus,Pseudoechinorhinus))),(Pristiophorus,(Ikamauius,Pliotrema))),(((Hexanchus,(Heptranchias,Paraheptranchias),Weltonia),Notorynchus,Notidanoides,Notidanodon,Pachyhexanchus,(Pseudonotidanus,Welcommia)),(Chlamydoselachus,Eothrinax),Sphenodus,Paraorthacodus),Protospinax),(Reifia,(Breviacanthus,(Mucrovenator,Rhomphaiodon),Palidiplospinax,Synechodus),((Heterodontus,Paracestracion,Proheterodontus),((Palaeorectolobus,Agaleus,Palaeocarcharias,Pseudospinax,(Annea,Dorsetoscyllium,Folipistrix,(Phorcynis,Heterophorcynus),Ornatoscyllium,(Pararhincodon,Cirrhoscyllium,Parascyllium),((((Ginglymostoma,Plicatoscyllium),Cantioscyllium,Protoginglymostoma,(Palaeorhincodon,Rhincodon),Stegostoma,Pseudoginglymostoma,Nebrius,Delpitoscyllium,Ganntouria,Hologinglymostoma),(Acanthoscyllium,Almascyllium,Hemiscyllium,Chiloscyllium,Mesiteia)),(Palaeobrachaelurus,Cretorectolobus,(Paraginglymostoma,Brachaelurus,Eostegostoma,Heteroscyllium),(Cederstroemia,Orectoloboides,(Eometlaouia,Squatiscyllium),Orectolobus,Eucrossorhinus,Sutorectus))))),((Dwardius,Priscusurus,Eoptolamna,(Mitsukurina,Scapanorhynchus,Anomotodon,Striatolamia,Woellsteinia),((Acrolamna,Archaeolamna,Cretodus,Cretoxyrhina,Dallasiela,Trigonotodus,Eostriatolamia,Paraisurus,Palaeocarcharodon,Cretolamna,Telodontaspis),Protolamna,Leptostyrax,Pseudoscapanorhynchus),((((Carcharias,Hispidaspis),Araloselachus),Brachycarcharias,Cenocarcharias,Glueckmanotodus,Hypotodus,Jaekelotodus,Johnlongia,(Mennerotodus,Borealotodus),Odontaspis,Orpodon,Palaeohypotodus,Roulletia,Sylvestrilamia,Turania,Pueblocarcharias),((Alopias,Anotodus,Paranomotodon,Usakias),Pseudocarcharias,Megachasma),((OtodusO,OtodusC,OtodusM),Parotodus),Cardabiodon,Serratolamna,((Galeocorax,Pseudocorax),(Ptychocorax,Nanocorax,Squalicorax,Scindocorax)),(Cetorhinus,(((Carchariolamna,Lamna,Carcharoides),Isurolamna,(Isurus,Macrorhizodus)),(Carcharodon,Cosmopolitodus),Karaisurus,Lethenia,Xiphodolamia)))),((Bavariscyllium,Casieria,Cretascyliorhinus,Eypea,(Foumtizia,Pachyscyllium),Macrourogaleus,Megascyliorhinus,Microscyliorhinus,Crassescyliorhinus,Palaeoscyllium,Platyrhizoscyllium,(PremontreiaP,PremontreiaO),Protoscyliorhinus,Pseudoscyliorhinus,Pteroscyllium,Stenoscyllium,Schroederichthys,(((Porodermoides,Poroderma),(Scyliorhinus,Cephaloscyllium)),(Atelomycterus,Aulohalaelurus)),(Galeus,Bythaelurus,(Asymbolus,(Cephalurus,Parmaturus)),(Holohalaelurus,Halaelurus,Haploblepharus,Prohaploblepharus),(Pentanchus,Apristurus)),(Proscyllium,Praeproscyllium,Ctenacis,Eridacnis),((Pseudotriakis,Gollum),(Leptocharias,(Archaeotriakis,(Iago,Khouribgaleus,Gomphogaleus,Galeorhinus,(Triakis,Scylliogaleus,Mustelus,Hemitriakis,Furgaleus,Pachygaleus,Palaeogaleus,Paratriakis,Squatigaleus,Xystrogaleus,((Hemigaleus,Paragaleus,(Chaenogaleus,Hemipristis),Moerigaleus),((Sphyrna,Eusphyra),((Abdounia,Tingaleus),Danogaleus,Eogaleus,Physogaleus,(Rhizoprionodon,Scoliodon,Loxodon,Isogomphodon,(Triaenodon,((Glyphis,Lamiopsis),Prionace,Nasolamia,Carcharhinus))),Kruckowlamna,Misrichthys,Negaprion,Galeocerdo)))))))),Figaro,Gogolia),Corysodon))))),Parasquatina))),Vallisia)

**MAW[30]**

(((((((Aegyptobatus,Tribodus),Distobatus,Glickmanodus,Reticulodus,Acrodus,Acrorhizodus,Asteracanthus,Bdellodus),(Egertonodus,Pororhiza,Priohybodus,Thaiodus,Leptacanthus,Planohybodus,Secarodus,Hybodus,Meristodon,Meristodonoides,Khoratodus)),(Palaeobates,Polyacrodus)),(Lonchidion,Lissodus,Bahariyodon,Hylaeobatis,Isanodus,Parvodus,Vectiselachos,Jiaodontus,Diplolonchidion)),(Homalodontus,Doratodus),(Heteroptychodus,Ptychodus),Steinbachodus,Pseudodalatias,Ostenoselache,Hubeiodus,Mukdahanodus,Raineria),(((((((((((Burnhamia,Eomobula,(Eoplinthicus,Plinthicus),Manta,Mobula),Rhinoptera),Ixobatis,(Pucabatis,Rhombodus,Washakiebatis),Aetobatus,Aetomylaeus,Apocopodon,Brachyrhizodus,Garabatis,Igdabatis,Leidybatis,Lophobatis,(Myliobatis,Pteromylaeus),Pseudaetobatus,Aktaua),(Pteroplatea,Aetoplatea,Gymnura,Jacquhermania,Ouledia),Cretomanta,Archaeomanta),((Urolophus,Trygonoptera,Plesiobatis),Himantura),((Potamotrygon,Plesiotrygon),(Paratrygon,Heliotrygon)),Arechia,Aturobatis,Coupatezia,Dasyrhombodus,Delpitia,Enantiobatis,Ewingia,Heterobatis,Heterotorpedo,Merabatis,Myliodasyatis,Phosphatodon,Meridiania,Texabatis,Viperecucullus,(Urotrygon,Urobatis),Hypolophites,Hypolophodon,Ishaquia,Pastinachus,Taeniura,Dasyatis,Neotrygon,Pteroplatytrygon,Taeniurops,Urogymnus,Asterotrygon,Heliobatis),Hexatrygon),Zanobatus),(Ataktobatis,Engaibatis,Jurobatos,Microbatis,Myledaphus,Protoplatyrhina,Pseudohypolophus,Squatirhina,Vascobatis,(Rhina,Rhynchobatus,((Asterodermus,Belemnobatis),Doliobatis,Iansan,Isidobatus,Paratrygonorrhina,Rhinobatos,Rhombopterygia,Spathobatis,Aptychotrema,Trygonorrhina,Zapteryx,Platypterix),(Platyrhina,Tethybatis,Platyrhinoidis),(Hypsobatis,Angolabatis,Youssoubatis),Parapalaeobates))),(((Torpedo,Eotorpedo),Hypnos),((Heteronarce,Typhlonarke,Narke,Temera,Crassinarke,Electrolux),(Narcine,Benthobatis,Diplobatis,Discopyge),Titanonarke))),(((Dipturus,Raja,Malacoraja,Mafdetia,Rajorhina,Smithraja,Walteraja,Amblyraja,Breviraja,Dactylobatus,Dentiraja,Fenestraja,Gurgesiella,Hongeo,Insentiraja,Leucoraja,Neoraja,Okamejei,Rajella,Rostroraja,Zearaja),(Anacanthobatis,Sinobatis),(Psammobatis,Pseudoraja,Rioraja,Rhinoraja,Pavoraja,Notoraja,Irolita,Brochiraja,Bathyraja,Atlantoraja,Arhynchobatis,Sympterygia)),Cyclobatis),(Anoxypristis,Pristis,Propristis),(Cristabatis,Toarcibatis),((Onchosaurus,Pucapristis,Schizorhiza),(Ankistrorhynchus,Borodinopristis,(Columbusia,Onchopristis),Ctenopristis,Dalpiazia,Ganopristis,Ischyrhiza,Libanopristis,Marckgrafia,Micropristis,Plicatopristis,Sclerorhynchus,Kiestus,Atlanticopristis),Biropristis,Baharipristis,Renpetia,Celtipristis),Ptychotrygon,Ptychotrygonoides,Texatrygon,Archingeayia,Duwibatis,Engolismaia,Erguitaia,Erythrobatis,Hamrabatis,Safagaia,Tanoutia,Turoniabatis,Leiribatos,Phosphatobatis,Iberotrygon),(Pseudocetorhinus,Hueneichthys,Grozonodon,(((((((((((Etmopterus,Miroscyllium),Eoetmopterus,Microetmopterus,Paraetmopterus,Proetmopterus),(Centroscyllium,Aculeola),Trigonognathus),((Centroselachus,Proscymnodon,(Scymnodon,Zameus),Scymnodalatias,Cretascymnus,Centroscymnus),(SomniosusS,SomniosusR)),(Oxynotus,Protoxynotus),((Eosqualiolus,Squaliolus,Euprotomicrus),Heteroscymnoides,Paraphorosoides,Acrosqualiolus,Angoumeius,Squaliodalatias,Dalatias,Isistius,Euprotomicroides,Mollisquama)),(Protocentrophorus,(Centrophorus,Deania))),((Squalus,Cirrhigaleus,Protosqualus,Centrophoroides),Centrosqualus,Megasqualus)),(Squatina,Pseudorhina)),(Pristiophorus,(Ikamauius,Pliotrema))),(Heterodontus,Paracestracion,Proheterodontus)),(Echinorhinus,Gibbechinorhinus,Orthechinorhinus,Paraechinorhinus,Pseudoechinorhinus),(((Hexanchus,(Heptranchias,Paraheptranchias),Weltonia),Notorynchus,Notidanoides,Notidanodon,Pachyhexanchus,(Pseudonotidanus,Welcommia)),(Chlamydoselachus,Eothrinax),Sphenodus,Paraorthacodus),Protospinax),(((Dwardius,Priscusurus,Eoptolamna,(Mitsukurina,Scapanorhynchus,Anomotodon,Striatolamia,Woellsteinia),((Acrolamna,Archaeolamna,Cretodus,Cretoxyrhina,Dallasiela,Trigonotodus,Eostriatolamia,Paraisurus,Palaeocarcharodon,Cretolamna,Telodontaspis),Protolamna,Leptostyrax,Pseudoscapanorhynchus),((((Carcharias,Hispidaspis),Araloselachus),Brachycarcharias,Cenocarcharias,Glueckmanotodus,Hypotodus,Jaekelotodus,Johnlongia,(Mennerotodus,Borealotodus),Odontaspis,Orpodon,Palaeohypotodus,Roulletia,Sylvestrilamia,Turania,Pueblocarcharias),((Alopias,Anotodus,Paranomotodon,Usakias),Pseudocarcharias,Megachasma),((OtodusO,OtodusC,OtodusM),Parotodus),Cardabiodon,Serratolamna,((Galeocorax,Pseudocorax),(Ptychocorax,Nanocorax,Squalicorax,Scindocorax)),(Cetorhinus,(((Carchariolamna,Lamna,Carcharoides),Isurolamna,(Isurus,Macrorhizodus)),(Carcharodon,Cosmopolitodus),Karaisurus,Lethenia,Xiphodolamia)))),(Palaeorectolobus,Agaleus,Palaeocarcharias,Pseudospinax,(Annea,Dorsetoscyllium,Folipistrix,(Phorcynis,Heterophorcynus),Ornatoscyllium,(Pararhincodon,Cirrhoscyllium,Parascyllium),((((Ginglymostoma,Plicatoscyllium),Cantioscyllium,Protoginglymostoma,(Palaeorhincodon,Rhincodon),Stegostoma,Pseudoginglymostoma,Nebrius,Delpitoscyllium,Ganntouria,Hologinglymostoma),(Acanthoscyllium,Almascyllium,Hemiscyllium,Chiloscyllium,Mesiteia)),(Palaeobrachaelurus,Cretorectolobus,(Paraginglymostoma,Brachaelurus,Eostegostoma,Heteroscyllium),(Cederstroemia,Orectoloboides,(Eometlaouia,Squatiscyllium),Orectolobus,Eucrossorhinus,Sutorectus)))))),((Bavariscyllium,Casieria,Cretascyliorhinus,Eypea,(Foumtizia,Pachyscyllium),Macrourogaleus,Megascyliorhinus,Microscyliorhinus,Crassescyliorhinus,Palaeoscyllium,Platyrhizoscyllium,(PremontreiaP,PremontreiaO),Protoscyliorhinus,Pseudoscyliorhinus,Pteroscyllium,Stenoscyllium,Schroederichthys,(((Porodermoides,Poroderma),(Scyliorhinus,Cephaloscyllium)),(Atelomycterus,Aulohalaelurus)),(Galeus,Bythaelurus,(Asymbolus,(Cephalurus,Parmaturus)),(Holohalaelurus,Halaelurus,Haploblepharus,Prohaploblepharus),(Pentanchus,Apristurus)),(Proscyllium,Praeproscyllium,Ctenacis,Eridacnis),((Pseudotriakis,Gollum),(Leptocharias,(Archaeotriakis,(Iago,Khouribgaleus,Gomphogaleus,Galeorhinus,(Triakis,Scylliogaleus,Mustelus,Hemitriakis,Furgaleus,Pachygaleus,Palaeogaleus,Paratriakis,Squatigaleus,Xystrogaleus,((Hemigaleus,Paragaleus,(Chaenogaleus,Hemipristis),Moerigaleus),((Sphyrna,Eusphyra),((Abdounia,Tingaleus),Danogaleus,Eogaleus,Physogaleus,(Rhizoprionodon,Scoliodon,Loxodon,Isogomphodon,(Triaenodon,((Glyphis,Lamiopsis),Prionace,Nasolamia,Carcharhinus))),Kruckowlamna,Misrichthys,Negaprion,Galeocerdo)))))))),Figaro,Gogolia),Corysodon)),Parasquatina,(Breviacanthus,(Mucrovenator,Rhomphaiodon),Palidiplospinax,Synechodus),Reifia))),Vallisia)

**MAW-m[16,30]**

(Vallisia,(((((((((((Burnhamia,Eomobula,(Eoplinthicus,Plinthicus),Manta,Mobula),Rhinoptera),Ixobatis,(Pucabatis,Rhombodus,Washakiebatis),Aetobatus,Aetomylaeus,Apocopodon,Brachyrhizodus,Garabatis,Igdabatis,Leidybatis,Lophobatis,(Myliobatis,Pteromylaeus),Pseudaetobatus,Aktaua),(Pteroplatea,Aetoplatea,Gymnura,Jacquhermania,Ouledia),Cretomanta,Archaeomanta),((Urolophus,Trygonoptera,Plesiobatis),Himantura),((Potamotrygon,Plesiotrygon),(Paratrygon,Heliotrygon)),Arechia,Aturobatis,Coupatezia,Dasyrhombodus,Delpitia,Enantiobatis,Ewingia,Heterobatis,Heterotorpedo,Merabatis,Myliodasyatis,Phosphatodon,Meridiania,Texabatis,Viperecucullus,(Urotrygon,Urobatis),Hypolophites,Hypolophodon,Ishaquia,Pastinachus,Taeniura,Dasyatis,Neotrygon,Pteroplatytrygon,Taeniurops,Urogymnus,Asterotrygon,Heliobatis),Hexatrygon),Zanobatus),(Ataktobatis,Engaibatis,Jurobatos,Microbatis,Myledaphus,Protoplatyrhina,Pseudohypolophus,Squatirhina,Vascobatis,(Rhina,Rhynchobatus,((Asterodermus,Belemnobatis),Doliobatis,Iansan,Isidobatus,Paratrygonorrhina,Rhinobatos,Rhombopterygia,Spathobatis,Aptychotrema,Trygonorrhina,Zapteryx,Platypterix),(Platyrhina,Tethybatis,Platyrhinoidis),(Hypsobatis,Angolabatis,Youssoubatis),Parapalaeobates))),(((Torpedo,Eotorpedo),Hypnos),((Heteronarce,Typhlonarke,Narke,Temera,Crassinarke,Electrolux),(Narcine,Benthobatis,Diplobatis,Discopyge),Titanonarke))),(((Dipturus,Raja,Malacoraja,Mafdetia,Rajorhina,Smithraja,Walteraja,Amblyraja,Breviraja,Dactylobatus,Dentiraja,Fenestraja,Gurgesiella,Hongeo,Insentiraja,Leucoraja,Neoraja,Okamejei,Rajella,Rostroraja,Zearaja),(Anacanthobatis,Sinobatis),(Psammobatis,Pseudoraja,Rioraja,Rhinoraja,Pavoraja,Notoraja,Irolita,Brochiraja,Bathyraja,Atlantoraja,Arhynchobatis,Sympterygia)),Cyclobatis),(Anoxypristis,Pristis,Propristis),(Cristabatis,Toarcibatis),((Onchosaurus,Pucapristis,Schizorhiza),(Ankistrorhynchus,Borodinopristis,(Columbusia,Onchopristis),Ctenopristis,Dalpiazia,Ganopristis,Ischyrhiza,Libanopristis,Marckgrafia,Micropristis,Plicatopristis,Sclerorhynchus,Kiestus,Atlanticopristis),Biropristis,Baharipristis,Renpetia,Celtipristis),Ptychotrygon,Ptychotrygonoides,Texatrygon,Archingeayia,Duwibatis,Engolismaia,Erguitaia,Erythrobatis,Hamrabatis,Safagaia,Tanoutia,Turoniabatis,Leiribatos,Phosphatobatis,Iberotrygon),(Pseudocetorhinus,Hueneichthys,Grozonodon,(((((((((Etmopterus,Miroscyllium),Eoetmopterus,Microetmopterus,Paraetmopterus,Proetmopterus),(Centroscyllium,Aculeola),Trigonognathus),((Centroselachus,Proscymnodon,(Scymnodon,Zameus),Scymnodalatias,Cretascymnus,Centroscymnus),(SomniosusS,SomniosusR)),(Oxynotus,Protoxynotus),((Eosqualiolus,Squaliolus,Euprotomicrus),Heteroscymnoides,Paraphorosoides,Acrosqualiolus,Angoumeius,Squaliodalatias,Dalatias,Isistius,Euprotomicroides,Mollisquama)),(Protocentrophorus,(Centrophorus,Deania))),((Squalus,Cirrhigaleus,Protosqualus,Centrophoroides),Centrosqualus,Megasqualus)),((Pristiophorus,(Ikamauius,Pliotrema)),(Squatina,Pseudorhina))),(Echinorhinus,Gibbechinorhinus,Orthechinorhinus,Paraechinorhinus,Pseudoechinorhinus),(((Hexanchus,(Heptranchias,Paraheptranchias),Weltonia),Notorynchus,Notidanoides,Notidanodon,Pachyhexanchus,(Pseudonotidanus,Welcommia)),(Chlamydoselachus,Eothrinax),Sphenodus,Paraorthacodus),Protospinax),(Reifia,(Breviacanthus,(Mucrovenator,Rhomphaiodon),Palidiplospinax,Synechodus),((Heterodontus,Paracestracion,Proheterodontus),(((Palaeorectolobus,Agaleus,Palaeocarcharias,Pseudospinax,(Annea,Dorsetoscyllium,Folipistrix,(Phorcynis,Heterophorcynus),Ornatoscyllium,(Pararhincodon,Cirrhoscyllium,Parascyllium),((((Ginglymostoma,Plicatoscyllium),Cantioscyllium,Protoginglymostoma,(Palaeorhincodon,Rhincodon),Stegostoma,Pseudoginglymostoma,Nebrius,Delpitoscyllium,Ganntouria,Hologinglymostoma),(Acanthoscyllium,Almascyllium,Hemiscyllium,Chiloscyllium,Mesiteia)),(Palaeobrachaelurus,Cretorectolobus,(Paraginglymostoma,Brachaelurus,Eostegostoma,Heteroscyllium),(Cederstroemia,Orectoloboides,(Eometlaouia,Squatiscyllium),Orectolobus,Eucrossorhinus,Sutorectus))))),((Bavariscyllium,Casieria,Cretascyliorhinus,Eypea,(Foumtizia,Pachyscyllium),Macrourogaleus,Megascyliorhinus,Microscyliorhinus,Crassescyliorhinus,Palaeoscyllium,Platyrhizoscyllium,(PremontreiaP,PremontreiaO),Protoscyliorhinus,Pseudoscyliorhinus,Pteroscyllium,Stenoscyllium,Schroederichthys,(((Porodermoides,Poroderma),(Scyliorhinus,Cephaloscyllium)),(Atelomycterus,Aulohalaelurus)),(Galeus,Bythaelurus,(Asymbolus,(Cephalurus,Parmaturus)),(Holohalaelurus,Halaelurus,Haploblepharus,Prohaploblepharus),(Pentanchus,Apristurus)),(Proscyllium,Praeproscyllium,Ctenacis,Eridacnis),((Pseudotriakis,Gollum),(Leptocharias,(Archaeotriakis,(Iago,Khouribgaleus,Gomphogaleus,Galeorhinus,(Triakis,Scylliogaleus,Mustelus,Hemitriakis,Furgaleus,Pachygaleus,Palaeogaleus,Paratriakis,Squatigaleus,Xystrogaleus,((Hemigaleus,Paragaleus,(Chaenogaleus,Hemipristis),Moerigaleus),((Sphyrna,Eusphyra),((Abdounia,Tingaleus),Danogaleus,Eogaleus,Physogaleus,(Rhizoprionodon,Scoliodon,Loxodon,Isogomphodon,(Triaenodon,((Glyphis,Lamiopsis),Prionace,Nasolamia,Carcharhinus))),Kruckowlamna,Misrichthys,Negaprion,Galeocerdo)))))))),Figaro,Gogolia),Corysodon)),(Dwardius,Priscusurus,Eoptolamna,(Mitsukurina,Scapanorhynchus,Anomotodon,Striatolamia,Woellsteinia),((Acrolamna,Archaeolamna,Cretodus,Cretoxyrhina,Dallasiela,Trigonotodus,Eostriatolamia,Paraisurus,Palaeocarcharodon,Cretolamna,Telodontaspis),Protolamna,Leptostyrax,Pseudoscapanorhynchus),((((Carcharias,Hispidaspis),Araloselachus),Brachycarcharias,Cenocarcharias,Glueckmanotodus,Hypotodus,Jaekelotodus,Johnlongia,(Mennerotodus,Borealotodus),Odontaspis,Orpodon,Palaeohypotodus,Roulletia,Sylvestrilamia,Turania,Pueblocarcharias),((Alopias,Anotodus,Paranomotodon,Usakias),Pseudocarcharias,Megachasma),((OtodusO,OtodusC,OtodusM),Parotodus),Cardabiodon,Serratolamna,((Galeocorax,Pseudocorax),(Ptychocorax,Nanocorax,Squalicorax,Scindocorax)),(Cetorhinus,(((Carchariolamna,Lamna,Carcharoides),Isurolamna,(Isurus,Macrorhizodus)),(Carcharodon,Cosmopolitodus),Karaisurus,Lethenia,Xiphodolamia))))))),Parasquatina))),((((((Aegyptobatus,Tribodus),Distobatus,Glickmanodus,Reticulodus,Acrodus,Acrorhizodus,Asteracanthus,Bdellodus),(Egertonodus,Pororhiza,Priohybodus,Thaiodus,Leptacanthus,Planohybodus,Secarodus,Hybodus,Meristodon,Meristodonoides,Khoratodus)),(Palaeobates,Polyacrodus)),(Lonchidion,Lissodus,Bahariyodon,Hylaeobatis,Isanodus,Parvodus,Vectiselachos,Jiaodontus,Diplolonchidion)),(Homalodontus,Doratodus),(Heteroptychodus,Ptychodus),Steinbachodus,Pseudodalatias,Ostenoselache,Hubeiodus,Mukdahanodus,Raineria))

**NAY[31]**

(((((((Aegyptobatus,Tribodus),Distobatus,Glickmanodus,Reticulodus,Acrodus,Acrorhizodus,Asteracanthus,Bdellodus),(Egertonodus,Pororhiza,Priohybodus,Thaiodus,Leptacanthus,Planohybodus,Secarodus,Hybodus,Meristodon,Meristodonoides,Khoratodus)),(Palaeobates,Polyacrodus)),(Lonchidion,Lissodus,Bahariyodon,Hylaeobatis,Isanodus,Parvodus,Vectiselachos,Jiaodontus,Diplolonchidion)),(Homalodontus,Doratodus),(Heteroptychodus,Ptychodus),Steinbachodus,Pseudodalatias,Ostenoselache,Hubeiodus,Mukdahanodus,Raineria),(((((Pteroplatea,Aetoplatea,Gymnura,Jacquhermania,Ouledia),(Trygonoptera,Urolophus),Plesiobatis,Hexatrygon),(((Potamotrygon,Plesiotrygon),(Paratrygon,Heliotrygon)),(Urotrygon,Urobatis),Arechia,Aturobatis,Coupatezia,Dasyrhombodus,Delpitia,Enantiobatis,Ewingia,Heterobatis,Heterotorpedo,Merabatis,Myliodasyatis,Phosphatodon,Meridiania,Texabatis,Viperecucullus,Hypolophodon,Neotrygon,(Asterotrygon,Dasyatis,Heliobatis,Himantura,Hypolophites,Ishaquia,Pastinachus,Pteroplatytrygon,Urogymnus,Taeniurops,Taeniura)),(((Burnhamia,Eomobula,(Eoplinthicus,Plinthicus),Manta,Mobula),Rhinoptera),Ixobatis,(Pucabatis,Rhombodus,Washakiebatis),Aetobatus,Aetomylaeus,Apocopodon,Brachyrhizodus,Garabatis,Igdabatis,Leidybatis,Lophobatis,(Myliobatis,Pteromylaeus),Pseudaetobatus,Aktaua),Archaeomanta,Cretomanta),((((Dipturus,Raja,Malacoraja,Mafdetia,Rajorhina,Smithraja,Walteraja,Amblyraja,Breviraja,Dactylobatus,Dentiraja,Fenestraja,Gurgesiella,Hongeo,Insentiraja,Leucoraja,Neoraja,Okamejei,Rajella,Rostroraja,Zearaja),(Psammobatis,Pseudoraja,Rioraja,Rhinoraja,Pavoraja,Notoraja,Irolita,Brochiraja,Bathyraja,Atlantoraja,Arhynchobatis,Sympterygia)),(Anacanthobatis,Sinobatis)),Cyclobatis),((Heteronarce,Typhlonarke,Narke,Temera,Crassinarke,Electrolux),(Narcine,Benthobatis,Diplobatis,Discopyge),Titanonarke,(Torpedo,Eotorpedo),Hypnos),Ataktobatis,Engaibatis,Jurobatos,Microbatis,Myledaphus,Protoplatyrhina,Pseudohypolophus,Squatirhina,Vascobatis,(Rhina,Rhynchobatus),(Platyrhina,Tethybatis,Platyrhinoidis),(Hypsobatis,Angolabatis,Youssoubatis),Parapalaeobates,(Asterodermus,Belemnobatis),Doliobatis,Iansan,Isidobatus,Paratrygonorrhina,Rhombopterygia,Spathobatis,(Trygonorrhina,(Zapteryx,Aptychotrema)),Platypterix,Zanobatus,(Cristabatis,Toarcibatis),Rhinobatos,Anoxypristis,Pristis,Propristis,((Onchosaurus,Pucapristis,Schizorhiza),(Ankistrorhynchus,Borodinopristis,(Columbusia,Onchopristis),Ctenopristis,Dalpiazia,Ganopristis,Ischyrhiza,Libanopristis,Marckgrafia,Micropristis,Plicatopristis,Sclerorhynchus,Kiestus,Atlanticopristis),Biropristis,Baharipristis,Renpetia,Celtipristis),Ptychotrygon,Ptychotrygonoides,Texatrygon,Archingeayia,Duwibatis,Engolismaia,Erguitaia,Erythrobatis,Hamrabatis,Safagaia,Tanoutia,Turoniabatis,Leiribatos,Phosphatobatis,Iberotrygon),((Parasquatina,(((((((Eosqualiolus,Squaliolus,Euprotomicrus),Heteroscymnoides,Paraphorosoides,Acrosqualiolus,Angoumeius,Squaliodalatias,(Isistius,Dalatias),Euprotomicroides,Mollisquama),((Squalus,Cirrhigaleus,Protosqualus,Centrophoroides),Centrosqualus,Megasqualus)),(((Oxynotus,Protoxynotus),Proscymnodon,Scymnodalatias,Cretascymnus,Centroscymnus,Scymnodon,(Zameus,Centroselachus)),(SomniosusS,SomniosusR)),((Eoetmopterus,Microetmopterus,Paraetmopterus,Proetmopterus,Etmopterus,Miroscyllium),(Trigonognathus,Centroscyllium),Aculeola)),(Protocentrophorus,(Centrophorus,Deania))),(((Pristiophorus,(Ikamauius,Pliotrema)),(Squatina,Pseudorhina)),(Echinorhinus,Gibbechinorhinus,Orthechinorhinus,Paraechinorhinus,Pseudoechinorhinus))),Protospinax,((Chlamydoselachus,Eothrinax),Sphenodus,Paraorthacodus,((Hexanchus,Weltonia,Heptranchias,Paraheptranchias),Notorynchus,Notidanoides,Notidanodon,Pachyhexanchus),(Pseudonotidanus,Welcommia))),((Breviacanthus,(Mucrovenator,Rhomphaiodon),Palidiplospinax,Synechodus),Reifia,((Heterodontus,Paracestracion,Proheterodontus),((((((Acanthoscyllium,Almascyllium,Hemiscyllium,Chiloscyllium,Mesiteia),((Ginglymostoma,Plicatoscyllium),Cantioscyllium,Protoginglymostoma,(Palaeorhincodon,Rhincodon),Stegostoma,Pseudoginglymostoma,Nebrius,Delpitoscyllium,Ganntouria,Hologinglymostoma)),(Palaeobrachaelurus,Cretorectolobus,(Paraginglymostoma,Brachaelurus,Eostegostoma,Heteroscyllium),(Cederstroemia,Orectoloboides,(Eometlaouia,Squatiscyllium),Orectolobus,Eucrossorhinus,Sutorectus))),(Pararhincodon,Cirrhoscyllium,Parascyllium),Annea,Dorsetoscyllium,Folipistrix,(Phorcynis,Heterophorcynus),Ornatoscyllium),Agaleus,Palaeorectolobus,Palaeocarcharias,Pseudospinax),((Dwardius,Priscusurus,Eoptolamna,(Mitsukurina,Scapanorhynchus,Anomotodon,Striatolamia,Woellsteinia),((Acrolamna,Archaeolamna,Cretodus,Cretoxyrhina,Dallasiela,Trigonotodus,Eostriatolamia,Paraisurus,Palaeocarcharodon,Cretolamna,Telodontaspis),Protolamna,Leptostyrax,Pseudoscapanorhynchus),((((Carcharias,Hispidaspis),Araloselachus),Brachycarcharias,Cenocarcharias,Glueckmanotodus,Hypotodus,Jaekelotodus,Johnlongia,(Mennerotodus,Borealotodus),Odontaspis,Orpodon,Palaeohypotodus,Roulletia,Sylvestrilamia,Turania,Pueblocarcharias),(Pseudocarcharias,Megachasma,Alopias,Anotodus,Paranomotodon,Usakias),((OtodusO,OtodusC,OtodusM),Parotodus),Cardabiodon,Serratolamna,((Galeocorax,Pseudocorax),(Ptychocorax,Nanocorax,Squalicorax,Scindocorax)),(Cetorhinus,(Carcharodon,Cosmopolitodus),Karaisurus,Lethenia,Xiphodolamia,(Carchariolamna,Lamna,Carcharoides),Isurolamna,(Isurus,Macrorhizodus)))),((Iago,Khouribgaleus,Gomphogaleus,Galeorhinus,Triakis,Scylliogaleus,Mustelus,(Hemitriakis,Furgaleus),Pachygaleus,Palaeogaleus,Paratriakis,Squatigaleus,Xystrogaleus,(Leptocharias,((Hemigaleus,Paragaleus,(Chaenogaleus,Hemipristis),Moerigaleus),((Sphyrna,Eusphyra),((((Scoliodon,Loxodon),Rhizoprionodon),((Triaenodon,Nasolamia,Prionace,Carcharhinus),Isogomphodon),(Glyphis,Lamiopsis),Negaprion),Kruckowlamna,Misrichthys,Galeocerdo,(Abdounia,Tingaleus),Danogaleus,Eogaleus,Physogaleus)))),Archaeotriakis),(((Porodermoides,Poroderma),Scyliorhinus),Cephaloscyllium),Parmaturus,Galeus,(Pentanchus,Apristurus),Proscyllium,Praeproscyllium,Ctenacis,((Pseudotriakis,Gollum),Eridacnis),Bavariscyllium,Casieria,Cretascyliorhinus,Eypea,(Foumtizia,Pachyscyllium),Macrourogaleus,Megascyliorhinus,Microscyliorhinus,Crassescyliorhinus,Palaeoscyllium,Platyrhizoscyllium,(PremontreiaP,PremontreiaO),Protoscyliorhinus,Pseudoscyliorhinus,Pteroscyllium,Stenoscyllium,((Figaro,Asymbolus),Bythaelurus),Gogolia,Schroederichthys,Atelomycterus,Aulohalaelurus,(Halaelurus,Haploblepharus,Prohaploblepharus),Holohalaelurus,Cephalurus,Corysodon)))))),Pseudocetorhinus,Hueneichthys,Grozonodon,Vallisia)))

*Family-level phylogenies:*

**DOU[30]**

(((Lonchidiidae,(Polyacrodontidae,(Hybodontidae,(Acrodontidae,Distobatidae)))),Ostenoselachidae,Ptychodontidae,Steinbachodontidae,Pseudodalatiidae,Waipitiodidae),((((((((Rhinopteridae,Mobulidae),Rhombodontidae,Myliobatidae),Gymnuridae),(Urolophidae,Plesiobatidae),Potamotrygonidae,Dasyatidae,Urotrygonidae),Hexatrygonidae)),((Narkidae,Narcinidae),(Torpedinidae,Hypnidae)),Pristidae,Archaeobatidae,Sclerorhynchidae,((Rhynchobatidae,Rhinidae,Rhinobatidae,Platyrhinidae,Parapalaeobatidae,Hypsobatidae)),((Anacanthobatidae,Rajidae,Arhynchobatidae),Cyclobatidae)),((((((((Squalidae,(Centrophoridae,Etmopteridae,Dalatiidae,Oxynotidae,Somniosidae)),(Pristiophoridae,Squatinidae)),(Chlamydoselachidae,Orthacodontidae,Paraorthacodontidae,Pseudonotidanidae,(Heptranchidae,Hexanchidae)),Echinorhinidae,Protospinacidae),((Eoptolamnidae,(Paraisuridae,Pseudoscapanorhynchidae,Cretoxyrhinidae,Archaeolamnidae),Mitsukurinidae,((Alopiidae,Pseudocarchariidae,Megachasmidae),Odontaspididae,Otodontidae,Cardabiodontidae,Serratolamnidae,(Anacoracidae,Pseudocoracidae),(Lamnidae,Cetorhinidae))),((Scyliorhinidae,((Proscylliidae,Pseudotriakidae),(Leptochariidae,(Triakidae,(Hemigaleidae,(Sphyrnidae,Carcharhinidae))))))))),((Parascylliidae,((Orectolobidae,Brachaeluridae),(Hemiscylliidae,(Rhincodontidae,Stegostomatidae,Ginglymostomatidae)))),Agaleidae)),Heterodontidae),Palaeospinacidae))))

**HEI[31]**

(((Lonchidiidae,(Polyacrodontidae,(Hybodontidae,(Acrodontidae,Distobatidae)))),Ostenoselachidae,Ptychodontidae,Steinbachodontidae,Pseudodalatiidae,Waipitiodidae),((((((((((Rhinopteridae,Mobulidae),Rhombodontidae,Myliobatidae),Gymnuridae),(Urolophidae,Plesiobatidae),Potamotrygonidae,Dasyatidae,Urotrygonidae),Hexatrygonidae)),((Rhynchobatidae,Rhinidae,Rhinobatidae,Platyrhinidae,Parapalaeobatidae,Hypsobatidae))),((Narkidae,Narcinidae),(Torpedinidae,Hypnidae))),((Anacanthobatidae,Rajidae,Arhynchobatidae),Cyclobatidae),Pristidae,Archaeobatidae,Sclerorhynchidae),(((Echinorhinidae,Protospinacidae,(((Squalidae,(Centrophoridae,Etmopteridae,Dalatiidae,Oxynotidae,Somniosidae)),Squatinidae),Pristiophoridae),(Chlamydoselachidae,Orthacodontidae,Paraorthacodontidae,Pseudonotidanidae,(Heptranchidae,Hexanchidae))),(Palaeospinacidae,(Heterodontidae,(((Parascylliidae,((Orectolobidae,Brachaeluridae),(Hemiscylliidae,(Rhincodontidae,Stegostomatidae,Ginglymostomatidae)))),Agaleidae),((Eoptolamnidae,(Paraisuridae,Pseudoscapanorhynchidae,Cretoxyrhinidae,Archaeolamnidae),Mitsukurinidae,((Alopiidae,Pseudocarchariidae,Megachasmidae),Odontaspididae,Otodontidae,Cardabiodontidae,Serratolamnidae,(Anacoracidae,Pseudocoracidae),(Lamnidae,Cetorhinidae))),((Scyliorhinidae,((Proscylliidae,Pseudotriakidae),(Leptochariidae,(Triakidae,(Hemigaleidae,(Sphyrnidae,Carcharhinidae)))))))))))))))


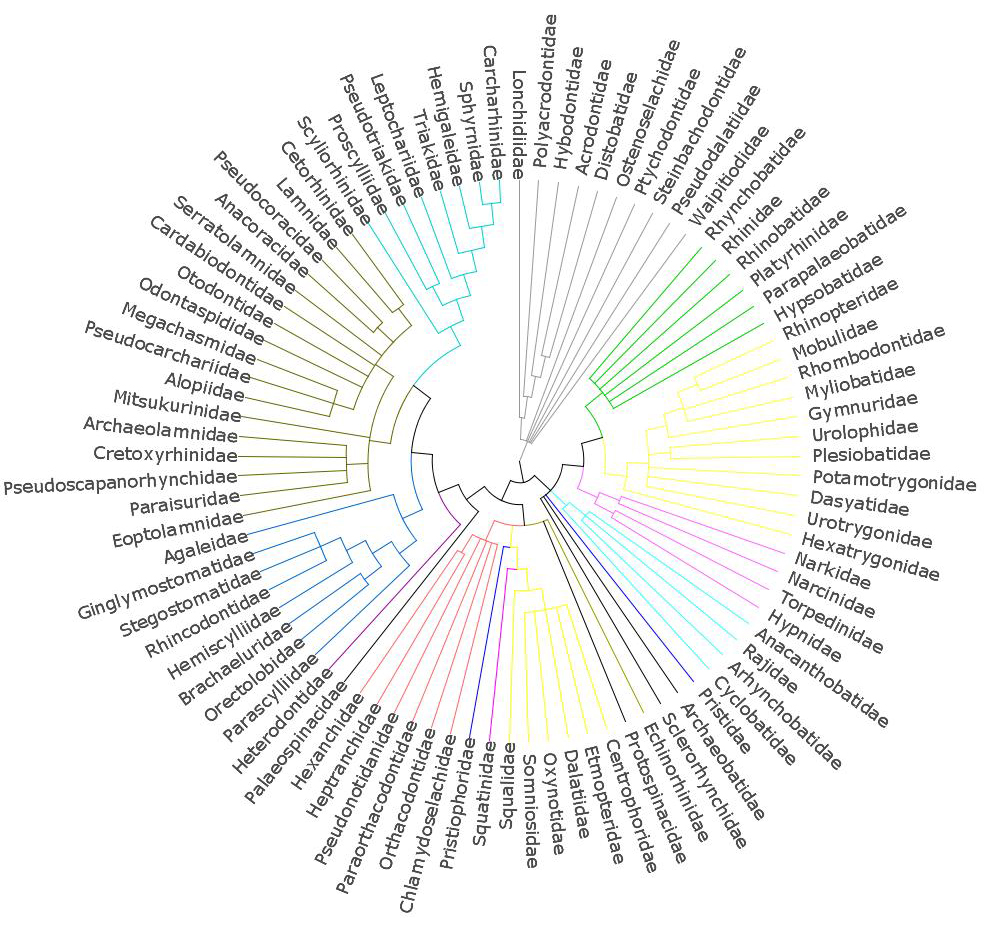


**HUM[32]**

(((Lonchidiidae,(Polyacrodontidae,(Hybodontidae,(Acrodontidae,Distobatidae)))),Ostenoselachidae,Ptychodontidae,Steinbachodontidae,Pseudodalatiidae,Waipitiodidae),(((((((((Rhinopteridae,Mobulidae),Rhombodontidae,Myliobatidae),Gymnuridae),(Urolophidae,Plesiobatidae),Potamotrygonidae,Dasyatidae,Urotrygonidae),Hexatrygonidae)),((Anacanthobatidae,Rajidae,Arhynchobatidae),Cyclobatidae)),((Narkidae,Narcinidae),(Torpedinidae,Hypnidae)),((Rhynchobatidae,Rhinidae,Rhinobatidae,Platyrhinidae,Parapalaeobatidae,Hypsobatidae)),Pristidae,Archaeobatidae,Sclerorhynchidae),((((((Parascylliidae,((Orectolobidae,Brachaeluridae),(Hemiscylliidae,(Rhincodontidae,Stegostomatidae,Ginglymostomatidae)))),Agaleidae),(((Pristiophoridae,Squatinidae),(Squalidae,(Centrophoridae,Etmopteridae,Dalatiidae,Oxynotidae,Somniosidae))),Heterodontidae),((Eoptolamnidae,(Paraisuridae,Pseudoscapanorhynchidae,Cretoxyrhinidae,Archaeolamnidae),Mitsukurinidae,((Alopiidae,Pseudocarchariidae,Megachasmidae),Odontaspididae,Otodontidae,Cardabiodontidae,Serratolamnidae,(Anacoracidae,Pseudocoracidae),(Lamnidae,Cetorhinidae))),((Scyliorhinidae,((Proscylliidae,Pseudotriakidae),(Leptochariidae,(Triakidae,(Hemigaleidae,(Sphyrnidae,Carcharhinidae))))))))),(Chlamydoselachidae,Orthacodontidae,Paraorthacodontidae,Pseudonotidanidae,(Heptranchidae,Hexanchidae)),Protospinacidae,Echinorhinidae),Palaeospinacidae))))

**MAI[33]**

(((Lonchidiidae,(Polyacrodontidae,(Hybodontidae,(Acrodontidae,Distobatidae)))),Ostenoselachidae,Ptychodontidae,Steinbachodontidae,Pseudodalatiidae,Waipitiodidae),(((Pristidae,((((((Rhinopteridae,Mobulidae),Rhombodontidae,Myliobatidae),Gymnuridae),(Urolophidae,Plesiobatidae),Potamotrygonidae,Dasyatidae,Urotrygonidae),Hexatrygonidae))),((Narkidae,Narcinidae),(Torpedinidae,Hypnidae)),(((Rhynchobatidae,Rhinidae,Rhinobatidae,Platyrhinidae,Parapalaeobatidae,Hypsobatidae)),((Anacanthobatidae,Rajidae,Arhynchobatidae),Cyclobatidae)),Archaeobatidae,Sclerorhynchidae),(((Protospinacidae,(Chlamydoselachidae,Orthacodontidae,Paraorthacodontidae,Pseudonotidanidae,(Heptranchidae,Hexanchidae)),(((Echinorhinidae,Squatinidae),(Squalidae,(Centrophoridae,Etmopteridae,Dalatiidae,Oxynotidae,Somniosidae))),Pristiophoridae)),(Palaeospinacidae,(Heterodontidae,(((Parascylliidae,((Orectolobidae,Brachaeluridae),(Hemiscylliidae,(Rhincodontidae,Stegostomatidae,Ginglymostomatidae)))),Agaleidae),((Eoptolamnidae,(Paraisuridae,Pseudoscapanorhynchidae,Cretoxyrhinidae,Archaeolamnidae),Mitsukurinidae,((Alopiidae,Pseudocarchariidae,Megachasmidae),Odontaspididae,Otodontidae,Cardabiodontidae,Serratolamnidae,(Anacoracidae,Pseudocoracidae),(Lamnidae,Cetorhinidae))),((Scyliorhinidae,((Proscylliidae,Pseudotriakidae),(Leptochariidae,(Triakidae,(Hemigaleidae,(Sphyrnidae,Carcharhinidae)))))))))))))))

**MAW[34]**

(((Lonchidiidae,(Polyacrodontidae,(Hybodontidae,(Acrodontidae,Distobatidae)))),Ostenoselachidae,Ptychodontidae,Steinbachodontidae,Pseudodalatiidae,Waipitiodidae),((((((((((Rhinopteridae,Mobulidae),Rhombodontidae,Myliobatidae),Gymnuridae),(Urolophidae,Plesiobatidae),Potamotrygonidae,Dasyatidae,Urotrygonidae),Hexatrygonidae)),((Rhynchobatidae,Rhinidae,Rhinobatidae,Platyrhinidae,Parapalaeobatidae,Hypsobatidae))),((Narkidae,Narcinidae),(Torpedinidae,Hypnidae))),Pristidae,Archaeobatidae,Sclerorhynchidae,((Anacanthobatidae,Rajidae,Arhynchobatidae),Cyclobatidae)),(((((Chlamydoselachidae,Orthacodontidae,Paraorthacodontidae,Pseudonotidanidae,(Heptranchidae,Hexanchidae)),(((Squalidae,(Centrophoridae,Etmopteridae,Dalatiidae,Oxynotidae,Somniosidae)),Squatinidae),Pristiophoridae),Echinorhinidae,Protospinacidae),Heterodontidae),Palaeospinacidae,(((Eoptolamnidae,(Paraisuridae,Pseudoscapanorhynchidae,Cretoxyrhinidae,Archaeolamnidae),Mitsukurinidae,((Alopiidae,Pseudocarchariidae,Megachasmidae),Odontaspididae,Otodontidae,Cardabiodontidae,Serratolamnidae,(Anacoracidae,Pseudocoracidae),(Lamnidae,Cetorhinidae))),((Parascylliidae,((Orectolobidae,Brachaeluridae),(Hemiscylliidae,(Rhincodontidae,Stegostomatidae,Ginglymostomatidae)))),Agaleidae)),((Scyliorhinidae,((Proscylliidae,Pseudotriakidae),(Leptochariidae,(Triakidae,(Hemigaleidae,(Sphyrnidae,Carcharhinidae))))))))))))

**MAW-m[34,20]**

(((Lonchidiidae,(Polyacrodontidae,(Hybodontidae,(Acrodontidae,Distobatidae)))),Ostenoselachidae,Ptychodontidae,Steinbachodontidae,Pseudodalatiidae,Waipitiodidae),((((((((((Rhinopteridae,Mobulidae),Rhombodontidae,Myliobatidae),Gymnuridae),(Urolophidae,Plesiobatidae),Potamotrygonidae,Dasyatidae,Urotrygonidae),Hexatrygonidae)),((Rhynchobatidae,Rhinidae,Rhinobatidae,Platyrhinidae,Parapalaeobatidae,Hypsobatidae))),((Narkidae,Narcinidae),(Torpedinidae,Hypnidae))),Pristidae,Archaeobatidae,Sclerorhynchidae,((Anacanthobatidae,Rajidae,Arhynchobatidae),Cyclobatidae)),((((Chlamydoselachidae,Orthacodontidae,Paraorthacodontidae,Pseudonotidanidae,(Heptranchidae,Hexanchidae)),((Pristiophoridae,Squatinidae),(Squalidae,(Centrophoridae,Etmopteridae,Dalatiidae,Oxynotidae,Somniosidae))),Echinorhinidae,Protospinacidae),(Palaeospinacidae,(Heterodontidae,((Eoptolamnidae,(Paraisuridae,Pseudoscapanorhynchidae,Cretoxyrhinidae,Archaeolamnidae),Mitsukurinidae,((Alopiidae,Pseudocarchariidae,Megachasmidae),Odontaspididae,Otodontidae,Cardabiodontidae,Serratolamnidae,(Anacoracidae,Pseudocoracidae),(Lamnidae,Cetorhinidae))),(((Scyliorhinidae,((Proscylliidae,Pseudotriakidae),(Leptochariidae,(Triakidae,(Hemigaleidae,(Sphyrnidae,Carcharhinidae))))))),((Parascylliidae,((Orectolobidae,Brachaeluridae),(Hemiscylliidae,(Rhincodontidae,Stegostomatidae,Ginglymostomatidae)))),Agaleidae)))))))))

**NAY[31]**

((((Rhynchobatidae,Rhinidae,Rhinobatidae,Parapalaeobatidae,Hypsobatidae,Pristidae),Archaeobatidae,Sclerorhynchidae,((Urolophidae,Plesiobatidae,Gymnuridae,Hexatrygonidae),(Potamotrygonidae,Dasyatidae,Urotrygonidae),((Rhinopteridae,Mobulidae),Rhombodontidae,Myliobatidae)),((Anacanthobatidae,(Rajidae,Arhynchobatidae)),Cyclobatidae),(Narkidae,Narcinidae,Torpedinidae,Hypnidae),Platyrhinidae),((((Centrophoridae,(Etmopteridae,(Dalatiidae,Squalidae),(Oxynotidae,Somniosidae))),((Squatinidae,Pristiophoridae),Echinorhinidae)),Protospinacidae,(Chlamydoselachidae,Orthacodontidae,Paraorthacodontidae,Pseudonotidanidae,(Heptranchidae,Hexanchidae))),(Palaeospinacidae,(Heterodontidae,(((Parascylliidae,((Orectolobidae,Brachaeluridae),(Hemiscylliidae,(Rhincodontidae,Stegostomatidae,Ginglymostomatidae)))),Agaleidae),((Eoptolamnidae,(Paraisuridae,Pseudoscapanorhynchidae,Cretoxyrhinidae,Archaeolamnidae),Mitsukurinidae,(Odontaspididae,Otodontidae,Cardabiodontidae,Serratolamnidae,(Anacoracidae,Pseudocoracidae),Lamnidae,Cetorhinidae,Alopiidae,Pseudocarchariidae,Megachasmidae)),(Proscylliidae,Pseudotriakidae,Scyliorhinidae,(Triakidae,(Leptochariidae,(Hemigaleidae,(Sphyrnidae,Carcharhinidae))))))))))),((Lonchidiidae,(Polyacrodontidae,(Hybodontidae,(Acrodontidae,Distobatidae)))),Ostenoselachidae,Ptychodontidae,Steinbachodontidae,Pseudodalatiidae,Waipitiodidae))

*Order-level phylogenies:*

**DOU[30]**

(Hybodontiformes,((Torpediniformes,Rajiformes^1^, Rhinobatid-like^2^,Myliobatiformes, Pristids^3^,Archaeobatidae,Sclerorhynchidae),((((((Lamniformes,Carcharhiniformes),((Squaliformes,(Pristiophoriformes,Squatiniformes)),Echinorhinidae,Protospinacidae,Hexanchiformes)),Orectolobiformes),Heterodontiformes),Synechodontiformes))))

**HEI[31]**

(Hybodontiformes,(( Rajiformes^1^,(( Rhinobatid-like^2^,Myliobatiformes),Torpediniformes), Pristids^3^,Archaeobatidae,Sclerorhynchidae),(((((Squaliformes,Squatiniformes),Pristiophoriformes),Echinorhinidae,Protospinacidae,Hexanchiformes),((((Lamniformes,Carcharhiniformes),Orectolobiformes),Heterodontiformes),Synechodontiformes)))))


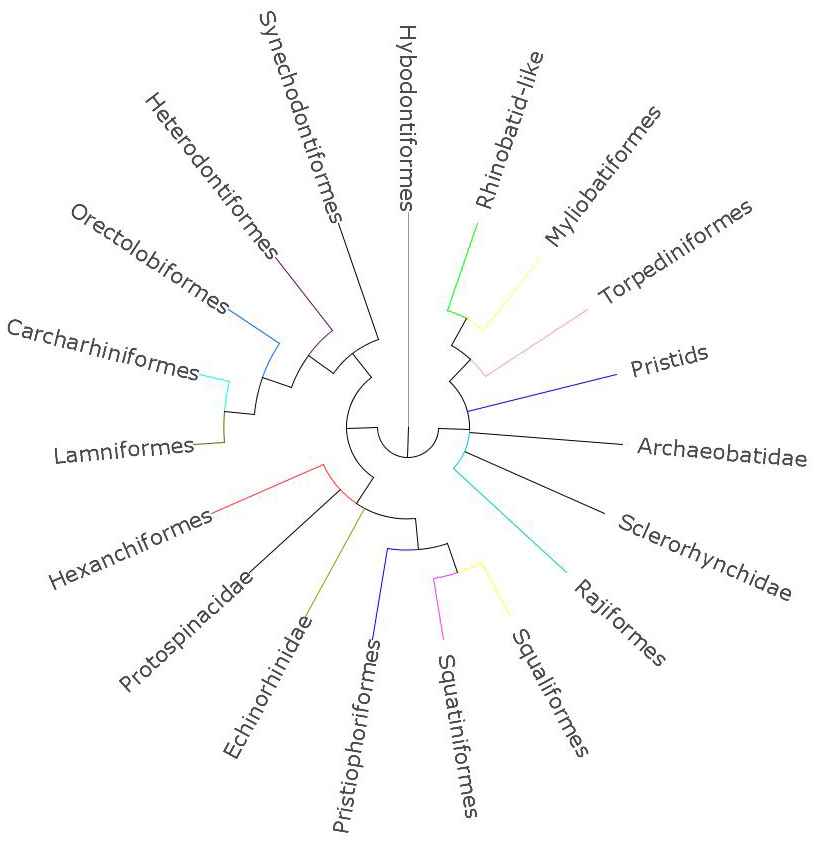


**HUM[32]** (Hybodontiformes,((( Rajiformes^1^,Myliobatiformes), Pristids^3^,Archaeobatidae,Sclerorhynchidae,Torpediniformes, Rhinobatid-like^2^),(((((((Pristiophoriformes,Squatiniformes),Squaliformes),Heterodontiformes),(Lamniformes,Carcharhiniformes),Orectolobiformes),Echinorhinidae,Protospinacidae,Hexanchiformes),Synechodontiformes))))

**MAI[33]** (Hybodontiformes,(( Rajiformes^1^,Archaeobatidae,Sclerorhynchidae,Torpediniformes, Rhinobatid-like^2^,(Myliobatiformes, Pristids^3^)),(((((Squaliformes,Squatiniformes),Pristiophoriformes),Echinorhinidae,Protospinacidae,Hexanchiformes),((((Lamniformes,Carcharhiniformes),Orectolobiformes),Heterodontiformes),Synechodontiformes)))))

**MAW[34]**

(Hybodontiformes,((Archaeobatidae,Sclerorhynchidae, Pristids^3^,((Myliobatiformes, Rhinobatid-like^2^),Torpediniformes),Rajiformes^1^),((Synechodontiformes,((((Squaliformes,Squatiniformes),Pristiophoriformes),Echinorhinidae,Protospinacidae,Hexanchiformes),Heterodontiformes),((Lamniformes,Orectolobiformes),Carcharhiniformes)))))

**MAW-m[34,20]**

(Hybodontiformes,((Archaeobatidae,Sclerorhynchidae, Pristids^3^,((Myliobatiformes,),Torpediniformes),Rajiformes^1^),((((Squaliformes,(Squatiniformes,Pristiophoriformes)),Echinorhinidae,Protospinacidae,Hexanchiformes),(((Lamniformes,(Orectolobiformes,Carcharhiniformes)),Heterodontiformes),Synechodontiformes)))))

**NAY[31]**

(Hybodontiformes,(( Rajiformes^1^,Torpediniformes,Myliobatiformes, Rhinobatid-like^2^, Pristids^3^,Archaeobatidae,Sclerorhynchidae),(((Squaliformes,((Squatiniformes,Pristiophoriformes),Echinorhinidae)),Hexanchiformes,Protospinacidae),((((Lamniformes,Carcharhiniformes),Orectolobiformes),Heterodontiformes),Synechodontiformes))))

^1^ excluding Rhynchobatidae, Rhinidae, Rhinobatidae, Platyrhinidae, Parapalaeobatidae, Hypsobatidae, Pristidae and relatives; ^2^ including Rhynchobatidae, Rhinidae, Rhinobatidae, Platyrhinidae, Parapalaeobatidae, Hypsobatidae and relatives; ^3^ including Pristidae and relative

**References**

1. Goto T (2001) Comparative anatomy, phylogeny and cladistic classification order Orectolobiformes (Chondrichthyes: Elasmobranchii). Memoirs of the Graduate School of Fisheries Sciences, Hokkaido University 28(1): 1-100.

2. Naylor GJP, Ryburn JA, Fedrigo O, Lopez JA (2005) Phylogenetic relationships among the major lineages of modern elasmobranchs. In: WC Hamlet (Ed.) Reproductive biology and phylogeny of Chondrichthyes: Sharks, batoids and chimaeras. Science Publishers, Enfield, NH, USA: 1-25.

3. Naylor GJP, Martin AP, Mattison EG, Brown WM (1997) Interrelationships of lamniform sharks: Testing phylogenetic hypotheses with sequence data. In: TD Kocher & CA Stepien (Eds). Molecular systematics of fishes, Academic Press, pp. 199-218.

4. Compagno LJV (1977) Phyletic relationships of living sharks and rays. Am Zool 17(2): 303-322.

5. Iglesias SP, Lecointre G, Sellos DY (2005) Extensive paraphylies within sharks of the order Carcharhiniformes inferred from nuclear and mitochondrial genes. Mol Phylogenet Evol 34(2005): 569-583.

6. Human BA (2007) A taxonomic revision of the catshark genus *Haploblepharus* Garman 1913 (Chondrichthyes: Carcharhiniformes: Scyliorhinidae). Zootaxa 1451: 1-40.

7. Shirai S (1996) Phylogenetic interrelationships of neoselachians (Chondrichthyes, Euselachii). In: MLJ Stiassny, LR Parenti & GD Johnson (Eds). Interrelationships of fishes. Academic Press, San Diego, London, pp. 9-34.

8. Adnet S, Cappetta H (2001) A palaeontological and phylogenetical analysis of squaliform sharks (Chondrichthyes: Squaliformes) based on dental characters. Lethaia 34: 234-248.

9. Straube N, Iglesias SP, Sellos DY, Kriwet J, Schliewen UK (2010) Molecular phylogeny and node time estimation of bioluminescent Lantern Sharks (Elasmobranchii: Etmopteridae). Mol Phylogenet Evol 56: 905-917.

10. Cappetta H (1987) Mesozoic and Cenozoic Elasmobranchii, Chondrichthyes II. In: H-P Schultze (Ed.) Handbook of palaeoichthyology Vol. 3B (pp.1-193). Stuttgart: Gustav Fischer Verlag.

11. Cappetta H (2012) Chondrichthyes (Mesozoic and Cenozoic Elasmobranchii: Teeth). In: H-P Schultze (Ed.) Handbook of palaeoichthyology, Vol. 3e. München: Verlag F. Pfeil, in press.

12. McEachran JD, Aschliman N (2004) Phylogeny of batoidea. In: Carrier JC, Musick JA, Heithaus MR, (Eds). Biology of sharks and their relatives. Boca Raton: CRC Press. p 79–114.

13. Lovejoy NR (1996) Systematics of myliobatoid elasmobranchs: with emphasis on the phylogeny and historical biogeography of neotropical freshwater stingrays (Potamotrygonidae: Rajiformes). Zool J Linn Soc-Lond 117: 207-257.

14. Compagno LJV (1999) Checklist of living elasmobranches, p.471-498. In: WC Hamlett, (Ed.) Sharks, skates and rays: the biology of elasmobranchs fishes, Johns Hopkins University Press. Maryland, 515 pp.

15. Aschliman NC, Nishida M, Miya M, Inoue JG, Rosana KM, Naylor GJP (2012) Body plan convergence in the evolution of skates and rays (Chondrichthyes: Batoidea). Mol Phylogenet Evol 63(1): 28-42. (doi:doi: 10.1016/j.ympev.2011.12.012).

16. Vélez-Zuazo X, Agnarsson I (2011) Shark tales: A molecular species-level phylogeny of sharks (Selachimorpha, Chondrichthyes). Mol Phylogenet Evol 58(2): 207-217.

17. Reif W-E (1973) Morphologie und Ultrastuktur des Hai-"Schmelzes". Zool Scr 2: 231-250.

18. Reif W-E (1977) Tooth enameloid as a taxonomic criterion: 1. A new euselachian shark from the Rhaeic-Liassic boundary. Neues Jahrb Geol P-M 1977: 565-576.

19. Ginter M, Hampe O, Duffin CJ (2010) Chondrichthyes Paleozoic Elasmobranchii: Teeth. In: H-P Schultze (Ed.) Handbook of palaeoichthyology, Vol. 3d. München: Verlag F. Pfeil, pp. 168.

20. Burrow CJ, Hovestadt DC, Hovestadt-Euler M, Turner S, Young G (2008) New information on the Devonian shark *Mcmurdodus*, based on material from western Queesland, Australia. Acta Geol Pol 58(2): 155-163.

21. Adnet S, Guinot G, Cappetta H, Welcomme J-L (2012) Oldest evidence of bramble sharks (Elasmobranchii, Echinorhinidae) in the Lower Cretaceous of southeast France and the evolutionary history of orbitostylic sharks. Cretaceous Res 35: 81-87. (doi: 10.1016/j.cretres.2011.11.021).

22. Cuny G, Martin M, Rauscher R, Mazin J-M (1998) A new neoselachian shark from the Upper Triasic of Grozon (Jura, France). Geol Mag 135(5): 657-668.

23. Cuny G, Benton MJ (1999) Early radiation of the Neoselachian sharks in Western Europe. Geobios 32: 193-204.

24. Duffin CJ (1980) A new euselachian shark from the Upper Triassic of Germany. Neues Jahrbuch Geol P-M 1980(1): 1-16.

25. Guinot G, Underwood CJ, Cappetta H, Ward DJ (2012) Squatiniformes (Chondrichthyes, Neoselachii) from the Late Cretaceous of southern England and Northern France with redescription of the holotype of *S. cranei* WOODWARD, 1888 Palaeontology, in press.

26. Douady CJ, Dosay M, Shivji MS, Stanhope MJ (2003) Molecular phylogenetic evidence refuting the hypothesis of Batoidea (rays and skates) as derived sharks. Mol Phylogenet Evol 26: 215-221.

27. Heinicke MP, Naylor GJP, Hedges SB (2009) Cartilaginous fishes (Chondrichthyes). In: Hedges, SB and Kumar, S (Eds), The timetree of Life, 320-327 pp. Oxford University Press.

28. Human BA, Owen EP, Compagno LJV, Harley EH (2006) Testing morphologically based phylogenetic theories within the cartilaginous fishes with molecular data, with special reference to the catshark family (Chondrichthyes; Scyliorhinidae) and the interrelationships within them. Mol Phylogenet Evol 39(2): 384-391.

29. Maisey JG, Naylor GJP, Ward DJ (2004) Mesozoic elasmobranches, neoselachian phylogeny and the rise of modern elasmobranch diversity. In: Arratia G & Tintori A (Eds.), Mesozoic fishes 3 - Systematics, palaeoenvironments and biodiversity. Verlag Dr. Friedrich Pfeil, München, pp. 17-56.

30. Mallatt J, Winchell CJ (2007) Ribosomal RNA genes and deuterostome phylogeny revisited: More cyclostomes, elasmobranchs, reptiles, and a brittle star. Mol Phylogenet Evol 43(3): 1005-1022.

31. Naylor GJP, Caira JN, Jensen K, Rosana KAM, Straube N, et al. (2012) Elasmobranch Phylogeny: A mitochondrial estimate based on 595 species. In: Carrier JC, Musick JA, Heithaus MR, editors. The Biology of Sharks and Their Relatives. 2nd Edition ed: CRC Press, Taylor & Francis Group. pp. 31-56.
