# Supplementary material for: An Analytical Approach for Estimating Fossil Record and Diversification Events in Sharks, Skates and Rays
Source: PLoS One. 2012 Sep 5;7(9):e44632. doi: 10.1371/journal.pone.0044632 (PMC3434181; doi:10.1371/journal.pone.0044632)
Supplement: File S2 — Stratigraphic framework used in the analyses. (DOC) [file pone.0044632.s005.doc]

**Supporting Information File S2**

Stratigraphic framework used in the analyses

Actual 0 0

Gelasian -1.81 -2.59

Piacenzian -2.59 -3.6

Zanclean -3.6 -5.33

Messinian -5.33 -7.25

Tortonian -7.25 -11.61

Serravallian -11.61 -13.65

Langhian -13.65 -15.97

Burdigalian -15.97 -20.43

Aquitanian -20.43 -23.03

Chattian -23.03 -28.4

Rupelian -28.49 -33.9

Priabonian -33.9 -37.2

Bartonian -37.2 -40.4

Lutetian -40.4 -48.6

Ypresian -48.6 -55.8

Thanetian -55.8 -58.7

Selandian -58.7 -61.7

Danian -61.7 -65.5

Maastrichtian -65.5 -70.6

Campanian -70.6 -83.5

Santonian -83.5 -85.8

Coniacian -85.8 -89.3

Turonian -89.3 -93.5

Cenomanian -93.5 -99.6

Albian -99.6 -112.0

Aptian -112.0 -125

Barremian -125 -130

Hauterivian -130 -136.4

Valanginian -136.4 -140.2

Berriasian -140.2 -145.5

Tithonian -145.5 -150.8

Kimmeridgian -150.8 -155.7

Oxfordian -155.7 -161.2

Callovian -161.2 -164.7

Bathonian -164.7 -167.7

Bajocian -167.7 -171.6

Aalenian -171.6 -175.6

Toarcian -175.6 -183

Pliensbachian -183 -189.6

Sinemurian -189.6 -196.5

Hettangian -196.5 -199.6

Rhaetian -199.6 -203.6

Norian -203.6 -216.5

Carnian -216.5 -228

Ladinian -228 -237

Anisian -237 -245

Olenekian -245 -249.7

Induan -249.7 -251

L-Pliocene -1.81 -3.6

E-Pliocene -3.6 -5.33

L-Miocene -5.33 -11.61

M-Miocene -11.61 -15.97

E-Miocene -15.97 -23.03

L-Oligocene -23.03 -28.4

E-Oligocene -28.4 -33.9

L-Eocene -33.9 -37.2

M-Eocene -37.2 -48.6

E-Eocene -48.6 -55.8

L-Paleocene -55.8 -58.7

M-Paleocene -58.7 -61.7

E-Paleocene -61.7 -65.5

L-Cretaceous -65.5 -99.6

E-Cretaceous -99.6 -145.5

L-Jurassic -145.5 -161.2

M-Jurassic -161.2 -175.6

E-Jurassic -175.6 -199.6

L-Triassic -199.6 -228

M-Triassic -228 -245

E-Triassic -245 -251

Permian -251 -299

Pleistocene 0 -1.81

Pliocene -1.81 -5.33

Miocene -5.33 -23.03

Oligocene -23.03 -33.9

Eocene -33.9 -55.8

Paleocene -55.8 -65.5

Cretaceous -65.5 -145.5

Jurassic -145.5 -199.6

Triassic -199.6 -251

Neogene 0 -23.3

Paleogene -23.3 -65.5

Neocomien -145.5 -130

Lias -175.6 -199.6

Tithonian2 -145.5 -150.8

Scynien -245 -251

Cisuralien -299 -270
