# Supplementary material for: An Analytical Approach for Estimating Fossil Record and Diversification Events in Sharks, Skates and Rays
Source: PLoS One. 2012 Sep 5;7(9):e44632. doi: 10.1371/journal.pone.0044632 (PMC3434181; doi:10.1371/journal.pone.0044632)
Supplement: File S3 — Observed and computed stratigraphic ranges for genera, families and orders. (DOC) [file pone.0044632.s006.doc]

**Supporting Information File S3**

Observed and computed stratigraphic ranges for genera, families and orders

| **GENERA** |  |  |  |  |  |  |  |  |  |  |  |  |  |
| --- | --- | --- | --- | --- | --- | --- | --- | --- | --- | --- | --- | --- | --- |
|  |  | **observed** |  | **CBM** | | | | | **DDBM** | | | | |
|  | **FAD** |  | **LAD** | **FAD** | |  | **LAD** | | **FAD** | |  | **LAD** | |
| *Abdounia* | **65,5** | to | **28,5** | 65,5 |  | to | 28,5 |  | 65,5 |  | to | 28,5 |  |
| *Acanthoscyllium* | **85,8** | to | **83,5** | 196,5 | 125,0 | to | 83,5 |  | 93,5 |  | to | 83,5 |  |
| *Acrodus* | **251,0** | to | **83,5** | 251,0 |  | to | 83,5 |  | 251,0 |  | to | 83,5 |  |
| *Acrolamna* | **112,0** | to | **70,6** | 140,2 | 125,0 | to | 70,6 |  | 112,0 |  | to | 70,6 |  |
| *Acrorhizodus* | **125,0** | to | **112,0** | 251,0 |  | to | 112,0 |  | 175,6 |  | to | 112,0 |  |
| *Acrosqualiolus* | **48,6** | to | **40,4** | 83,5 |  | to | 40,4 |  | 70,6 |  | to | 40,4 |  |
| *Aculeola* | **0,0** | to | **0,0** | 83,5 | 0,0 | to | 0,0 |  | 83,5 | 0,0 | to | 0,0 |  |
| *Aegyptobatus* | **99,6** | to | **93,5** | 270,0 |  | to | 93,5 |  | 99,6 |  | to | 93,5 |  |
| *Aetobatus* | **58,7** | to | **0,0** | 83,5 |  | to | 0,0 |  | 70,6 |  | to | 0,0 |  |
| *Aetomylaeus* | **16,0** | to | **0,0** | 83,5 |  | to | 0,0 |  | 70,6 |  | to | 0,0 |  |
| *Aetoplatea* | **0,0** | to | **0,0** | 58,7 |  | to | 0,0 |  | 40,4 |  | to | 0,0 |  |
| *Agaleus* | **196,5** | to | **183,0** | 196,5 |  | to | 183,0 |  | 245,0 | 196,5 | to | 183,0 |  |
| *Aktaua* | **48,6** | to | **37,2** | 83,5 |  | to | 37,2 |  | 70,6 |  | to | 37,2 |  |
| *Almascyllium* | **99,6** | to | **83,5** | 125,0 |  | to | 83,5 |  | 99,6 |  | to | 83,5 |  |
| *Alopias* | **55,8** | to | **0,0** | 136,4 | 99,6 | to | 0,0 |  | 65,5 |  | to | 0,0 |  |
| *Amblyraja* | **0,0** | to | **0,0** | 99,6 |  | to | 0,0 |  | 93,5 |  | to | 0,0 |  |
| *Anacanthobatis* | **0,0** | to | **0,0** | 99,6 |  | to | 0,0 |  | 99,6 |  | to | 0,0 |  |
| *Angolabatis* | **83,5** | to | **65,5** | 83,5 |  | to | 65,5 |  | 83,5 |  | to | 65,5 |  |
| *Angoumeius* | **48,6** | to | **37,2** | 83,5 |  | to | 37,2 |  | 70,6 |  | to | 37,2 |  |
| *Ankistrorhynchus* | **85,8** | to | **65,5** | 130,0 |  | to | 65,5 |  | 93,5 |  | to | 65,5 |  |
| *Annea* | **183,0** | to | **167,7** | 196,5 | 183,0 | to | 167,7 |  | 183,0 |  | to | 167,7 |  |
| *Anomotodon* | **125,0** | to | **28,5** | 130,0 | 125,0 | to | 28,5 |  | 125,0 |  | to | 28,5 |  |
| *Anotodus* | **20,4** | to | **3,6** | 99,6 |  | to | 3,6 |  | 65,5 |  | to | 3,6 |  |
| *Anoxypristis* | **48,6** | to | **0,0** | 183,0 | 136,4 | to | 0,0 |  | 48,6 |  | to | 0,0 |  |
| *Apocopodon* | **58,7** | to | **55,8** | 83,5 |  | to | 55,8 |  | 70,6 |  | to | 55,8 |  |
| *Apristurus* | **48,6** | to | **0,0** | 48,6 |  | to | 0,0 |  | 70,6 |  | to | 0,0 |  |
| *Aptychotrema* | **99,6** | to | **0,0** | 183,0 | 99,6 | to | 0,0 |  | 183,0 | 175,0 | to | 0,0 |  |
| *Araloselachus* | **37,2** | to | **13,7** | 136,4 | 125,0 | to | 13,7 |  | 125,0 | 37,2 | to | 13,7 |  |
| *Archaeolamna* | **112,0** | to | **65,5** | 125,0 |  | to | 65,5 |  | 112,0 |  | to | 65,5 |  |
| *Archaeomanta* | **65,5** | to | **40,4** | 136,4 | 99,6 | to | 40,4 |  | 99,6 | 83,5 | to | 40,4 |  |
| *Archaeotriakis* | **85,8** | to | **70,6** | 99,6 |  | to | 70,6 |  | 93,5 |  | to | 70,6 |  |
| *Archingeayia* | **0,0** | to | **0,0** | 183,0 |  | to | 0,0 |  | 183,0 |  | to | 0,0 |  |
| *Arechia* | **55,8** | to | **40,4** | 136,4 |  | to | 40,4 |  | 93,5 |  | to | 40,4 |  |
| *Arhynchobatis* | **0,0** | to | **0,0** | 0,0 |  | to | 0,0 |  | 0,0 |  | to | 0,0 |  |
| *Asteracanthus* | **245,0** | to | **99,6** | 251,0 |  | to | 99,6 |  | 245,0 |  | to | 99,6 |  |
| *Asterodermus* | **150,8** | to | **145,5** | 183,0 |  | to | 145,5 |  | 150,8 |  | to | 145,5 |  |
| *Asterotrygon* | **55,8** | to | **48,6** | 136,4 |  | to | 48,6 |  | 93,5 |  | to | 48,6 |  |
| *Asymbolus* | **0,0** | to | **0,0** | 89,3 | 0,0 | to | 0,0 |  | 70,6 | 0,0 | to | 0,0 |  |
| *Ataktobatis* | **70,6** | to | **65,5** | 183,0 |  | to | 65,5 |  | 175,6 |  | to | 65,5 |  |
| *Atelomycterus* | **0,0** | to | **0,0** | 140,2 | 0,0 | to | 0,0 |  | 164,7 | 0,0 | to | 0,0 |  |
| *Atlanticopristis* | **99,6** | to | **93,5** | 130,0 |  | to | 93,5 |  | 99,6 |  | to | 93,5 |  |
| *Atlantoraja* | **0,0** | to | **0,0** | 0,0 |  | to | 0,0 |  | 0,0 |  | to | 0,0 |  |
| *Aturobatis* | **55,8** | to | **33,9** | 136,4 |  | to | 33,9 |  | 93,5 |  | to | 33,9 |  |
| *Aulohalaelurus* | **0,0** | to | **0,0** | 167,7 | 0,0 | to | 0,0 |  | 164,7 | 0,0 | to | 0,0 |  |
| *Baharipristis* | **99,6** | to | **93,5** | 130,0 |  | to | 93,5 |  | 125,0 |  | to | 93,5 |  |
| *Bahariyodon* | **99,6** | to | **93,5** | 251,0 |  | to | 93,5 |  | 216,5 |  | to | 93,5 |  |
| *Bathyraja* | **0,0** | to | **0,0** | 0,0 |  | to | 0,0 |  | 0,0 |  | to | 0,0 |  |
| *Bavariscyllium* | **155,7** | to | **145,5** | 196,5 | 167,7 | to | 145,5 |  | 164,7 |  | to | 145,5 |  |
| *Bdellodus* | **183,0** | to | **175,6** | 251,0 |  | to | 175,6 |  | 183,0 |  | to | 175,6 |  |
| *Belemnobatis* | **167,7** | to | **125,0** | 167,7 |  | to | 125,0 |  | 175,6 |  | to | 125,0 |  |
| *Benthobatis* | **0,0** | to | **0,0** | 58,7 |  | to | 0,0 |  | 0,0 |  | to | 0,0 |  |
| *Biropristis* | **70,6** | to | **65,5** | 130,0 |  | to | 65,5 |  | 125,0 |  | to | 65,5 |  |
| *Borealotodus* | **37,2** | to | **33,9** | 48,6 | 37,2 | to | 33,9 |  | 125,0 |  | to | 33,9 |  |
| *Borodinopristis* | **85,8** | to | **70,6** | 130,0 |  | to | 70,6 |  | 93,5 |  | to | 70,6 |  |
| *Brachaelurus* | **130,0** | to | **0,0** | 136,4 | 130,0 | to | 0,0 |  | 130,0 |  | to | 0,0 |  |
| *Brachycarcharias* | **65,5** | to | **40,4** | 136,4 | 125,0 | to | 40,4 |  | 125,0 | 93,5 | to | 40,4 |  |
| *Brachyrhizodus* | **83,5** | to | **65,5** | 83,5 |  | to | 65,5 |  | 83,5 |  | to | 65,5 |  |
| *Breviacanthus* | **167,7** | to | **164,7** | 270,0 |  | to | 164,7 |  | 237,0 |  | to | 164,7 |  |
| *Breviraja* | **0,0** | to | **0,0** | 99,6 |  | to | 0,0 |  | 93,5 |  | to | 0,0 |  |
| *Brochiraja* | **0,0** | to | **0,0** | 0,0 |  | to | 0,0 |  | 0,0 |  | to | 0,0 |  |
| *Burnhamia* | **58,7** | to | **40,4** | 270,0 | 136,0 | to | 40,4 |  | 70,6 |  | to | 40,4 |  |
| *Bythaelurus* | **0,0** | to | **0,0** | 89,3 | 0,0 | to | 0,0 |  | 70,6 | 0,0 | to | 0,0 |  |
| *Cantioscyllium* | **130,0** | to | **65,5** | 130,0 |  | to | 65,5 |  | 136,4 | 130,0 | to | 65,5 |  |
| *Carcharhinus* | **48,6** | to | **0,0** | 55,8 | 48,6 | to | 0,0 |  | 48,6 |  | to | 0,0 |  |
| *Carcharias* | **125,0** | to | **0,0** | 140,2 | 125,0 | to | 0,0 |  | 125,0 |  | to | 0,0 |  |
| *Carchariolamna* | **23,0** | to | **5,3** | 65,5 | 48,6 | to | 5,3 |  | 23,0 |  | to | 5,3 |  |
| *Carcharodon* | **5,3** | to | **0,0** | 65,5 | 5,3 | to | 0,0 |  | 48,2 | 5,3 | to | 0,0 |  |
| *Carcharoides* | **33,9** | to | **11,6** | 37,2 | 33,9 | to | 11,6 |  | 33,9 |  | to | 11,6 |  |
| *Cardabiodon* | **99,6** | to | **89,3** | 136,4 | 125,0 | to | 89,3 |  | 125,0 | 112,0 | to | 89,3 |  |
| *Casieria* | **65,5** | to | **40,4** | 167,7 |  | to | 40,4 |  | 164,7 |  | to | 40,4 |  |
| *Cederstroemia* | **112,0** | to | **70,6** | 175,6 | 112,0 | to | 70,6 |  | 130,0 | 125,0 | to | 70,6 |  |
| *Celtipristis* | **130,0** | to | **125,0** | 130,0 |  | to | 125,0 |  | 130,0 |  | to | 125,0 |  |
| *Cenocarcharias* | **99,6** | to | **93,5** | 136,4 | 125,0 | to | 93,5 |  | 125,0 | 99,6 | to | 93,5 |  |
| *Centrophoroides* | **85,8** | to | **65,5** | 130,0 |  | to | 65,5 |  | 93,5 |  | to | 65,5 |  |
| *Centrophorus* | **70,6** | to | **0,0** | 99,6 |  | to | 0,0 |  | 70,6 |  | to | 0,0 |  |
| *Centroscyllium* | **0,0** | to | **0,0** | 83,5 | 48,6 | to | 0,0 |  | 83,5 | 0,0 | to | 0,0 |  |
| *Centroscymnus* | **83,5** | to | **0,0** | 93,5 | 85,8 | to | 0,0 |  | 83,5 |  | to | 0,0 |  |
| *Centroselachus* | **65,5** | to | **0,0** | 93,5 | 65,0 | to | 0,0 |  | 70,6 |  | to | 0,0 |  |
| *Centrosqualus* | **85,8** | to | **83,5** | 130,0 |  | to | 83,5 |  | 99,6 |  | to | 83,5 |  |
| *Cephaloscyllium* | **0,0** | to | **0,0** | 140,2 |  | to | 0,0 |  | 65,5 | 0,0 | to | 0,0 |  |
| *Cephalurus* | **0,0** | to | **0,0** | 167,7 | 0,0 | to | 0,0 |  | 164,7 | 0,0 | to | 0,0 |  |
| *Cetorhinus* | **48,6** | to | **0,0** | 136,4 | 65,5 | to | 0,0 |  | 55,8 | 48,6 | to | 0,0 |  |
| *Chaenogaleus* | **23,0** | to | **0,0** | 37,2 | 23,0 | to | 0,0 |  | 40,0 | 23,0 | to | 0,0 |  |
| *Chiloscyllium* | **125,0** | to | **0,0** | 125,0 |  | to | 0,0 |  | 125,0 |  | to | 0,0 |  |
| *Chlamydoselachus* | **85,8** | to | **0,0** | 196,5 |  | to | 0,0 |  | 183,0 |  | to | 0,0 |  |
| *Cirrhigaleus* | **0,0** | to | **0,0** | 130,0 |  | to | 0,0 |  | 93,5 |  | to | 0,0 |  |
| *Cirrhoscyllium* | **0,0** | to | **0,0** | 112,0 |  | to | 0,0 |  | 40,4 |  | to | 0,0 |  |
| *Columbusia* | **85,8** | to | **70,6** | 130,0 |  | to | 70,6 |  | 93,5 |  | to | 70,6 |  |
| *Corysodon* | **155,7** | to | **150,8** | 167,7 |  | to | 150,8 |  | 167,7 |  | to | 150,8 |  |
| *Cosmopolitodus* | **20,4** | to | **2,6** | 20,4 |  | to | 2,6 |  | 55,8 | 37,2 | to | 2,6 |  |
| *Coupatezia* | **83,5** | to | **33,9** | 136,4 |  | to | 33,9 |  | 93,5 |  | to | 33,9 |  |
| *Crassescyliorhinus* | **85,8** | to | **65,5** | 167,7 |  | to | 65,5 |  | 164,7 |  | to | 65,5 |  |
| *Crassinarke* | **0,0** | to | **0,0** | 0,0 |  | to | 0,0 |  | 0,0 |  | to | 0,0 |  |
| *Cretascyliorhinus* | **112,0** | to | **89,3** | 167,7 |  | to | 89,3 |  | 164,7 |  | to | 89,3 |  |
| *Cretascymnus* | **85,8** | to | **70,6** | 93,5 | 85,8 | to | 70,6 |  | 85,8 |  | to | 70,6 |  |
| *Cretodus* | **112,0** | to | **65,5** | 125,0 |  | to | 65,5 |  | 112,0 |  | to | 65,5 |  |
| *Cretolamna* | **112,0** | to | **33,9** | 125,0 |  | to | 33,9 |  | 112,0 |  | to | 33,9 |  |
| *Cretomanta* | **99,6** | to | **65,5** | 136,4 | 99,6 | to | 65,5 |  | 99,6 |  | to | 65,5 |  |
| *Cretorectolobus* | **136,4** | to | **70,6** | 175,6 |  | to | 70,6 |  | 136,4 | 125,0 | to | 70,6 |  |
| *Cretoxyrhina* | **112,0** | to | **83,5** | 125,0 |  | to | 83,5 |  | 112,0 |  | to | 83,5 |  |
| *Cristabatis* | **183,0** | to | **175,6** | 183,0 |  | to | 175,6 |  | 183,0 |  | to | 175,6 |  |
| *Ctenacis* | **0,0** | to | **0,0** | 167,7 |  | to | 0,0 |  | 164,7 |  | to | 0,0 |  |
| *Ctenopristis* | **89,3** | to | **65,5** | 130,0 |  | to | 65,5 |  | 93,5 |  | to | 65,5 |  |
| *Cyclobatis* | **99,6** | to | **93,5** | 99,6 |  | to | 93,5 |  | 99,6 |  | to | 93,5 |  |
| *Dactylobatus* | **0,0** | to | **0,0** | 99,6 |  | to | 0,0 |  | 93,5 |  | to | 0,0 |  |
| *Dalatias* | **48,6** | to | **0,0** | 83,5 | 58,7 | to | 0,0 |  | 70,6 | 48,6 | to | 0,0 |  |
| *Dallasiela* | **0,0** | to | **0,0** | 125,0 |  | to | 0,0 |  | 99,6 |  | to | 0,0 |  |
| *Dalpiazia* | **93,5** | to | **65,5** | 130,0 |  | to | 65,5 |  | 93,5 |  | to | 65,5 |  |
| *Danogaleus* | **65,5** | to | **61,7** | 65,5 |  | to | 61,7 |  | 65,5 |  | to | 61,7 |  |
| *Dasyatis* | **136,4** | to | **0,0** | 136,4 |  | to | 0,0 |  | 245,0 | 183,0 | to | 0,0 |  |
| *Dasyrhombodus* | **70,6** | to | **65,5** | 136,4 |  | to | 65,5 |  | 93,5 |  | to | 65,5 |  |
| *Deania* | **65,5** | to | **0,0** | 70,6 |  | to | 0,0 |  | 65,5 |  | to | 0,0 |  |
| *Delpitia* | **65,5** | to | **55,8** | 136,4 |  | to | 55,8 |  | 93,5 |  | to | 55,8 |  |
| *Delpitoscyllium* | **65,5** | to | **55,8** | 130,0 |  | to | 55,8 |  | 70,6 |  | to | 55,8 |  |
| *Dentiraja* | **0,0** | to | **0,0** | 99,6 |  | to | 0,0 |  | 93,5 |  | to | 0,0 |  |
| *Diplobatis* | **0,0** | to | **0,0** | 58,7 |  | to | 0,0 |  | 0,0 |  | to | 0,0 |  |
| *Diplolonchidion* | **228,0** | to | **216,5** | 251,0 |  | to | 216,5 |  | 228,0 |  | to | 216,5 |  |
| *Dipturus* | **13,7** | to | **0,0** | 270,0 | 183,0 | to | 0,0 |  | 93,5 |  | to | 0,0 |  |
| *Discopyge* | **0,0** | to | **0,0** | 58,7 |  | to | 0,0 |  | 0,0 |  | to | 0,0 |  |
| *Distobatus* | **99,6** | to | **93,5** | 251,0 |  | to | 93,5 |  | 203,6 |  | to | 93,5 |  |
| *Doliobatis* | **183,0** | to | **175,6** | 183,0 |  | to | 175,6 |  | 183,0 |  | to | 175,6 |  |
| *Doratodus* | **228,0** | to | **203,6** | 249,7 |  | to | 203,6 |  | 245,0 |  | to | 203,6 |  |
| *Dorsetoscyllium* | **167,7** | to | **164,7** | 183,0 |  | to | 164,7 |  | 171,6 |  | to | 164,7 |  |
| *Duwibatis* | **70,6** | to | **65,5** | 183,0 |  | to | 65,5 |  | 183,0 |  | to | 65,5 |  |
| *Dwardius* | **112,0** | to | **85,8** | 270,0 | 140,2 | to | 0,0 | 85,8 | 125,0 |  | to | 0,0 | 85,8 |
| *Echinorhinus* | **112,0** | to | **0,0** | 196,5 | 161,2 | to | 0,0 |  | 183,0 | 150,8 | to | 0,0 |  |
| *Egertonodus* | **145,5** | to | **140,2** | 251,0 |  | to | 140,2 |  | 164,7 |  | to | 140,2 |  |
| *Electrolux* | **0,0** | to | **0,0** | 0,0 |  | to | 0,0 |  | 0,0 |  | to | 0,0 |  |
| *Enantiobatis* | **99,6** | to | **93,5** | 136,4 |  | to | 93,5 |  | 99,6 |  | to | 93,5 |  |
| *Engaibatis* | **150,8** | to | **145,5** | 183,0 |  | to | 145,5 |  | 183,0 | 175,6 | to | 145,5 |  |
| *Engolismaia* | **99,6** | to | **93,5** | 183,0 |  | to | 93,5 |  | 183,0 |  | to | 93,5 |  |
| *Eoetmopterus* | **83,5** | to | **65,5** | 130,0 | 83,0 | to | 65,5 |  | 99,6 | 83,5 | to | 65,5 |  |
| *Eogaleus* | **55,8** | to | **48,6** | 65,5 |  | to | 48,6 |  | 61,7 |  | to | 48,6 |  |
| *Eometlaouia* | **58,7** | to | **48,6** | 112,0 |  | to | 48,6 |  | 70,6 | 58,7 | to | 48,6 |  |
| *Eomobula* | **55,8** | to | **40,4** | 58,7 |  | to | 40,4 |  | 55,8 |  | to | 40,4 |  |
| *Eoplinthicus* | **37,2** | to | **33,9** | 58,7 |  | to | 33,9 |  | 40,4 |  | to | 33,9 |  |
| *Eoptolamna* | **130,0** | to | **99,6** | 140,2 |  | to | 99,6 |  | 130,0 | 125,0 | to | 99,6 |  |
| *Eosqualiolus* | **48,6** | to | **40,4** | 270,0 | 93,5 | to | 40,4 |  | 70,6 |  | to | 40,4 |  |
| *Eostegostoma* | **65,5** | to | **37,2** | 136,4 | 130,0 | to | 37,2 |  | 70,6 | 65,5 | to | 37,2 |  |
| *Eostriatolamia* | **125,0** | to | **65,5** | 125,0 |  | to | 65,5 |  | 125,0 |  | to | 65,5 |  |
| *Eothrinax* | **70,6** | to | **40,4** | 85,8 |  | to | 40,4 |  | 70,6 |  | to | 40,4 |  |
| *Eotorpedo* | **65,5** | to | **48,6** | 65,5 |  | to | 48,6 |  | 183,0 |  | to | 48,6 |  |
| *Erguitaia* | **70,6** | to | **65,5** | 183,0 |  | to | 65,5 |  | 183,0 |  | to | 65,5 |  |
| *Eridacnis* | **0,0** | to | **0,0** | 167,7 | 0,0 | to | 0,0 |  | 164,7 | 0,0 | to | 0,0 |  |
| *Erythobatis* | **0,0** | to | **0,0** | 183,0 |  | to | 0,0 |  | 183,0 |  | to | 0,0 |  |
| *Etmopterus* | **48,6** | to | **0,0** | 270,0 | 83,0 | to | 0,0 |  | 65,5 |  | to | 0,0 |  |
| *Eucrossorhinus* | **0,0** | to | **0,0** | 112,0 |  | to | 0,0 |  | 70,6 |  | to | 0,0 |  |
| *Euprotomicroides* | **48,6** | to | **40,4** | 83,5 |  | to | 40,4 |  | 70,6 |  | to | 40,4 |  |
| *Euprotomicrus* | **0,0** | to | **0,0** | 48,6 |  | to | 0,0 |  | 40,4 |  | to | 0,0 |  |
| *Eusphyra* | **0,0** | to | **0,0** | 20,4 |  | to | 0,0 |  | 0,0 |  | to | 0,0 |  |
| *Ewingia* | **70,6** | to | **65,5** | 136,4 |  | to | 65,5 |  | 93,5 |  | to | 65,5 |  |
| *Eypea* | **167,7** | to | **164,7** | 167,7 |  | to | 164,7 |  | 183,0 | 167,7 | to | 164,7 |  |
| *Fenestraja* | **0,0** | to | **0,0** | 99,6 |  | to | 0,0 |  | 93,5 |  | to | 0,0 |  |
| *Figaro* | **0,0** | to | **0,0** | 167,7 |  | to | 0,0 |  | 164,7 |  | to | 0,0 |  |
| *Folipistrix* | **183,0** | to | **171,6** | 183,0 |  | to | 171,6 |  | 183,0 |  | to | 171,6 |  |
| *Foumtizia* | **65,5** | to | **40,4** | 167,7 |  | to | 40,4 |  | 164,7 |  | to | 40,4 |  |
| *Furgaleus* | **0,0** | to | **0,0** | 93,5 | 0,0 | to | 0,0 |  | 70,6 | 0,0 | to | 0,0 |  |
| *Galeocerdo* | **55,8** | to | **0,0** | 65,5 |  | to | 0,0 |  | 61,7 |  | to | 0,0 |  |
| *Galeocorax* | **93,5** | to | **65,5** | 136,4 | 93,5 | to | 65,5 |  | 99,6 |  | to | 65,5 |  |
| *Galeorhinus* | **99,6** | to | **0,0** | 99,6 |  | to | 0,0 |  | 164,7 |  | to | 0,0 |  |
| *Galeus* | **20,4** | to | **0,0** | 167,7 | 20,4 | to | 0,0 |  | 164,7 | 70,6 | to | 0,0 |  |
| *Ganntouria* | **70,6** | to | **65,5** | 130,0 |  | to | 65,5 |  | 70,6 |  | to | 65,5 |  |
| *Ganopristis* | **85,8** | to | **65,5** | 130,0 |  | to | 65,5 |  | 93,5 |  | to | 65,5 |  |
| *Garabatis* | **58,7** | to | **33,9** | 83,5 |  | to | 33,9 |  | 70,6 |  | to | 33,9 |  |
| *Gibbechinorhinus* | **70,6** | to | **65,5** | 112,0 |  | to | 65,5 |  | 70,6 |  | to | 65,5 |  |
| *Ginglymostoma* | **70,6** | to | **0,0** | 183,0 | 130,0 | to | 0,0 |  | 70,6 |  | to | 0,0 |  |
| *Glickmanodus* | **112,0** | to | **99,6** | 251,0 |  | to | 99,6 |  | 203,6 |  | to | 99,6 |  |
| *Glueckmanotodus* | **58,7** | to | **55,8** | 136,4 | 125,0 | to | 55,8 |  | 125,0 | 93,5 | to | 55,8 |  |
| *Glyphis* | **55,8** | to | **0,0** | 55,8 |  | to | 0,0 |  | 55,8 |  | to | 0,0 |  |
| *Gogolia* | **0,0** | to | **0,0** | 167,7 |  | to | 0,0 |  | 164,7 |  | to | 0,0 |  |
| *Gollum* | **0,0** | to | **0,0** | 0,0 |  | to | 0,0 |  | 0,0 |  | to | 0,0 |  |
| *Gomphogaleus* | **55,8** | to | **48,6** | 99,6 |  | to | 48,6 |  | 93,5 | 70,6 | to | 48,6 |  |
| *Grozonodon* | **216,5** | to | **203,6** | 270,0 |  | to | 203,6 |  | 245,0 |  | to | 203,6 |  |
| *Gurgesiella* | **0,0** | to | **0,0** | 99,6 |  | to | 0,0 |  | 93,5 |  | to | 0,0 |  |
| *Gymnura* | **58,7** | to | **0,0** | 99,6 |  | to | 0,0 |  | 93,0 |  | to | 0,0 |  |
| *Halaelurus* | **0,0** | to | **0,0** | 167,7 | 0,0 | to | 0,0 |  | 70,6 | 0,0 | to | 0,0 |  |
| *Hamrabatis* | **99,6** | to | **65,5** | 183,0 |  | to | 65,5 |  | 183,0 |  | to | 65,5 |  |
| *Haploblepharus* | **0,0** | to | **0,0** | 89,3 | 0,0 | to | 0,0 |  | 70,6 | 0,0 | to | 0,0 |  |
| *Heliobatis* | **55,8** | to | **48,6** | 136,4 |  | to | 48,6 |  | 93,5 | 61,7 | to | 48,6 |  |
| *Heliotrygon* | **0,0** | to | **0,0** | 0,0 |  | to | 0,0 |  | 0,0 |  | to | 0,0 |  |
| *Hemigaleus* | **0,0** | to | **0,0** | 93,5 | 48,6 | to | 0,0 |  | 40,4 | 33,9 | to | 0,0 |  |
| *Hemipristis* | **37,2** | to | **0,0** | 37,2 |  | to | 0,0 |  | 70,6 | 65,5 | to | 0,0 |  |
| *Hemiscyllium* | **70,6** | to | **0,0** | 125,0 |  | to | 0,0 |  | 93,5 | 83,5 | to | 0,0 |  |
| *Hemitriakis* | **0,0** | to | **0,0** | 99,6 | 93,5 | to | 0,0 |  | 70,6 |  | to | 0,0 |  |
| *Heptranchias* | **83,5** | to | **0,0** | 145,5 |  | to | 0,0 |  | 83,5 |  | to | 0,0 |  |
| *Heterobatis* | **58,7** | to | **55,8** | 136,4 |  | to | 55,8 |  | 93,5 |  | to | 55,8 |  |
| *Heterodontus* | **175,6** | to | **0,0** | 270,0 | 183,0 | to | 0,0 |  | 175,6 |  | to | 0,0 |  |
| *Heteronarce* | **0,0** | to | **0,0** | 183,0 | 65,5 | to | 0,0 |  | 58,7 | 48,0 | to | 0,0 |  |
| *Heterophorcynus* | **167,7** | to | **164,7** | 167,7 |  | to | 164,7 |  | 171,6 |  | to | 164,7 |  |
| *Heteroptychodus* | **130,0** | to | **112,0** | 251,0 |  | to | 112,0 |  | 251,0 |  | to | 112,0 |  |
| *Heteroscyllium* | **0,0** | to | **0,0** | 130,0 |  | to | 0,0 |  | 65,5 |  | to | 0,0 |  |
| *Heteroscymnoides* | **0,0** | to | **0,0** | 83,5 |  | to | 0,0 |  | 70,6 |  | to | 0,0 |  |
| *Heterotorpedo* | **65,5** | to | **37,2** | 136,4 |  | to | 37,2 |  | 93,5 |  | to | 37,2 |  |
| *Hexanchus* | **145,5** | to | **0,0** | 196,5 |  | to | 0,0 |  | 183,0 | 150,8 | to | 0,0 |  |
| *Hexatrygon* | **48,6** | to | **0,0** | 136,4 | 58,7 | to | 0,0 |  | 99,6 | 58,7 | to | 0,0 |  |
| *Himantura* | **20,4** | to | **0,0** | 136,4 | 58,7 | to | 0,0 |  | 61,7 | 55,8 | to | 0,0 |  |
| *Hispidaspis* | **136,4** | to | **65,5** | 136,4 |  | to | 65,5 |  | 136,4 |  | to | 65,5 |  |
| *Hologinglymostoma* | **58,7** | to | **55,8** | 130,0 |  | to | 55,8 |  | 70,6 |  | to | 55,8 |  |
| *Holohalaelurus* | **0,0** | to | **0,0** | 167,7 | 0,0 | to | 0,0 |  | 164,7 | 0,0 | to | 0,0 |  |
| *Homalodontus* | **249,7** | to | **245,0** | 251,0 |  | to | 245,0 |  | 251,0 |  | to | 245,0 |  |
| *Hongeo* | **0,0** | to | **0,0** | 99,6 |  | to | 0,0 |  | 93,5 |  | to | 0,0 |  |
| *Hubeiodus* | **171,6** | to | **164,7** | 251,0 |  | to | 164,7 |  | 245,0 |  | to | 164,7 |  |
| *Hueneichthys* | **203,6** | to | **199,6** | 270,0 |  | to | 199,6 |  | 245,0 |  | to | 199,6 |  |
| *Hybodus* | **203,6** | to | **65,5** | 203,6 |  | to | 65,5 |  | 245,0 |  | to | 65,5 |  |
| *Hylaeobatis* | **130,0** | to | **125,0** | 251,0 |  | to | 125,0 |  | 216,5 |  | to | 125,0 |  |
| *Hypnos* | **0,0** | to | **0,0** | 65,5 |  | to | 0,0 |  | 58,7 |  | to | 0,0 |  |
| *Hypolophites* | **65,5** | to | **55,8** | 136,4 |  | to | 55,8 |  | 93,5 | 65,5 | to | 55,8 |  |
| *Hypolophodon* | **58,7** | to | **48,6** | 136,4 |  | to | 48,6 |  | 93,5 |  | to | 48,6 |  |
| *Hypotodus* | **55,8** | to | **37,2** | 136,4 | 125,0 | to | 37,2 |  | 125,0 | 93,5 | to | 37,2 |  |
| *Hypsobatis* | **83,5** | to | **65,5** | 183,0 |  | to | 65,5 |  | 183,0 | 175,0 | to | 65,5 |  |
| *Iago* | **55,8** | to | **0,0** | 167,7 | 99,6 | to | 0,0 |  | 93,5 | 70,6 | to | 0,0 |  |
| *Iansan* | **112,0** | to | **99,6** | 183,0 |  | to | 99,6 |  | 175,6 |  | to | 99,6 |  |
| *Iberotrygon* | **99,6** | to | **93,5** | 183,0 |  | to | 93,5 |  | 183,0 |  | to | 93,5 |  |
| *Igdabatis* | **83,5** | to | **65,5** | 83,5 |  | to | 65,5 |  | 83,5 |  | to | 65,5 |  |
| *Ikamauius* | **37,2** | to | **0,0** | 112,0 |  | to | 0,0 |  | 37,2 |  | to | 0,0 |  |
| *Insentiraja* | **0,0** | to | **0,0** | 99,6 |  | to | 0,0 |  | 93,5 |  | to | 0,0 |  |
| *Irolita* | **0,0** | to | **0,0** | 0,0 |  | to | 0,0 |  | 0,0 |  | to | 0,0 |  |
| *Isanodus* | **145,5** | to | **112,0** | 251,0 |  | to | 112,0 |  | 216,5 |  | to | 112,0 |  |
| *Ischyrhiza* | **93,5** | to | **65,5** | 130,0 |  | to | 65,5 |  | 93,5 |  | to | 65,5 |  |
| *Ishaquia* | **65,5** | to | **61,7** | 136,4 |  | to | 61,7 |  | 93,5 | 65,5 | to | 61,7 |  |
| *Isidobatus* | **99,6** | to | **93,5** | 183,0 |  | to | 93,5 |  | 183,0 | 175,6 | to | 93,5 |  |
| *Isistius* | **58,7** | to | **0,0** | 83,5 |  | to | 0,0 |  | 70,6 |  | to | 0,0 |  |
| *Isogomphodon* | **23,0** | to | **0,0** | 55,8 | 48,6 | to | 0,0 |  | 48,6 | 23,0 | to | 0,0 |  |
| *Isurolamna* | **65,5** | to | **23,0** | 65,5 |  | to | 23,0 |  | 125,0 | 112,0 | to | 23,0 |  |
| *Isurus* | **33,9** | to | **0,0** | 65,5 |  | to | 0,0 |  | 33,9 |  | to | 0,0 |  |
| *Ixobatis* | **70,6** | to | **65,5** | 83,5 |  | to | 65,5 |  | 70,6 |  | to | 65,5 |  |
| *Jacquhermania* | **55,8** | to | **40,4** | 58,7 |  | to | 40,4 |  | 55,8 |  | to | 40,4 |  |
| *Jaekelotodus* | **65,5** | to | **33,9** | 136,4 | 125,0 | to | 33,9 |  | 125,0 | 93,5 | to | 33,9 |  |
| *Jiaodontus* | **161,2** | to | **155,7** | 251,0 |  | to | 155,7 |  | 216,5 |  | to | 155,7 |  |
| *Johnlongia* | **112,0** | to | **83,5** | 136,4 | 125,0 | to | 83,5 |  | 125,0 | 112,0 | to | 83,5 |  |
| *Jurobatos* | **183,0** | to | **175,6** | 183,0 |  | to | 175,6 |  | 183,0 |  | to | 175,6 |  |
| *Karaisurus* | **40,4** | to | **37,2** | 65,5 |  | to | 37,2 |  | 55,8 | 40,4 | to | 37,2 |  |
| *Khoratodus* | **125,0** | to | **112,0** | 203,6 |  | to | 112,0 |  | 164,7 |  | to | 112,0 |  |
| *Khouribgaleus* | **58,7** | to | **55,8** | 99,6 |  | to | 55,8 |  | 93,5 | 70,6 | to | 55,8 |  |
| *Kiestus* | **93,5** | to | **85,8** | 130,0 |  | to | 85,8 |  | 93,5 |  | to | 85,8 |  |
| *Kruckowlamna* | **5,3** | to | **3,6** | 65,5 |  | to | 3,6 |  | 61,7 |  | to | 3,6 |  |
| *Lamiopsis* | **0,0** | to | **0,0** | 55,8 |  | to | 0,0 |  | 0,0 |  | to | 0,0 |  |
| *Lamna* | **5,3** | to | **0,0** | 48,6 | 33,9 | to | 0,0 |  | 11,6 | 5,3 | to | 0,0 |  |
| *Leidybatis* | **58,7** | to | **40,4** | 83,5 |  | to | 40,4 |  | 70,6 |  | to | 40,4 |  |
| *Leiribatos* | **155,7** | to | **150,8** | 183,0 |  | to | 150,8 |  | 183,0 |  | to | 150,8 |  |
| *Leptacanthus* | **199,6** | to | **164,7** | 203,6 |  | to | 164,7 |  | 199,6 |  | to | 164,7 |  |
| *Leptocharias* | **0,0** | to | **0,0** | 99,6 |  | to | 0,0 |  | 93,5 | 70,6 | to | 0,0 |  |
| *Leptostyrax* | **112,0** | to | **99,6** | 140,2 |  | to | 99,6 |  | 125,0 |  | to | 99,6 |  |
| *Lethenia* | **33,9** | to | **23,0** | 65,5 |  | to | 23,0 |  | 55,8 | 37,2 | to | 23,0 |  |
| *Leucoraja* | **0,0** | to | **0,0** | 99,6 |  | to | 0,0 |  | 93,5 |  | to | 0,0 |  |
| *Libanopristis* | **99,6** | to | **93,5** | 130,0 |  | to | 93,5 |  | 99,6 |  | to | 93,5 |  |
| *Lissodus* | **251,0** | to | **99,6** | 251,0 |  | to | 99,6 |  | 251,0 |  | to | 99,6 |  |
| *Lonchidion* | **183,0** | to | **65,5** | 251,0 |  | to | 65,5 |  | 216,5 |  | to | 65,5 |  |
| *Lophobatis* | **55,8** | to | **48,6** | 83,5 |  | to | 48,6 |  | 70,6 |  | to | 48,6 |  |
| *Loxodon* | **0,0** | to | **0,0** | 55,8 | 23,0 | to | 0,0 |  | 48,6 | 0,0 | to | 0,0 |  |
| *Macrorhizodus* | **55,8** | to | **28,5** | 55,8 |  | to | 28,5 |  | 55,8 |  | to | 28,5 |  |
| *Macrourogaleus* | **150,8** | to | **145,5** | 167,7 |  | to | 145,5 |  | 164,7 |  | to | 145,5 |  |
| *Mafdetia* | **99,6** | to | **93,5** | 99,6 |  | to | 93,5 |  | 245,0 | 183,0 | to | 93,5 |  |
| *Malacoraja* | **48,6** | to | **0,0** | 99,6 |  | to | 0,0 |  | 93,5 |  | to | 0,0 |  |
| *Manta* | **5,3** | to | **0,0** | 58,7 |  | to | 0,0 |  | 40,4 |  | to | 0,0 |  |
| *Marckgrafia* | **0,0** | to | **0,0** | 130,0 |  | to | 93,5 |  | 99,6 |  | to | 93,5 |  |
| *Megachasma* | **28,4** | to | **0,0** | 99,6 |  | to | 0,0 |  | 65,5 |  | to | 0,0 |  |
| *Megascyliorhinus* | **55,8** | to | **0,0** | 167,7 |  | to | 0,0 |  | 164,7 |  | to | 0,0 |  |
| *Megasqualus* | **61,7** | to | **48,6** | 130,0 |  | to | 48,6 |  | 99,6 |  | to | 48,6 |  |
| *Mennerotodus* | **48,6** | to | **37,2** | 136,4 | 112,0 | to | 37,2 |  | 125,0 | 33,9 | to | 37,2 |  |
| *Merabatis* | **55,8** | to | **40,4** | 136,4 |  | to | 40,4 |  | 93,5 |  | to | 40,4 |  |
| *Meridiana* | **0,0** | to | **0,0** | 136,4 |  | to | 0,0 |  | 93,5 |  | to | 0,0 |  |
| *Meristodon* | **199,6** | to | **70,6** | 203,6 |  | to | 70,6 |  | 199,6 |  | to | 70,6 |  |
| *Meristodonoides* | **112,0** | to | **83,5** | 203,6 |  | to | 83,5 |  | 164,7 |  | to | 83,5 |  |
| *Mesiteia* | **99,6** | to | **93,5** | 125,0 |  | to | 93,5 |  | 99,6 |  | to | 93,5 |  |
| *Microbatis* | **58,7** | to | **55,8** | 183,0 |  | to | 55,8 |  | 183,0 | 175,0 | to | 55,8 |  |
| *Microetmopterus* | **70,6** | to | **65,5** | 83,5 |  | to | 65,5 |  | 70,6 |  | to | 65,5 |  |
| *Micropristis* | **99,6** | to | **70,6** | 130,0 |  | to | 70,6 |  | 99,6 |  | to | 70,6 |  |
| *Microscyliorhinus* | **58,7** | to | **48,6** | 167,7 |  | to | 48,6 |  | 164,7 |  | to | 48,6 |  |
| *Miroscyllium* | **16,0** | to | **0,0** | 83,5 | 48,6 | to | 0,0 |  | 65,5 | 16,0 | to | 0,0 |  |
| *Misrichthys* | **40,4** | to | **33,9** | 65,5 |  | to | 33,9 |  | 61,7 |  | to | 33,9 |  |
| *Mitsukurina* | **48,6** | to | **0,0** | 140,2 |  | to | 0,0 |  | 99,6 | 65,5 | to | 0,0 |  |
| *Mobula* | **23,0** | to | **0,0** | 58,7 |  | to | 0,0 |  | 40,4 |  | to | 0,0 |  |
| *Moerigaleus* | **37,2** | to | **33,9** | 37,2 |  | to | 33,9 |  | 37,2 |  | to | 33,9 |  |
| *Mollisquama* | **48,6** | to | **0,0** | 83,5 |  | to | 0,0 |  | 70,6 |  | to | 0,0 |  |
| *Mucrovenator* | **245,0** | to | **237,0** | 270,0 |  | to | 237,0 |  | 245,0 |  | to | 237,0 |  |
| *Mukdahanodus* | **145,5** | to | **125,0** | 251,0 |  | to | 125,0 |  | 251,0 |  | to | 125,0 |  |
| *Mustelus* | **58,7** | to | **0,0** | 99,6 | 93,5 | to | 0,0 |  | 70,6 |  | to | 0,0 |  |
| *Myledaphus* | **93,5** | to | **65,5** | 183,0 |  | to | 65,5 |  | 183,0 | 175,6 | to | 65,5 |  |
| *Myliobatis* | **65,5** | to | **0,0** | 83,5 |  | to | 0,0 |  | 70,6 |  | to | 0,0 |  |
| *Myliodasyatis* | **65,5** | to | **61,7** | 136,4 |  | to | 61,7 |  | 93,5 |  | to | 61,7 |  |
| *Nanocorax* | **99,6** | to | **70,6** | 112,0 |  | to | 70,6 |  | 99,6 |  | to | 70,6 |  |
| *Narcine* | **58,7** | to | **0,0** | 58,7 |  | to | 0,0 |  | 58,7 |  | to | 0,0 |  |
| *Narke* | **0,0** | to | **0,0** | 0,0 |  | to | 0,0 |  | 0,0 |  | to | 0,0 |  |
| *Nasolamia* | **5,3** | to | **0,0** | 55,8 | 48,5 | to | 0,0 |  | 5,3 |  | to | 0,0 |  |
| *Nebrius* | **70,6** | to | **0,0** | 130,0 |  | to | 0,0 |  | 70,6 |  | to | 0,0 |  |
| *Negaprion* | **20,4** | to | **0,0** | 65,5 |  | to | 0,0 |  | 61,7 |  | to | 0,0 |  |
| *Neoraja* | **0,0** | to | **0,0** | 99,6 |  | to | 0,0 |  | 93,5 |  | to | 0,0 |  |
| *Neotrygon* | **0,0** | to | **0,0** | 136,4 |  | to | 0,0 |  | 93,5 |  | to | 0,0 |  |
| *Notidanodon* | **140,2** | to | **55,8** | 196,5 |  | to | 55,8 |  | 183,0 |  | to | 55,8 |  |
| *Notidanoides* | **196,5** | to | **150,8** | 196,5 |  | to | 150,8 |  | 245,0 | 196,5 | to | 150,8 |  |
| *Notoraja* | **0,0** | to | **0,0** | 0,0 |  | to | 0,0 |  | 0,0 |  | to | 0,0 |  |
| *Notorhynchus* | **0,0** | to | **0,0** | 196,5 |  | to | 0,0 |  | 183,0 | 150,8 | to | 0,0 |  |
| *Odontaspis* | **83,5** | to | **0,0** | 136,4 | 125,0 | to | 0,0 |  | 125,0 | 93,5 | to | 0,0 |  |
| *Okamejei* | **0,0** | to | **0,0** | 99,6 |  | to | 0,0 |  | 93,5 |  | to | 0,0 |  |
| *Onchopristis* | **130,0** | to | **93,5** | 130,0 |  | to | 93,5 |  | 183,0 |  | to | 93,5 |  |
| *Onchosaurus* | **93,5** | to | **65,5** | 183,0 |  | to | 65,5 |  | 125,0 |  | to | 65,5 |  |
| *Orectoloboides* | **112,0** | to | **5,3** | 112,0 |  | to | 5,3 |  | 112,0 |  | to | 5,3 |  |
| *Orectolobus* | **48,6** | to | **0,0** | 112,0 |  | to | 0,0 |  | 70,6 |  | to | 0,0 |  |
| *Ornatoscyllium* | **167,7** | to | **112,0** | 183,0 |  | to | 112,0 |  | 171,6 |  | to | 112,0 |  |
| *Orpodon* | **61,7** | to | **58,7** | 136,4 | 125,0 | to | 58,7 |  | 125,0 | 93,5 | to | 58,7 |  |
| *Orthechinorhinus* | **55,8** | to | **40,4** | 112,0 |  | to | 40,4 |  | 65,5 |  | to | 40,4 |  |
| *Ostenoselache* | **196,5** | to | **189,6** | 251,0 |  | to | 189,6 |  | 251,0 |  | to | 189,6 |  |
| *OtodusC* | **55,8** | to | **23,0** | 65,5 |  | to | 23,0 |  | 55,8 |  | to | 23,0 |  |
| *OtodusM* | **20,4** | to | **2,6** | 65,5 |  | to | 2,6 |  | 48,6 |  | to | 2,6 |  |
| *OtodusO* | **65,5** | to | **48,6** | 136,4 | 65,5 | to | 48,6 |  | 125,0 | 112,0 | to | 48,6 |  |
| *Ouledia* | **58,7** | to | **37,2** | 58,7 |  | to | 37,2 |  | 58,7 |  | to | 37,2 |  |
| *Oxynotus* | **20,4** | to | **0,0** | 130,0 | 93,5 | to | 0,0 |  | 70,6 |  | to | 0,0 |  |
| *Pachygaleus* | **58,7** | to | **40,4** | 99,6 | 93,5 | to | 40,4 |  | 70,6 |  | to | 40,4 |  |
| *Pachyhexanchus* | **140,2** | to | **130,0** | 196,5 |  | to | 130,0 |  | 183,0 | 150,8 | to | 130,0 |  |
| *Pachyscyllium* | **58,7** | to | **3,6** | 65,5 |  | to | 3,6 |  | 58,7 |  | to | 3,6 |  |
| *Palaeobates* | **245,0** | to | **237,0** | 251,0 |  | to | 237,0 |  | 245,0 |  | to | 237,0 |  |
| *Palaeobrachaelurus* | **175,6** | to | **125,0** | 175,6 |  | to | 125,0 |  | 175,6 |  | to | 125,0 |  |
| *Palaeocarcharias* | **155,7** | to | **150,8** | 196,5 |  | to | 150,8 |  | 183,0 |  | to | 150,8 |  |
| *Palaeocarcharodon* | **65,5** | to | **55,8** | 125,0 |  | to | 55,8 |  | 99,6 |  | to | 55,8 |  |
| *Palaeogaleus* | **83,5** | to | **48,6** | 99,6 | 93,5 | to | 48,6 |  | 83,5 |  | to | 48,6 |  |
| *Palaeohypotodus* | **65,5** | to | **55,8** | 136,4 | 125,0 | to | 55,8 |  | 125,0 | 93,5 | to | 55,8 |  |
| *Palaeorectolobus* | **150,8** | to | **145,5** | 196,5 |  | to | 145,5 |  | 183,0 |  | to | 145,5 |  |
| *Palaeorhincodon* | **58,7** | to | **40,4** | 130,0 | 58,7 | to | 40,4 |  | 70,6 |  | to | 40,4 |  |
| *Palaeoscyllium* | **167,7** | to | **99,6** | 167,7 |  | to | 99,6 |  | 167,7 |  | to | 99,6 |  |
| *Palidiplospinax* | **196,5** | to | **175,6** | 270,0 |  | to | 175,6 |  | 237,0 |  | to | 175,6 |  |
| *Paracestracion* | **183,0** | to | **145,5** | 183,0 |  | to | 145,5 |  | 183,0 |  | to | 145,5 |  |
| *Paraechinorhinus* | **48,6** | to | **11,6** | 112,0 |  | to | 11,6 |  | 65,5 |  | to | 11,6 |  |
| *Paraetmopterus* | **48,6** | to | **40,4** | 83,5 |  | to | 40,4 |  | 65,5 |  | to | 40,4 |  |
| *Paragaleus* | **20,4** | to | **0,0** | 37,2 | 48,6 | to | 0,0 |  | 33,9 | 20,4 | to | 0,0 |  |
| *Paraginglymostoma* | **125,0** | to | **65,5** | 175,6 | 136,4 | to | 65,5 |  | 125,0 |  | to | 65,5 |  |
| *Paraheptranchias* | **20,4** | to | **13,7** | 145,0 | 83,5 | to | 13,7 |  | 48,6 | 20,4 | to | 13,7 |  |
| *Paraisurus* | **125,0** | to | **99,6** | 125,0 |  | to | 99,6 |  | 125,0 |  | to | 99,6 |  |
| *Paranomotodon* | **99,6** | to | **65,5** | 99,6 |  | to | 65,5 |  | 125,0 | 112,0 | to | 65,5 |  |
| *Paraorthacodus* | **155,7** | to | **55,8** | 196,5 |  | to | 55,8 |  | 183,0 |  | to | 55,8 |  |
| *Parapaleobates* | **0,0** | to | **0,0** | 183,0 |  | to | 0,0 |  | 183,0 | 175,6 | to | 0,0 |  |
| *Paraphorosoides* | **83,5** | to | **70,6** | 83,5 |  | to | 70,6 |  | 83,5 |  | to | 70,6 |  |
| *Pararhincodon* | **112,0** | to | **40,4** | 183,0 |  | to | 40,4 |  | 171,6 |  | to | 40,4 |  |
| *Parascyllium* | **48,6** | to | **0,0** | 112,0 |  | to | 0,0 |  | 48,6 |  | to | 0,0 |  |
| *Parasquatina* | **70,6** | to | **61,7** | 270,0 | 196,5 | to | 61,7 |  | 245,0 | 183,0 | to | 61,7 |  |
| *Paratriakis* | **93,5** | to | **70,6** | 99,6 | 93,5 | to | 70,6 |  | 93,5 |  | to | 70,6 |  |
| *Paratrygon* | **0,0** | to | **0,0** | 11,6 |  | to | 0,0 |  | 0,0 |  | to | 0,0 |  |
| *Paratrygonorrhina* | **83,5** | to | **65,5** | 183,0 |  | to | 65,5 |  | 183,0 | 175,6 | to | 65,5 |  |
| *Parmaturus* | **0,0** | to | **0,0** | 167,7 | 0,0 | to | 0,0 |  | 164,7 | 0,0 | to | 0,0 |  |
| *Parotodus* | **55,8** | to | **3,6** | 65,5 |  | to | 3,6 |  | 55,8 |  | to | 3,6 |  |
| *Parvodus* | **196,5** | to | **140,2** | 251,0 |  | to | 140,2 |  | 216,5 |  | to | 140,2 |  |
| *Pastinachus* | **20,4** | to | **0,0** | 136,4 |  | to | 0,0 |  | 93,5 | 61,7 | to | 0,0 |  |
| *Pavoraja* | **0,0** | to | **0,0** | 0,0 |  | to | 0,0 |  | 0,0 |  | to | 0,0 |  |
| *Pentanchus* | **0,0** | to | **0,0** | 167,7 | 0,0 | to | 0,0 |  | 0,0 |  | to | 0,0 |  |
| *Phorcynis* | **155,7** | to | **150,8** | 183,0 |  | to | 150,8 |  | 164,7 |  | to | 150,8 |  |
| *Phosphatobatis* | **89,3** | to | **65,5** | 183,0 |  | to | 65,5 |  | 183,0 |  | to | 65,5 |  |
| *Phosphatodon* | **65,5** | to | **61,7** | 136,4 |  | to | 61,7 |  | 93,5 |  | to | 61,7 |  |
| *Physogaleus* | **55,8** | to | **13,7** | 65,5 |  | to | 13,7 |  | 61,7 |  | to | 13,7 |  |
| *Planohybodus* | **164,7** | to | **155,7** | 203,6 |  | to | 155,7 |  | 164,7 |  | to | 155,7 |  |
| *Platypterix* | **130,0** | to | **125,0** | 183,0 |  | to | 125,0 |  | 183,0 | 175,6 | to | 125,0 |  |
| *Platyrhina* | **55,8** | to | **0,0** | 183,0 |  | to | 0,0 |  | 65,5 |  | to | 0,0 |  |
| *Platyrhinoidis* | **0,0** | to | **0,0** | 83,5 |  | to | 0,0 |  | 65,5 |  | to | 0,0 |  |
| *Platyrhizoscyllium* | **48,6** | to | **40,4** | 167,7 |  | to | 40,4 |  | 164,7 | 48,6 | to | 40,4 |  |
| *Plesiobatis* | **58,7** | to | **0,0** | 58,7 |  | to | 0,0 |  | 93,5 | 58,7 | to | 0,0 |  |
| *Plesiotrygon* | **0,0** | to | **0,0** | 11,6 |  | to | 0,0 |  | 0,0 |  | to | 0,0 |  |
| *Plicatopristis* | **89,3** | to | **65,5** | 130,0 |  | to | 65,5 |  | 93,5 |  | to | 65,5 |  |
| *Plicatoscyllium* | **83,5** | to | **65,5** | 83,5 |  | to | 65,5 |  | 83,5 |  | to | 65,5 |  |
| *Plinthicus* | **33,9** | to | **13,7** | 37,2 |  | to | 13,7 |  | 33,9 |  | to | 13,7 |  |
| *Pliotremata* | **0,0** | to | **0,0** | 37,2 |  | to | 0,0 |  | 0,0 |  | to | 0,0 |  |
| *Polyacrodus* | **245,0** | to | **70,6** | 245,0 |  | to | 70,6 |  | 245,0 |  | to | 70,6 |  |
| *Poroderma* | **0,0** | to | **0,0** | 65,5 |  | to | 0,0 |  | 55,8 |  | to | 0,0 |  |
| *Porodermoides* | **65,5** | to | **55,8** | 167,7 | 65,5 | to | 55,8 |  | 65,5 |  | to | 55,8 |  |
| *Pororhiza* | **112,0** | to | **99,6** | 203,6 |  | to | 99,6 |  | 164,7 |  | to | 99,6 |  |
| *Potamotrygon* | **11,6** | to | **0,0** | 136,4 |  | to | 0,0 |  | 93,5 |  | to | 0,0 |  |
| *Praeproscyllium* | **167,7** | to | **164,7** | 167,7 |  | to | 164,7 |  | 167,7 |  | to | 164,7 |  |
| *PremontreiaO* | **65,5** | to | **33,9** | 65,5 |  | to | 33,9 |  | 164,7 |  | to | 33,9 |  |
| *PremontreiaP* | **55,8** | to | **33,9** | 167,7 | 55,8 | to | 33,9 |  | 164,7 | 55,8 | to | 33,9 |  |
| *Priohybodus* | **155,7** | to | **99,6** | 203,6 |  | to | 99,6 |  | 164,7 |  | to | 99,6 |  |
| *Prionace* | **5,3** | to | **0,0** | 55,8 | 48,6 | to | 0,0 |  | 5,3 |  | to | 0,0 |  |
| *Priscurus* | **0,0** | to | **0,0** | 140,2 |  | to | 0,0 |  | 125,0 |  | to | 0,0 |  |
| *Pristiophorus* | **112,0** | to | **0,0** | 161,2 |  | to | 0,0 |  | 150,8 |  | to | 0,0 |  |
| *Pristis* | **55,8** | to | **0,0** | 183,0 | 55,8 | to | 0,0 |  | 183,0 | 99,6 | to | 0,0 |  |
| *Proetmopterus* | **83,5** | to | **65,5** | 83,5 |  | to | 65,5 |  | 83,5 |  | to | 65,5 |  |
| *Prohaploblepharus* | **89,3** | to | **70,6** | 89,3 |  | to | 70,6 |  | 164,7 |  | to | 70,6 |  |
| *Proheterodontus* | **167,7** | to | **150,8** | 183,0 |  | to | 150,8 |  | 167,7 |  | to | 150,8 |  |
| *Propristis* | **55,8** | to | **33,9** | 183,0 | 55,8 | to | 33,9 |  | 183,0 | 55,8 | to | 33,9 |  |
| *Proscyllium* | **0,0** | to | **0,0** | 167,7 |  | to | 0,0 |  | 164,7 |  | to | 0,0 |  |
| *Proscymnodon* | **0,0** | to | **0,0** | 93,5 | 85,8 | to | 0,0 |  | 70,6 |  | to | 0,0 |  |
| *Protocentrophorus* | **99,6** | to | **70,6** | 130,0 | 99,6 | to | 70,6 |  | 99,6 |  | to | 70,6 |  |
| *Protoginglymostoma* | **55,8** | to | **40,4** | 130,0 |  | to | 40,4 |  | 70,6 | 55,8 | to | 40,4 |  |
| *Protolamna* | **140,2** | to | **65,5** | 140,2 |  | to | 65,5 |  | 183,0 |  | to | 65,5 |  |
| *Protoplatyrhina* | **83,5** | to | **65,5** | 183,0 |  | to | 65,5 |  | 183,0 | 175,6 | to | 65,5 |  |
| *Protoscyliorhinus* | **130,0** | to | **83,5** | 167,7 |  | to | 83,5 |  | 164,7 |  | to | 83,5 |  |
| *Protospinax* | **183,0** | to | **145,5** | 196,5 | 183,0 | to | 145,5 |  | 183,0 |  | to | 145,5 |  |
| *Protosqualus* | **130,0** | to | **93,5** | 130,0 |  | to | 93,5 |  | 150,8 | 145,5 | to | 93,5 |  |
| *Protoxynotus* | **93,5** | to | **70,6** | 93,5 |  | to | 70,6 |  | 93,5 |  | to | 70,6 |  |
| *Psammobatis* | **0,0** | to | **0,0** | 99,6 |  | to | 0,0 |  | 99,6 |  | to | 0,0 |  |
| *Pseudaetobatus* | **55,8** | to | **40,4** | 83,5 |  | to | 40,4 |  | 70,6 |  | to | 40,4 |  |
| *Pseudocarcharias* | **20,4** | to | **0,0** | 136,4 | 99,6 | to | 0,0 |  | 65,5 |  | to | 0,0 |  |
| *Pseudocetorhinus* | **203,6** | to | **199,6** | 270,0 |  | to | 199,6 |  | 245,0 |  | to | 199,6 |  |
| *Pseudocorax* | **93,5** | to | **65,5** | 93,5 |  | to | 65,5 |  | 93,5 |  | to | 65,5 |  |
| *Pseudodalatias* | **203,6** | to | **199,6** | 251,0 |  | to | 199,6 |  | 245,0 |  | to | 199,6 |  |
| *Pseudoechinorhinus* | **65,5** | to | **61,7** | 112,0 |  | to | 61,7 |  | 65,5 |  | to | 61,7 |  |
| *Pseudoginglymostoma* | **70,6** | to | **0,0** | 130,0 |  | to | 0,0 |  | 70,6 |  | to | 0,0 |  |
| *Pseudohypolophus* | **125,0** | to | **93,5** | 183,0 |  | to | 93,5 |  | 175,6 |  | to | 93,5 |  |
| *Pseudonotidanus* | **183,0** | to | **155,7** | 196,5 |  | to | 155,7 |  | 183,0 |  | to | 155,7 |  |
| *Pseudoraja* | **0,0** | to | **0,0** | 0,0 |  | to | 0,0 |  | 0,0 |  | to | 0,0 |  |
| *Pseudorhina* | **150,8** | to | **93,5** | 161,2 |  | to | 93,5 |  | 150,8 |  | to | 93,5 |  |
| *Pseudoscapanorhynchus* | **112,0** | to | **89,3** | 140,2 |  | to | 89,3 |  | 125,0 |  | to | 89,3 |  |
| *Pseudoscyliorhinus* | **99,6** | to | **70,6** | 167,7 |  | to | 70,6 |  | 164,7 |  | to | 70,6 |  |
| *Pseudospinax* | **0,0** | to | **0,0** | 196,5 |  | to | 0,0 |  | 183,0 |  | to | 0,0 |  |
| *Pseudotriakis* | **0,0** | to | **0,0** | 167,7 | 0,0 | to | 0,0 |  | 164,0 | 0,0 | to | 0,0 |  |
| *Pteromylaeus* | **16,0** | to | **0,0** | 65,5 |  | to | 0,0 |  | 16,0 |  | to | 0,0 |  |
| *Pteroplatea* | **0,0** | to | **0,0** | 270,0 | 99,6 | to | 0,0 |  | 40,4 |  | to | 0,0 |  |
| *Pteroplatytrygon* | **0,0** | to | **0,0** | 136,4 |  | to | 0,0 |  | 93,5 | 61,7 | to | 0,0 |  |
| *Pteroscyllium* | **125,0** | to | **65,5** | 167,7 |  | to | 65,5 |  | 164,7 |  | to | 65,5 |  |
| *Ptychocorax* | **85,8** | to | **70,6** | 112,0 |  | to | 70,6 |  | 85,8 |  | to | 70,6 |  |
| *Ptychodus* | **112,0** | to | **70,6** | 130,0 |  | to | 70,6 |  | 112,0 |  | to | 70,6 |  |
| *Ptychotrygon* | **112,0** | to | **65,5** | 183,0 |  | to | 65,5 |  | 183,0 |  | to | 65,5 |  |
| *Ptychotrygonoides* | **99,6** | to | **89,3** | 183,0 |  | to | 89,3 |  | 183,0 |  | to | 89,3 |  |
| *Pucabatis* | **70,6** | to | **65,5** | 83,5 |  | to | 65,5 |  | 70,6 |  | to | 65,5 |  |
| *Pucapristis* | **70,6** | to | **65,5** | 93,5 |  | to | 65,5 |  | 70,6 |  | to | 65,5 |  |
| *Pueblocarcharias* | **85,8** | to | **83,5** | 136,4 |  | to | 83,5 |  | 125,0 |  | to | 83,5 |  |
| *Raineria* | **203,6** | to | **199,6** | 251,0 |  | to | 199,6 |  | 251,0 |  | to | 199,6 |  |
| *Raja* | **70,6** | to | **0,0** | 99,6 |  | to | 0,0 |  | 93,5 |  | to | 0,0 |  |
| *Rajella* | **0,0** | to | **0,0** | 99,6 |  | to | 0,0 |  | 93,5 |  | to | 0,0 |  |
| *Rajorhina* | **99,6** | to | **93,5** | 99,6 |  | to | 93,5 |  | 99,6 |  | to | 93,5 |  |
| *Reifia* | **216,5** | to | **203,6** | 270,0 |  | to | 203,6 |  | 245,0 |  | to | 203,6 |  |
| *Renpetia* | **99,6** | to | **93,5** | 130,0 |  | to | 93,5 |  | 125,0 |  | to | 93,5 |  |
| *Reticulodus* | **216,5** | to | **203,6** | 251,0 |  | to | 203,6 |  | 216,0 |  | to | 203,6 |  |
| *Rhina* | **23,0** | to | **0,0** | 183,0 |  | to | 0,0 |  | 175,6 | 23,0 | to | 0,0 |  |
| *Rhincodon* | **16,0** | to | **0,0** | 58,7 |  | to | 0,0 |  | 40,4 |  | to | 0,0 |  |
| *Rhinobatos* | **130,0** | to | **0,0** | 183,0 |  | to | 0,0 |  | 183,0 | 175,6 | to | 0,0 |  |
| *Rhinoptera* | **58,7** | to | **0,0** | 58,7 |  | to | 0,0 |  | 58,7 |  | to | 0,0 |  |
| *Rhinoraja* | **0,0** | to | **0,0** | 0,0 |  | to | 0,0 |  | 0,0 |  | to | 0,0 |  |
| *Rhizoprionodon* | **55,8** | to | **0,0** | 65,5 | 55,8 | to | 0,0 |  | 61,7 | 55,8 | to | 0,0 |  |
| *Rhombodus* | **83,5** | to | **65,5** | 83,5 |  | to | 65,5 |  | 99,6 | 83,5 | to | 65,5 |  |
| *Rhombopterygia* | **99,6** | to | **93,5** | 183,0 |  | to | 93,5 |  | 175,6 |  | to | 93,5 |  |
| *Rhomphaiodon* | **216,5** | to | **199,6** | 245,0 |  | to | 199,6 |  | 237,0 |  | to | 199,6 |  |
| *Rhynchobatus* | **55,8** | to | **0,0** | 183,0 | 55,8 | to | 0,0 |  | 183,0 | 175,6 | to | 0,0 |  |
| *Rioraja* | **0,0** | to | **0,0** | 0,0 |  | to | 0,0 |  | 0,0 |  | to | 0,0 |  |
| *Rostroraja* | **0,0** | to | **0,0** | 99,6 |  | to | 0,0 |  | 93,5 |  | to | 0,0 |  |
| *Roullletia* | **0,0** | to | **0,0** | 136,4 | 125,0 | to | 0,0 |  | 125,0 | 93,5 | to | 0,0 |  |
| *Safagaia* | **70,6** | to | **65,5** | 183,0 |  | to | 65,5 |  | 183,0 |  | to | 65,5 |  |
| *Scapanorhynchus* | **112,0** | to | **65,5** | 130,0 | 125,0 | to | 65,5 |  | 112,0 |  | to | 65,5 |  |
| *Schizorhiza* | **85,8** | to | **65,5** | 93,5 |  | to | 65,5 |  | 85,8 |  | to | 65,5 |  |
| *Schroederichthys* | **0,0** | to | **0,0** | 167,7 |  | to | 0,0 |  | 164,7 | 55,8 | to | 0,0 |  |
| *Scindocorax* | **85,8** | to | **83,5** | 112,0 |  | to | 83,5 |  | 85,8 |  | to | 83,5 |  |
| *Sclerorhynchus* | **93,5** | to | **65,5** | 130,0 |  | to | 65,5 |  | 93,5 |  | to | 65,5 |  |
| *Scoliodon* | **23,0** | to | **0,0** | 55,8 |  | to | 0,0 |  | 48,6 | 23,0 | to | 0,0 |  |
| *Scyliorhinus* | **140,2** | to | **0,0** | 140,2 |  | to | 0,0 |  | 164,7 |  | to | 0,0 |  |
| *Scylliogaleus* | **0,0** | to | **0,0** | 99,6 | 93,5 | to | 0,0 |  | 70,6 |  | to | 0,0 |  |
| *Scymnodalatias* | **48,6** | to | **0,0** | 85,8 |  | to | 0,0 |  | 70,6 |  | to | 0,0 |  |
| *Scymnodon* | **48,6** | to | **0,0** | 85,8 |  | to | 0,0 |  | 70,6 |  | to | 0,0 |  |
| *Secarodus* | **167,7** | to | **164,7** | 203,6 |  | to | 164,7 |  | 167,7 |  | to | 164,7 |  |
| *Serratolamna* | **83,5** | to | **65,5** | 136,4 | 125,0 | to | 65,5 |  | 125,0 | 83,5 | to | 65,5 |  |
| *Sinobatis* | **0,0** | to | **0,0** | 0,0 |  | to | 0,0 |  | 0,0 |  | to | 0,0 |  |
| *Smithraja* | **58,7** | to | **40,4** | 99,6 |  | to | 40,4 |  | 93,5 |  | to | 40,4 |  |
| *SomniosusR* | **5,3** | to | **0,0** | 48,6 |  | to | 0,0 |  | 5,3 |  | to | 0,0 |  |
| *SomniosusS* | **48,6** | to | **0,0** | 85,8 |  | to | 0,0 |  | 83,5 |  | to | 0,0 |  |
| *Spathobatis* | **175,6** | to | **125,0** | 183,0 |  | to | 125,0 |  | 183,0 | 175,6 | to | 125,0 |  |
| *Sphenodus* | **171,6** | to | **61,7** | 196,5 |  | to | 61,7 |  | 183,0 |  | to | 61,7 |  |
| *Sphyrna* | **20,4** | to | **0,0** | 65,5 | 20,4 | to | 0,0 |  | 65,5 |  | to | 0,0 |  |
| *Squalicorax* | **112,0** | to | **65,5** | 112,0 |  | to | 65,5 |  | 125,0 | 112,0 | to | 65,5 |  |
| *Squaliodalatias* | **48,6** | to | **37,2** | 83,5 |  | to | 37,2 |  | 70,6 |  | to | 37,2 |  |
| *Squaliolus* | **48,6** | to | **0,0** | 48,6 |  | to | 0,0 |  | 48,6 |  | to | 0,0 |  |
| *Squalus* | **99,6** | to | **0,0** | 130,0 |  | to | 0,0 |  | 99,6 |  | to | 0,0 |  |
| *Squatigaleus* | **89,3** | to | **65,5** | 99,6 | 93,5 | to | 65,5 |  | 89,3 |  | to | 65,5 |  |
| *Squatina* | **161,2** | to | **0,0** | 183,0 | 161,2 | to | 0,0 |  | 183,0 | 161,2 | to | 0,0 |  |
| *Squatirhina* | **112,0** | to | **65,5** | 183,0 |  | to | 65,5 |  | 175,6 |  | to | 65,5 |  |
| *Squatiscyllium* | **58,7** | to | **40,4** | 58,7 |  | to | 40,4 |  | 58,7 |  | to | 40,4 |  |
| *Stegostoma* | **55,8** | to | **0,0** | 130,0 |  | to | 0,0 |  | 70,6 |  | to | 0,0 |  |
| *Steinbachodus* | **228,0** | to | **216,5** | 251,0 |  | to | 216,5 |  | 251,0 |  | to | 216,5 |  |
| *Stenoscyllium* | **55,8** | to | **48,6** | 167,7 |  | to | 48,6 |  | 164,7 |  | to | 48,6 |  |
| *Striatolamia* | **65,5** | to | **33,9** | 130,0 | 125,0 | to | 33,9 |  | 99,6 | 65,5 | to | 33,9 |  |
| *Sutorectus* | **0,0** | to | **0,0** | 112,0 |  | to | 0,0 |  | 70,6 |  | to | 0,0 |  |
| *Sylvestrialmia* | **0,0** | to | **0,0** | 136,4 | 125,0 | to | 0,0 |  | 125,0 | 93,5 | to | 0,0 |  |
| *Sympterygia* | **0,0** | to | **0,0** | 0,0 |  | to | 0,0 |  | 0,0 |  | to | 0,0 |  |
| *Synechodus* | **270,0** | to | **48,6** | 270,0 |  | to | 48,6 |  | 270,0 |  | to | 48,6 |  |
| *Taeniura* | **0,0** | to | **0,0** | 136,4 |  | to | 0,0 |  | 93,5 | 61,7 | to | 0,0 |  |
| *Taeniurops* | **0,0** | to | **0,0** | 136,4 |  | to | 0,0 |  | 93,5 | 61,7 | to | 0,0 |  |
| *Tanoutia* | **70,6** | to | **65,5** | 183,0 |  | to | 65,5 |  | 183,0 |  | to | 65,5 |  |
| *Telodontaspis* | **99,6** | to | **93,5** | 125,0 |  | to | 93,5 |  | 99,6 |  | to | 93,5 |  |
| *Temera* | **0,0** | to | **0,0** | 0,0 |  | to | 0,0 |  | 0,0 |  | to | 0,0 |  |
| *Tethybatis* | **83,5** | to | **65,5** | 83,5 |  | to | 65,5 |  | 183,0 | 175,6 | to | 65,5 |  |
| *Texabatis* | **70,6** | to | **65,5** | 136,4 |  | to | 65,5 |  | 93,5 |  | to | 65,5 |  |
| *Texatrygon* | **99,6** | to | **65,5** | 183,0 |  | to | 65,5 |  | 183,0 |  | to | 65,5 |  |
| *Thaiodus* | **125,0** | to | **99,6** | 203,6 |  | to | 99,6 |  | 164,7 |  | to | 99,6 |  |
| *Tingaleus* | **58,7** | to | **55,8** | 65,5 |  | to | 55,8 |  | 58,7 |  | to | 55,8 |  |
| *Titanonarke* | **55,8** | to | **48,6** | 65,5 | 58,7 | to | 48,6 |  | 55,8 |  | to | 48,6 |  |
| *Toarcibatis* | **183,0** | to | **175,6** | 183,0 |  | to | 175,6 |  | 183,0 |  | to | 175,6 |  |
| *Torpedo* | **58,7** | to | **0,0** | 183,0 | 65,0 | to | 0,0 |  | 58,7 |  | to | 0,0 |  |
| *Triaenodon* | **0,0** | to | **0,0** | 55,8 |  | to | 0,0 |  | 48,6 | 0,0 | to | 0,0 |  |
| *Triakis* | **65,5** | to | **0,0** | 99,6 |  | to | 0,0 |  | 70,6 |  | to | 0,0 |  |
| *Tribodus* | **125,0** | to | **93,5** | 125,0 |  | to | 93,5 |  | 203,6 |  | to | 93,5 |  |
| *Trigonognathus* | **48,6** | to | **0,0** | 83,5 |  | to | 0,0 |  | 83,5 |  | to | 0,0 |  |
| *Trigonotodus* | **55,8** | to | **37,2** | 125,0 |  | to | 37,2 |  | 99,6 |  | to | 37,2 |  |
| *Trygonoptera* | **0,0** | to | **0,0** | 58,7 |  | to | 0,0 |  | 0,0 |  | to | 0,0 |  |
| *Trygonorrhina* | **0,0** | to | **0,0** | 183,0 |  | to | 0,0 |  | 175,6 | 0,0 | to | 0,0 |  |
| *Turania* | **40,4** | to | **37,2** | 136,4 | 125,0 | to | 37,2 |  | 125,0 | 93,5 | to | 37,2 |  |
| *Turoniabatis* | **112,0** | to | **93,5** | 183,0 |  | to | 93,5 |  | 183,0 |  | to | 93,5 |  |
| *Typhlonarke* | **0,0** | to | **0,0** | 0,0 |  | to | 0,0 |  | 0,0 |  | to | 0,0 |  |
| *Urobatis* | **48,6** | to | **0,0** | 48,6 |  | to | 0,0 |  | 93,5 |  | to | 0,0 |  |
| *Urogymnus* | **0,0** | to | **0,0** | 136,4 |  | to | 0,0 |  | 93,5 | 61,7 | to | 0,0 |  |
| *Urolophus* | **55,8** | to | **0,0** | 136,4 | 55,8 | to | 0,0 |  | 58,7 | 55,8 | to | 0,0 |  |
| *Urotrygon* | **0,0** | to | **0,0** | 136,4 |  | to | 0,0 |  | 0,0 |  | to | 0,0 |  |
| *Usakias* | **40,4** | to | **37,2** | 99,6 |  | to | 37,2 |  | 65,5 |  | to | 37,2 |  |
| *Vallisia* | **203,6** | to | **199,6** | 270,0 |  | to | 199,6 |  | 251,0 |  | to | 199,6 |  |
| *Vascobatis* | **70,6** | to | **65,5** | 183,0 |  | to | 65,5 |  | 183,0 | 175,6 | to | 65,5 |  |
| *Vectiselachos* | **164,7** | to | **125,0** | 251,0 |  | to | 125,0 |  | 216,5 |  | to | 125,0 |  |
| *Viperecucullus* | **65,5** | to | **55,8** | 136,4 |  | to | 55,8 |  | 93,5 |  | to | 55,8 |  |
| *Walteraja* | **70,6** | to | **65,5** | 99,6 |  | to | 65,5 |  | 93,5 |  | to | 65,5 |  |
| *Washakiebatis* | **55,8** | to | **48,6** | 83,5 |  | to | 48,6 |  | 65,5 |  | to | 48,6 |  |
| *Welcommia* | **183,0** | to | **136,4** | 183,0 |  | to | 136,4 |  | 183,0 |  | to | 136,4 |  |
| *Weltonia* | **58,7** | to | **48,6** | 145,5 |  | to | 48,6 |  | 58,7 |  | to | 48,6 |  |
| *Woellsteinia* | **48,6** | to | **28,5** | 125,0 |  | to | 28,5 |  | 65,5 |  | to | 28,5 |  |
| *Xiphodolamia* | **55,8** | to | **33,9** | 65,5 |  | to | 33,9 |  | 55,8 |  | to | 33,9 |  |
| *Xystrogaleus* | **48,6** | to | **40,4** | 99,6 | 93,5 | to | 40,4 |  | 70,6 |  | to | 40,4 |  |
| *Youssoubatis* | **83,5** | to | **65,5** | 83,5 |  | to | 65,5 |  | 83,5 |  | to | 65,5 |  |
| *Zameus* | **5,3** | to | **0,0** | 93,5 | 48,6 | to | 0,0 |  | 5,3 |  | to | 0,0 |  |
| *Zanobatus* | **0,0** | to | **0,0** | 183,0 | 136,4 | to | 0,0 |  | 183,0 | 99,6 | to | 0,0 |  |
| *Zapteryx* | **0,0** | to | **0,0** | 183,0 | 99,6 | to | 0,0 |  | 175,6 | 0,0 | to | 0,0 |  |
| *Zearaja* | **0,0** | to | **0,0** | 99,6 |  | to | 0,0 |  | 93,5 |  | to | 0,0 |  |
|  |  |  |  |  |  |  |  |  |  |  |  |  |  |
| **FAMILIES** |  |  |  |  |  |  |  |  |  |  |  |  |  |
|  |  | **Observed** |  |  |  | **CBM** |  |  |  |  | **DDBM** |  |  |
|  | **FAD** |  | **LAD** | **FAD** | |  | **LAD** | | **FAD** | |  | **LAD** | |
| Acrodontidae | **228,0** | to | **83,5** | 228,0 |  | to | 83,5 |  | 228,0 |  | to | 83,5 |  |
| Agaleidae | **196,5** | to | **183,0** | 196,5 |  | to | 183,0 |  | 196,5 |  | to | 183,0 |  |
| Alopiidae | **55,8** | to | **0,0** | 140,2 | 136,4 | to | 0,0 |  | 93,5 |  | to | 0,0 |  |
| Anacanthobatidae | **0,0** | to | **0,0** | 183,0 | 99,6 | to | 0,0 |  | 0,0 |  | to | 0,0 |  |
| Anacoracidae | **112,0** | to | **65,5** | 136,4 |  | to | 65,5 |  | 112,0 |  | to | 65,5 |  |
| Archaeobatidae | **183,0** | to | **175,6** | 183,0 |  | to | 175,6 |  | 196,5 | 183,0 | to | 175,6 |  |
| Archaeolamnidae | **112,0** | to | **65,5** | 140,2 |  | to | 65,5 |  | 112,0 |  | to | 65,5 |  |
| Arhynchobatidae | **0,0** | to | **0,0** | 99,6 |  | to | 0,0 |  | 0,0 |  | to | 0,0 |  |
| Brachaeluridae | **175,6** | to | **0,0** | 175,6 |  | to | 0,0 |  | 183,0 |  | to | 0,0 |  |
| Carcharhinidae | **65,5** | to | **0,0** | 65,5 |  | to | 0,0 |  | 65,5 |  | to | 0,0 |  |
| Cardabiodontidae | **112,0** | to | **89,3** | 136,4 |  | to | 89,3 |  | 112,0 |  | to | 89,3 |  |
| Centrophoridae | **99,6** | to | **0,0** | 270,0 | 130,0 | to | 0,0 |  | 99,6 |  | to | 0,0 |  |
| Cetorhinidae | **48,6** | to | **0,0** | 136,4 | 65,5 | to | 0,0 |  | 93,5 | 48,6 | to | 0,0 |  |
| Chlamydoselachidae | **85,8** | to | **0,0** | 270,0 | 196,5 | to | 0,0 |  | 136,4 |  | to | 0,0 |  |
| Cretoxyrhinidae | **112,0** | to | **33,9** | 140,2 |  | to | 33,9 |  | 112,0 |  | to | 33,9 |  |
| Cyclobatidae | **99,6** | to | **93,5** | 99,6 |  | to | 93,5 |  | 99,6 |  | to | 93,5 |  |
| Dalatiidae | **83,5** | to | **0,0** | 130,0 | 99,6 | to | 0,0 |  | 83,5 |  | to | 0,0 |  |
| Dasyatidae | **99,6** | to | **0,0** | 99,6 |  | to | 0,0 |  | 175,6 | 99,6 | to | 0,0 |  |
| Distobatidae | **125,0** | to | **93,5** | 228,0 |  | to | 93,5 |  | 245,0 |  | to | 93,5 |  |
| Echinorhinidae | **136,4** | to | **0,0** | 270,0 | 161,2 | to | 0,0 |  | 196,5 | 136,4 | to | 0,0 |  |
| Eoptolamnidae | **130,0** | to | **99,6** | 270,0 | 196,5 | to | 99,6 |  | 130,0 |  | to | 99,6 |  |
| Etmopteridae | **83,5** | to | **0,0** | 130,0 | 99,6 | to | 0,0 |  | 83,5 |  | to | 0,0 |  |
| Ginglymostomatidae | **130,0** | to | **0,0** | 130,0 |  | to | 0,0 |  | 150,8 |  | to | 0,0 |  |
| Gymnuridae | **58,7** | to | **0,0** | 83,5 | 58,7 | to | 0,0 |  | 83,5 | 58,7 | to | 0,0 |  |
| Hemigaleidae | **40,4** | to | **0,0** | 136,4 | 65,5 | to | 0,0 |  | 40,4 |  | to | 0,0 |  |
| Hemiscylliidae | **125,0** | to | **0,0** | 175,6 |  | to | 0,0 |  | 125,0 |  | to | 0,0 |  |
| Heptranchidae | **83,5** | to | **0,0** | 196,5 |  | to | 0,0 |  | 83,5 |  | to | 0,0 |  |
| Heterodontidae | **183,0** | to | **0,0** | 270,0 | 183,0 | to | 0,0 |  | 196,5 | 183,0 | to | 0,0 |  |
| Hexanchidae | **196,5** | to | **0,0** | 196,5 |  | to | 0,0 |  | 196,5 |  | to | 0,0 |  |
| Hexatrygonidae | **48,6** | to | **0,0** | 99,6 | 58,7 | to | 0,0 |  | 83,5 | 48,6 | to | 0,0 |  |
| Hybodontidae | **216,5** | to | **65,5** | 251,0 |  | to | 65,5 |  | 228,0 |  | to | 65,5 |  |
| Hypnidae | **0,0** | to | **0,0** | 65,5 |  | to | 0,0 |  | 0,0 |  | to | 0,0 |  |
| Hypsobatidae | **83,5** | to | **65,5** | 183,0 |  | to | 65,5 |  | 83,5 |  | to | 65,5 |  |
| Lamnidae | **65,5** | to | **0,0** | 136,4 |  | to | 0,0 |  | 93,5 |  | to | 0,0 |  |
| Leptochariidae | **0,0** | to | **0,0** | 167,7 | 136,4 | to | 0,0 |  | 65,5 | 40,4 | to | 0,0 |  |
| Lonchidiidae | **251,0** | to | **65,5** | 270,0 |  | to | 65,5 |  | 251,0 |  | to | 65,5 |  |
| Megachasmidae | **28,4** | to | **0,0** | 136,4 | 55,8 | to | 0,0 |  | 93,5 | 28,4 | to | 0,0 |  |
| Mitsukurinidae | **125,0** | to | **0,0** | 140,2 |  | to | 0,0 |  | 125,0 |  | to | 0,0 |  |
| Mobulidae | **58,7** | to | **0,0** | 58,7 |  | to | 0,0 |  | 58,7 |  | to | 0,0 |  |
| Myliobatidae | **83,5** | to | **0,0** | 83,5 |  | to | 0,0 |  | 83,5 |  | to | 0,0 |  |
| Narcinidae | **58,7** | to | **0,0** | 65,5 | 58,7 | to | 0,0 |  | 58,7 |  | to | 0,0 |  |
| Narkidae | **0,0** | to | **0,0** | 183,0 |  | to | 0,0 |  | 0,0 |  | to | 0,0 |  |
| Odontaspididae | **136,4** | to | **0,0** | 140,2 | 136,4 | to | 0,0 |  | 136,4 |  | to | 0,0 |  |
| Orectolobidae | **150,8** | to | **0,0** | 175,6 |  | to | 0,0 |  | 150,8 |  | to | 0,0 |  |
| Orthacodontidae | **171,6** | to | **61,7** | 196,5 |  | to | 61,7 |  | 171,6 |  | to | 61,7 |  |
| Ostenoselachidae | **196,5** | to | **189,6** | 251,0 |  | to | 189,6 |  | 245,0 |  | to | 189,6 |  |
| Otodontidae | **65,5** | to | **2,6** | 136,4 |  | to | 2,6 |  | 93,5 |  | to | 2,6 |  |
| Oxynotidae | **93,5** | to | **0,0** | 130,0 | 99,6 | to | 0,0 |  | 93,5 |  | to | 0,0 |  |
| Palaeospinacidae | **270,0** | to | **61,7** | 270,0 |  | to | 61,7 |  | 270,0 |  | to | 61,7 |  |
| Paraisuridae | **125,0** | to | **99,6** | 140,2 |  | to | 99,6 |  | 125,0 |  | to | 99,6 |  |
| Paraorthacodontidae | **196,5** | to | **55,8** | 196,5 |  | to | 55,8 |  | 196,5 |  | to | 55,8 |  |
| Parapalaeobatidae | **89,3** | to | **65,5** | 183,0 |  | to | 65,5 |  | 89,3 |  | to | 65,5 |  |
| Parascylliidae | **112,0** | to | **0,0** | 270,0 | 196,5 | to | 0,0 |  | 150,8 |  | to | 0,0 |  |
| Platyrhinidae | **83,5** | to | **0,0** | 183,0 |  | to | 0,0 |  | 175,6 | 83,5 | to | 0,0 |  |
| Plesiobatidae | **58,7** | to | **0,0** | 58,7 |  | to | 0,0 |  | 83,5 |  | to | 0,0 |  |
| Polyacrodontidae | **245,0** | to | **70,6** | 251,0 |  | to | 70,6 |  | 245,0 |  | to | 70,6 |  |
| Potamotrygonidae | **11,6** | to | **0,0** | 99,6 |  | to | 0,0 |  | 83,5 | 11,6 | to | 0,0 |  |
| Pristidae | **55,8** | to | **0,0** | 270,0 | 183,0 | to | 0,0 |  | 175,6 | 83,5 | to | 0,0 |  |
| Pristiophoridae | **112,0** | to | **0,0** | 196,5 | 161,2 | to | 0,0 |  | 136,4 | 112,0 | to | 0,0 |  |
| Proscylliidae | **167,7** | to | **0,0** | 167,7 | 167,7 | to | 0,0 |  | 167,7 |  | to | 0,0 |  |
| Protospinacidae | **183,0** | to | **93,5** | 270,0 | 196,5 | to | 93,5 |  | 196,5 |  | to | 93,5 |  |
| Pseudocarchariidae | **20,4** | to | **0,0** | 136,4 | 55,8 | to | 0,0 |  | 93,5 | 20,4 | to | 0,0 |  |
| Pseudocoracidae | **93,5** | to | **65,5** | 112,0 |  | to | 65,5 |  | 93,5 |  | to | 65,5 |  |
| Pseudodalatiidae | **203,6** | to | **199,6** | 251,0 |  | to | 199,6 |  | 245,0 |  | to | 199,6 |  |
| Pseudonotidanidae | **183,0** | to | **136,4** | 196,5 |  | to | 136,4 |  | 183,0 |  | to | 136,4 |  |
| Pseudoscapanorhynchidae | **140,2** | to | **65,5** | 140,2 |  | to | 65,5 |  | 196,5 | 183,0 | to | 65,5 |  |
| Pseudotriakidae | **0,0** | to | **0,0** | 167,7 |  | to | 0,0 |  | 65,5 | 0,0 | to | 0,0 |  |
| Ptychodontidae | **125,0** | to | **70,6** | 251,0 |  | to | 70,6 |  | 245,0 |  | to | 70,6 |  |
| Rajidae | **99,6** | to | **0,0** | 99,6 |  | to | 0,0 |  | 175,6 | 99,6 | to | 0,0 |  |
| Rhincodontidae | **58,7** | to | **0,0** | 130,0 |  | to | 0,0 |  | 58,7 |  | to | 0,0 |  |
| Rhinidae | **23,0** | to | **0,0** | 183,0 |  | to | 0,0 |  | 65,5 |  | to | 0,0 |  |
| Rhinobatidae | **183,0** | to | **0,0** | 183,0 | 99,6 | to | 0,0 |  | 196,5 | 183,0 | to | 0,0 |  |
| Rhinopteridae | **58,7** | to | **0,0** | 270,0 | 99,6 | to | 0,0 |  | 65,5 |  | to | 0,0 |  |
| Rhombodontidae | **83,5** | to | **65,5** | 83,5 |  | to | 65,5 |  | 83,5 |  | to | 65,5 |  |
| Rhynchobatidae | **55,8** | to | **0,0** | 270,0 | 183,0 | to | 0,0 |  | 65,5 |  | to | 0,0 |  |
| Sclerorhynchidae | **130,0** | to | **65,5** | 183,0 |  | to | 65,5 |  | 175,6 |  | to | 65,5 |  |
| Scyliorhinidae | **167,7** | to | **0,0** | 196,5 | 167,7 | to | 0,0 |  | 183,0 | 167,7 | to | 0,0 |  |
| Serratolamnidae | **83,5** | to | **65,5** | 136,4 |  | to | 65,5 |  | 93,5 |  | to | 65,5 |  |
| Somniosidae | **85,8** | to | **0,0** | 99,6 | 93,5 | to | 0,0 |  | 85,8 |  | to | 0,0 |  |
| Sphyrnidae | **33,9** | to | **0,0** | 65,5 |  | to | 0,0 |  | 33,9 |  | to | 0,0 |  |
| Squalidae | **130,0** | to | **0,0** | 270,0 | 130,0 | to | 0,0 |  | 136,4 | 130,0 | to | 0,0 |  |
| Squatinidae | **161,2** | to | **0,0** | 161,2 |  | to | 0,0 |  | 196,5 | 161,2 | to | 0,0 |  |
| Stegostomatidae | **55,8** | to | **0,0** | 130,0 |  | to | 0,0 |  | 55,8 |  | to | 0,0 |  |
| Steinbachodontidae | **228,0** | to | **216,5** | 251,0 |  | to | 216,5 |  | 245,0 |  | to | 216,5 |  |
| Torpedinidae | **65,5** | to | **0,0** | 65,5 |  | to | 0,0 |  | 175,6 | 99,6 | to | 0,0 |  |
| Triakidae | **136,4** | to | **0,0** | 136,4 |  | to | 0,0 |  | 136,4 |  | to | 0,0 |  |
| Urolophidae | **55,8** | to | **0,0** | 183,0 | 99,6 | to | 0,0 |  | 55,8 |  | to | 0,0 |  |
| Urotrygonidae | **55,8** | to | **0,0** | 99,6 |  | to | 0,0 |  | 83,5 |  | to | 0,0 |  |
| Waipitiodidae | **203,6** | to | **199,6** | 251,0 |  | to | 199,6 |  | 245,0 |  | to | 199,6 |  |
|  |  |  |  |  |  |  |  |  |  |  |  |  |  |
| **ORDERS** |  |  |  |  |  |  |  |  |  |  |  |  |  |
|  |  | **Observed** |  |  |  | **CBM** |  |  |  |  | **DDBM** |  |  |
|  | **FAD** |  | **LAD** | **FAD** | |  | **LAD** | | **FAD** | |  | **LAD** | |
| Archaeobatid | **183,0** | to | **175,6** | 270,0 | 183,0 | to | 175,6 |  | 196,5 | 183,0 | to | 175,6 |  |
| Carcharhiniformes | **167,7** | to | **0,0** | 175,6 | 167,7 | to | 0,0 |  | 183,0 | 167,7 | to | 0,0 |  |
| Echinorhinid | **136,4** | to | **0,0** | 196,5 | 161,2 | to | 0,0 |  | 183,0 | 136,4 | to | 0,0 |  |
| Heterodontiformes | **183,0** | to | **0,0** | 196,5 | 183,0 | to | 0,0 |  | 183,0 |  | to | 0,0 |  |
| Hexanchiformes | **196,5** | to | **0,0** | 196,5 |  | to | 0,0 |  | 196,5 |  | to | 0,0 |  |
| Hybodontiformes | **251,0** | to | **65,5** | 270,0 |  | to | 65,5 |  | 251,0 |  | to | 65,5 |  |
| Lamniformes | **140,2** | to | **0,0** | 270,0 | 183,0 | to | 0,0 |  | 167,7 | 140,2 | to | 0,0 |  |
| Myliobatiformes | **136,4** | to | **0,0** | 183,0 | 136,4 | to | 0,0 |  | 175,6 | 136,4 | to | 0,0 |  |
| Orectolobiformes | **175,6** | to | **0,0** | 196,5 | 175,6 | to | 0,0 |  | 183,0 | 175,6 | to | 0,0 |  |
| Pristid | **55,8** | to | **0,0** | 183,0 | 136,4 | to | 0,0 |  | 175,6 | 55,8 | to | 0,0 |  |
| Pristiophoriformes | **112,0** | to | **0,0** | 270,0 | 161,2 | to | 0,0 |  | 161,2 | 112,0 | to | 0,0 |  |
| Protospinacid | **183,0** | to | **93,5** | 196,5 |  | to | 93,5 |  | 183,0 |  | to | 93,5 |  |
| Rajid | **99,6** | to | **0,0** | 270,0 | 183,0 | to | 0,0 |  | 175,6 | 99,6 | to | 0,0 |  |
| Rhinobatid | **183,0** | to | **0,0** | 183,0 |  | to | 0,0 |  | 196,5 | 183,0 | to | 0,0 |  |
| Sclerorhynchid | **130,0** | to | **65,5** | 183,0 |  | to | 65,5 |  | 175,6 |  | to | 65,5 |  |
| Squaliformes | **183,0** | to | **0,0** | 270,0 | 183,0 | to | 0,0 |  | 183,0 |  | to | 0,0 |  |
| Squatiniformes | **161,2** | to | **0,0** | 183,0 | 161,2 | to | 0,0 |  | 161,2 |  | to | 0,0 |  |
| Synechodontiformes | **270,0** | to | **61,7** | 270,0 |  | to | 61,7 |  | 270,0 |  | to | 61,7 |  |
| Torpediniformes | **65,5** | to | **0,0** | 270,0 | 183,0 | to | 0,0 |  | 175,6 | 136,4 | to | 0,0 |  |

FAD: First Appearance Datum, LAD: Last Appearance Datum; numerical values in Million years BP, Extreme values for FAD and LAD when approximated (with CBM and DDBM methods and according to phylogenetic relationships considered).
